# Randomised phase II trial of mFOLFOX6 plus bevacizumab versus mFOLFOX6 plus cetuximab as first-line treatment for colorectal liver metastasis (ATOM trial)

**DOI:** 10.1038/s41416-019-0518-2

**Published:** 2019-07-09

**Authors:** Eiji Oki, Yasunori Emi, Takeharu Yamanaka, Hiroyuki Uetake, Kei Muro, Takao Takahashi, Takeshi Nagasaka, Etsuro Hatano, Hitoshi Ojima, Dai Manaka, Tetsuya Kusumoto, Yu Katayose, Toshiyoshi Fujiwara, Kazuhiro Yoshida, Michiaki Unno, Ichinosuke Hyodo, Naohiro Tomita, Kenichi Sugihara, Yoshihiko Maehara

**Affiliations:** 1 Department of Surgery and Science, Graduate School of Medical Sciences, Kyushu University, Fukuoka, Japan; 2 Department of Surgery, Saiseikai Fukuoka General Hospital, Fukuoka, Japan; 3 Department of Biostatistics, Yokohama City University, Yokohama, Japan; 4 Department of Surgical Oncology and Gastroenterology, Tokyo Medical and Dental University, Tokyo, Japan; 5 Department of Clinical Oncology, Aichi Cancer Center Hospital, Nagoya, Japan; 6 Department of Surgical Oncology, Gifu University Graduate School of Medicine, Gifu, Japan; 7 Department of Clinical Oncology, Kawasaki Medical School, Kurashiki, Japan; 8 Department of Hepato-Biliary-Pancreatic Surgery, Hyogo College of Medicine, Nishinomiya, Japan; 9 Department of Surgery, Gunma Prefectural Cancer Center, Ota, Japan; 10 Department of Surgery, Kyoto Katsura Hospital, Kyoto, Japan; 11 Department of Surgery, National Kyushu Medical Center, Fukuoka, Japan; 12 Department of Hepatobiliary and Pancreatic, Tohoku Medical and Pharmaceutical University, Sendai, Japan; 13 Department of Gastroenterological Surgery, Okayama University Graduate School of Medicine, Dentistry, and Pharmaceutical Sciences, Okayama, Japan; 14 Department of Surgery, Tohoku University, Graduate School of Medicine, Sendai, Japan; 15 Division of Gastroenterology, Faculty of Medicine, University of Tsukuba, Tsukuba, Japan; 16 Divison of Lower GI Surgery, Department of Surgery, Hyogo College of Medicine, Nishinomiya, Japan; 17 Kyushu Central Hospital of the Mutual Aid Association of Public School Teachers, Fukuoka, Japan

**Keywords:** Colorectal cancer, Chemotherapy

## Abstract

**Background:**

Chemotherapy with biologics followed by liver surgery improves the resection rate and survival of patients with colorectal liver metastasis (CRLM). However, no prospective study has compared the outcomes of chemotherapy with bevacizumab (BEV) versus cetuximab (CET).

**Methods:**

The ATOM study is the first randomised trial comparing BEV and CET for initially unresectable CRLM. Patients were randomly assigned in a 1:1 ratio to receive mFOLFOX6 plus either BEV or CET. The primary endpoint was progression-free survival (PFS).

**Results:**

Between May 2013 and April 2016, 122 patients were enrolled. Median PFS was 11.5 months (95% CI 9.2–13.3 months) in the BEV group and 14.8 months (95% CI 9.7–17.3 months) in the CET group (hazard ratio 0.803; *P* = 0.33). Patients with a smaller-number but larger-sized metastases did better in the CET group. In the BEV and CET groups, the response rates were 68.4% and 84.7% and the resection rates were 56.1% and 49.2%, respectively.

**Conclusion:**

Although CET achieved a better response rate than BEV for patients with a small number of large liver metastases, both biologics had similar efficacy regarding liver resection and acceptable safety profiles. To achieve optimal PFS, biologics should be selected in accordance with patient conditions.

**Trial registration:**

This trial is registered at ClinicalTrials.gov (number NCT01836653), and UMIN Clinical Trials Registry (UMIN-CTR number UMIN000010209).

## Background

Colorectal cancer (CRC) is the third most common cancer and the second leading cause of cancer death worldwide.^1^ The 5-year overall survival (OS) rate for the curatively resectable stages I–III of CRC is almost 80% but is only 13% for stage IV CRC, which accounts for approximately 18% of CRC diagnoses.^2^ Liver metastases develop in almost 60% of patients with stage IV CRC and in 9–13% of patients after curative resection of CRC.^1,3^ To improve the prognosis of patients with CRC, outcomes for patients with liver metastasis must be improved.

Among the treatment options for colorectal liver metastasis (CRLM), liver resection is the most conducive to a cure, with 5-year OS rates of 29–48%.^4,5^ Even for initially unresectable liver metastasis, effective chemotherapy, along with targeted therapy, sometimes enables resection of liver metastasis; Adam et al. reported a 5-year OS rate of 33% for patients with unresectable metastases who underwent surgery after chemotherapy.^4^ However, it is unclear which type of chemotherapy can best increase resection rates and improve survival in patients with CRLM.

Several promising CRLM treatments have been reported, including chemotherapy and molecular agents that target epidermal growth factor receptor (EGFR) and vascular endothelial growth factor (VEGF).^6–10^ Anti-EGFR drugs resulted in high response and resection rates in the CELIM phase II trial and other studies for initially unresectable CRLM with wild-type *KRAS*.^7,8,11^ Anti-VEGF antibody regimens, such as mFOLFOX6 or CAPEOX plus bevacizumab (BEV), have also showed high response and resection rates in phase II studies.^9,10,12^ These reports suggest that the combination of targeted agents and chemotherapy can increase liver resection rates and response rates, thus improving the progression-free survival (PFS) and OS of patients with CRLM. However, no studies have compared anti-VEGF agents and anti-EGFR agents for liver-limited *RAS*^*wt*^ CRLM.

The present randomised phase II clinical study (the ATOM trial) aimed to compare mFOLFOX6 plus BEV versus mFOLFOX6 plus cetuximab (CET) in patients with liver-limited *RAS*^*wt*^ CRLM.

## Methods

### Patients

The ATOM trial was a multicentre, randomised phase II study designed to evaluate the efficacy and safety of mFOLFOX6 plus BEV and mFOLFOX6 plus CET in patients with liver-limited metastases from wild-type (*K*)*RAS* CRC. Eligible criteria for the study were (1) histopathologically confirmed CRC (adenocarcinoma), excluding appendix and anal cancers; (2) no metastasis other than to liver; (3) tumour tested to be KRASwt (between May 2013 and April 2015) or RASwt (between April 2015 and April 2016) (4) age between 20 and 80 years at the time of enrolment; (5) Eastern Cooperative Oncology Group performance status of 0 or 1; (6) life expectancy of ≥3 months at the time of enrolment; (7) sufficient organ function; and (8) signed informed consent. Furthermore, each patient had to satisfy at least one of the following criteria at enrolment: (a) ≥5 liver metastases, (b) a liver metastasis with a maximum diameter ≥5 cm, (c) technically inappropriate for resection in light of remaining hepatic function, (d) invasion into all hepatic veins or the inferior vena cava, or (e) invasion into both the right and left hepatic arteries or both of the portal veins. The study was approved by the local ethics committee of each participating centre and conducted in 63 Japanese institution.

### Randomisation

Randomisation was based on dynamic allocation by a minimisation method in a centralised web-based system (EPS Corporation, Tokyo, Japan). Allocation factors included: (1) synchronous liver metastases with a primary lesion, synchronous liver metastases without a primary lesion, or metachronous liver metastases; (2) number of metastases (1–4 or ≥5); (3) maximum metastasis diameter (≤5 or >5 cm); and (4) oxaliplatin used as adjuvant chemotherapy.

### Procedures

Patients received either mFOLFOX6 plus BEV (BEV 5 mg/kg, followed by oxaliplatin 85 mg/m^2^, *l*-leucovorin 200 mg/m^2^ and bolus infusion of fluorouracil 400 mg/m^2^ on Day 1 and continuous fluorouracil infusion 2400 mg/m^2^ on Day 1 through Day 2) or mFOLFOX6 plus CET (CET 400 mg/m^2^ as the initial dose and 250 mg/m^2^ as the subsequent doses on Days 1 and 8, followed by mFOLFOX6), no later than 2 weeks after enrolment in the study. Study treatments were continued in 2-week cycles until disease progression. In accordance with the Response Evaluation Criteria In Solid Tumours version 1.1, the same methods were used to perform tumour assessment at baseline and every subsequent 8 weeks using torso contrast-enhanced computed tomography (CT), liver contrast-enhanced magnetic resonance imaging, or whole-body non-contrast CT. The tumour histopathological response rate was defined as the proportion of patients with grade ≥Ib in accordance with the following definition: grade 0, no necrosis in the tumour; grade 1a, necrosis in <33.3% of the tumour; grade 1b, necrosis in 33.3–66.6% of the tumour; grade 2, necrosis in 66.6–<100% of the tumour; and grade 3, necrosis in 100% of the tumour.

Patients underwent liver resections if their metastases were considered resectable, based on tumour assessments performed after receiving at least 8 cycles of either protocol treatment. After liver resection, a total of 12 cycles of the same chemotherapy plus biologic agent as the preoperative treatment were recommended. Surgery was performed at least 42 days after the last dose of BEV.

### Statistical analysis

The primary endpoint was PFS, as assessed by the Independent Central Review Committee (IRC). The cases were not censored at the time of liver resection. Recurrence was considered to be a PFS event in patients who underwent liver resection after protocol treatment. Secondary endpoints included response rate, tumour shrinkage at week 8, liver resection rate, time to treatment failure, OS, quality of life, and adverse events (AEs).

We employed a selection design based on the hazard ratio (HR). For 1-year PFS rates of the two arms to be 55% and 50% (which corresponds to a HR of 0.862 under exponential distribution), we needed 160 patients to select better treatment in terms of an HR with a probability of 75%. Therefore, the initial sample size was 160 in both arms. However, owing to slow accrual of patients, the independent data monitoring committee approved a reduction of the sample size in January 2015 to 120 patients in both arms; this would enable the observation of 84 PFS events, which would indicate a superior treatment with a probability of 70%.

HRs for progression or death for CET versus BEV were estimated using a Cox proportional-hazards model. Survival curves were estimated using the Kaplan–Meier method. Statistical analyses were performed using SAS version 9.4 (SAS Institute, Cary, USA).

## Results

### Patients

**Fig. 1:**
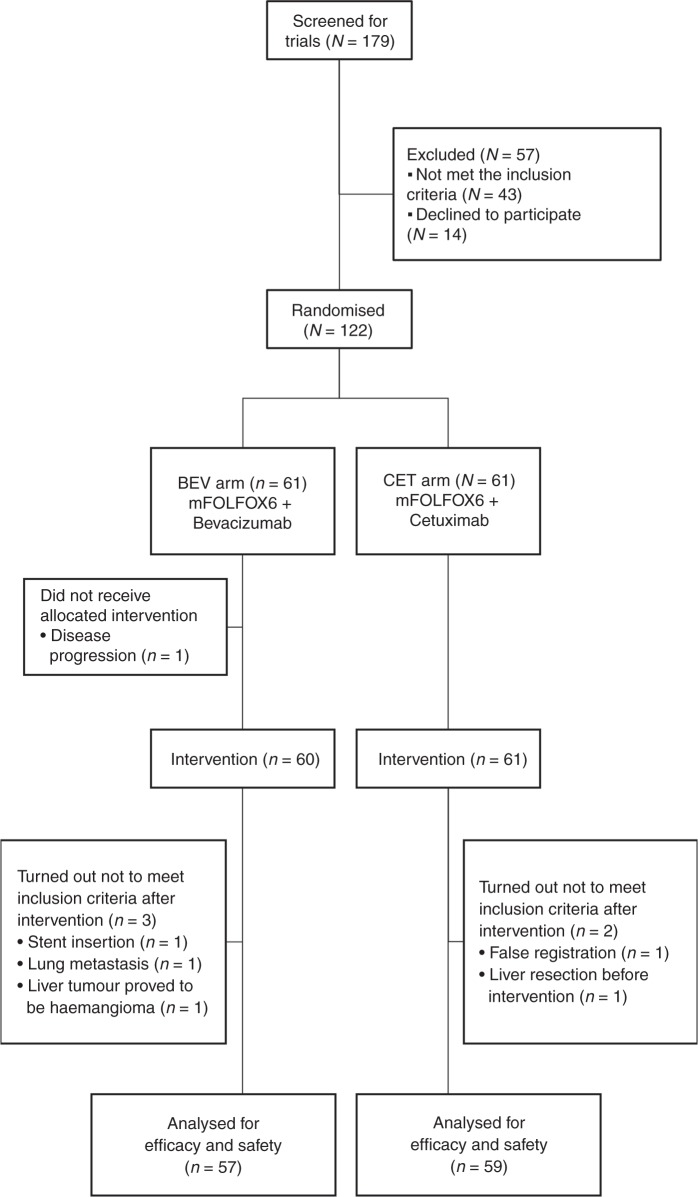
Consort flow diagram. Primary analysis was evaluated in the full analysis set (FAS), which was defined as all patients who were eligible for study inclusion, as well as those who received at least one dose of the protocol treatment

Between May 2013 and April 2016, we screened 179 patients and enrolled 122 patients from 63 sites in Japan (61 patients in each arm). Figure 1 shows the consort flow diagram. Five patients (two CET arm/three BEV arm) did not meet the criteria and a patient in the BEV arm cannot be treated because of rapid disease progression. Patient characteristics were well balanced between the two arms (Table 1). Of the 116 patients, 2 patients in the BEV arm were found to have *RAS* mutations, while 1 patient in each arm did not undergo *RAS* testing due to insufficient tumour samples.

**Table 1:** Patient characteristics

Variables		mFOLFOX6+BEV	mFOLFOX6+CET	*P*
Age (years)	Median (range)	64 (32.0–80.0)	65 (42.0–79.0)	0.328
Sex	Male	34 (59.6%)	34 (57.6%)	0.825
	Female	23 (40.4%)	25 (42.4%)	
ECOG PS	0	51 (89.5%)	51 (86.4%)	0.616
	1	6 (10.5%)	8 (13.6%)	
Adjuvant chemotherapy	Yes	3 (5.3%)	2 (3.4%)	0.619
Prior oxaliplatin	Yes	1 (1.8%)	2 (3.4%)	0.579
Location of tumour	Right	9 (15.8%)	14 (23.7%)	0.284
	Left	48 (84.2%)	45 (76.3%)	
Tumour status	Synchro/primary tumour	13 (22.8%)	15 (25.4%)	0.771
	Synchro/no primary tumour^a^	39 (68.4%)	39 (66.1%)	
	Metachronous	5 (8.8%)	5 (8.5%)	
Number of liver metastases	<5	15 (26.3%)	18 (30.5%)	0.617
	≥5	42 (73.7%)	41 (69.5%)	
Diameter of liver metastases	≤5 cm	20 (35.1%)	19 (32.2%)	0.742
	>5 cm	37 (64.9%)	40 (67.8%)	

*mFOLFOX6* 5-fluorouracil/folinic acid, oxaliplatin, *BEV* bevacizumab, *CET* cetuximab, *ECOG PS* Eastern Cooperative Oncology Group performance status

^a^Synchro/no primary tumour: metastatic tumour diagnosed within 6 months after resection of primary tumour

### Efficacy

**Fig. 2:**
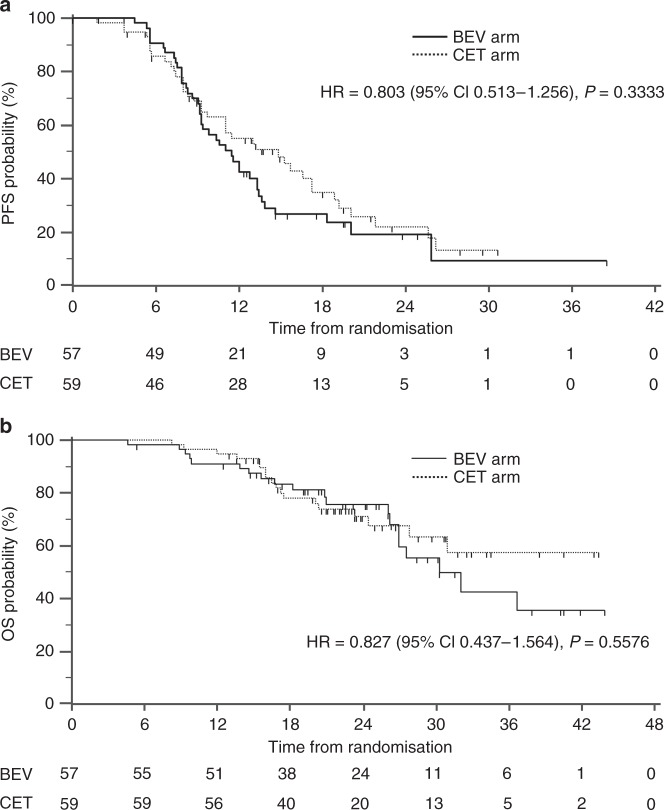
Kaplan–Meier estimates of **a** progression-free survival by central assessment and **b** overall survival. Solid black line: mFOLFOX6+bevacizumab, dotted black line: mFOLFOX6+cetuximab

**Fig. 3:**
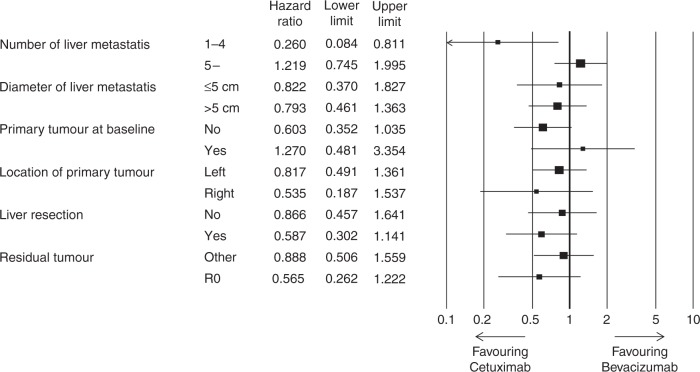
Forest plots show hazard ratios for progression-free survival in patients with colorectal to liver metastases, using mFOLFOX6+bevacizumab and mFOLFOX6+cetuximab

On March 31, 2017, the median follow-up time was 24.3 months. The median PFS assessed by the IRC for the CET arm was 14.8 months (95% confidence interval (CI): 9.7–17.3 months), while for the BEV arm it was 11.5 months (95% CI: 9.2–13.3 months), with a log-rank *P* value of 0.33. The PFS HR between the two arms was 0.803 (95% CI: 0.513–1.256; Fig. 2a); PFS in accordance with the investigators’ evaluations is shown in Supplementary Fig. S1. The median OS in the BEV arm was 30.4 months but was not achieved yet in Cmab arm (HR: 0.827, 95% CI: 0.437–1.564; Fig. 2b). Subgroup analyses for the IRC PFS are shown in Fig. 3 Only in patient with 1–4 liver metastasis the CET arm showed significant HR, whereas PFS was better in the BEV arm among patients with ≥5 metastases. Kaplan–Meier curves of these subgroups are shown in Supplementary Fig. S2A, B.

**Fig. 4:**
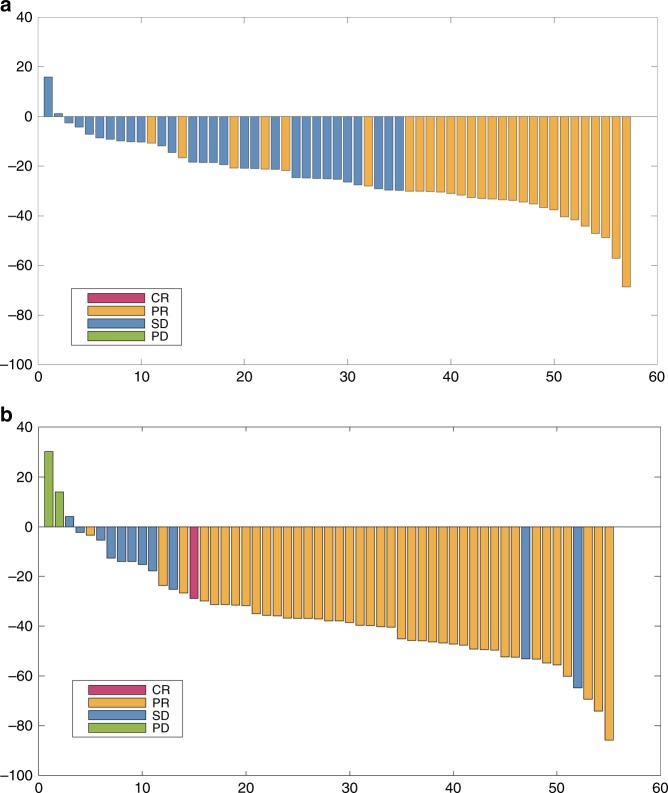
Waterfall plot shows the best change in target lesion size for individual patients by central assessment. CR complete response, NE not evaluable, PD progressive disease, PR partial response, SD stable disease. **a** mFOLFOX6+bevacizumab and **b** mFOLFOX6+cetuximab

The overall response rate (ORR) for BEV was 68.4% (95% CI: 54.8%–80.1%), while the ORR for CET was 84.7% (95% CI: 73.0%–92.8%; *P* = 0.0483; Table 2). Median tumour shrinkage rates at 8 weeks were 25.3% for BEV and 37.8% for CET. The waterfall plot showing the best change in target lesion based on central assessment is shown in Fig. 4. The overall resection rates including R2 were 56.1% (32/57) for BEV and 49.2% (29/59) for CET, and resection rates (R0+R1) were 49.1% (28/57) for BEV and 47.5% (28/59) for CET (Table 2). Median PFS was assessed for patients with R0/R1 status after study treatment followed by surgical resection; median PFS for BEV was 6.5 months (95% CI: 4.0–13.6), while that for CET it was 13.8 months (95% CI: 8.4–not reached), HR: 0.610 (95% CI: 0.298–1.245; Supplementary Fig. S3). Of the 57 tumours that were assessable for histopathological analysis, the tumour histopathological response rate (Grade 1b/2/3) was 66.6% (20/30) for BEV and 92.6% (25/27) for CET (*P* = 0.0229, Supplementary Table S1).

**Table 2:** Overall response rates and liver resection rate

	Overall response rates		*P*
	mFOLFOX6+BEV	mFOLFOX6+CET	
CR (%)	1 (1.8%)	1 (1.7%)	—
PR (%)	38 (66.7%)	49 (83.1%)	—
SD (%)	18 (31.6%)	6 (10.2%)	—
PD (%)	0 (0.0%)	2 (3.4%)	—
NE	0 (0.0%)	1 (1.7%)	—
Overall response rate	68.4%	84.7%	0.0483
Disease control rate	100%	94.9%	0.2437

	Liver resection rate	
	mFOLFOX6+BEV	mFOLFOX6+CET
R0+R1	28 (49.1%)	28 (47.5%)
R0	25 (43.9%)	22 (37.3%)
R1	3 (5.3%)	6 (10.2%)
R2	4 (7.0%)	1 (1.7%)
All	32 (56.1%)	29 (49.2%)
Reasons for R2	Ablation: 2	Ablation and Residual: 1
	Residual: 2	

*BEV* bevacizumab, *CET* cetuximab, *CR* complete response, *mFOLFOX6* 5-fluorouracil/folinic acid, oxaliplatin, *NE* not evaluable, *PD* progressive disease, *PR* partial response, *SD* stable disease

### Safety

Grade ≥3 subjective or objective toxicity events occurred in 40.4% of the patients who received BEV and 52.5% of the patients who received CET. The most frequently occurring AE of grade ≥3 was neutropenia, with an incidence of 36.8% in the BEV arm and 50.8% in the CET arm. Other AEs are presented in Supplementary Table S2. AEs that caused discontinuation occurred in 8 patients (13.1%) in the BEV arm and 6 (9.8%) in the CET arm. No patient died from treatment-related AEs. In the surgical safety population (*n* = 33 in the BEV arm and *n* = 29 in the CET arm), all-grade surgery-related AEs according to the Clavien–Dindo classification were reported in 8 patients (24.2%) in the BEV arm and 12 (41.4%) in the CET arm. The most frequent surgical AE was bile leakage, with an incidence of 18.2% in the BEV arm and 24.1% in the CET arm. No grade 5 AEs were reported (Supplementary Table S3).

## Discussion

The anti-VEGF antibody BEV has an anti-angiogenic effect, which elicits a specific morphologic tumour response of enhanced necrosis, thus increasing the survival benefit of chemotherapy.^13^ Because of these benefits, some pivotal clinical studies reported that combined therapy with cytotoxic drugs could potentially improve CRC prognosis.^10^ A phase II study of patients with CRLM reported that the anti-EGFR antibody CET was associated with an improved response rate and a high liver resection rate.^7,14–17^ Although a few head-to-head randomised studies compared the two antibodies in the whole population of patients with advanced CRC, none of them led to a clear conclusion as to which agent was better for the liver-limited CRLM.^15,17–19^ Our trial aimed to evaluate the effect of an anti-EGFR antibody versus an anti-VEGF antibody in patients with CRLM.

In our analysis, the median PFS (as assessed by the IRC) tended to be better in the CET arm than in the BEV arm, but this difference was not statistically significant. The ORR was also better in the CET arm (84.7%) than the BEV arm (68.4%). However, despite the higher ORR in the CET arm compared with the BEV arm, the overall resection rate was similar in both arms. Although the main results were equivocal, we can consider the results from various angles.

Resection rate is often used as the primary endpoint for phase II studies evaluating chemotherapy for CRLM. Previous studies have reported similar resection rates to those achieved in our study. One study reported a liver resection rate and ORR of FOLFOXIRI+BEV of 51% and 81%, respectively,^10^ while the CELIM trial reported that the resection rates of FOLFOX+CET and FOLFIRI+CET were 51% and 49%, respectively.^7^ Furthermore, a review of data from previous studies reported that response and resection rates were positively correlated in CRLM and found the liver resection rate to be 50–60% when the ORR is 80%.^5^ This correlation is applicable to recent reports, including our study. However, increasing the response rate >80% is a clinical challenge. Therefore, the resection rate might not be an adequate surrogate endpoint in further clinical studies of CRLM.

Subset analysis showed that, among patients with fewer metastases (1–4), the CET group had better survival than the BEV group (HR: 0.260, 95% CI: 0.084–0.811) but conversely among patients with ≥5 metastases. Our inclusion criteria required that patients with ≥5 metastases had tumours ≥5 cm in size. Therefore, the presence of fewer but larger metastases might be a good indication for the use of anti-EGFR antibody. Resection after shrinkage of a large tumour is the ideal clinical course for CET-treated patients. The New EPOC trial could not show any benefit of adding CET to standard chemotherapy in patients with resectable CRLM.^20^ However, this may be because CET is not effective for small resectable liver metastases but seems suitable for patients with fewer but larger metastases.

Tumour sidedness is also an important characteristic of metastatic CRC. Although previous studies indicated no survival benefit for CET in metastatic right-sided CRC,^21^ the PFS of patients with right-sided tumours in our trial showed similar or better results with CET as those with left-sided tumours. CET reportedly reduces tumour size better than BEV, even in right-sided colon cancer.^22^ Therefore, in cases with metastatic CRC limited to the liver, sidedness might not be relevant for PFS, as half of the metastatic site will be surgically resected.

A previous study reported a higher pathological response rate for BEV than for CET.^23^ In contrast, the present study showed a higher pathological response rate for CET than for BEV, which had some effect on the survival of the patients who underwent surgery. In fact, the median PFS of patients with surgical conversion was 13.8 months (95% CI: 8.4–not reached) in the CET arm and 6.5 months (95% CI: 4.0–13.6) in the BEV arm (Supplementary Fig. S3). This difference will have some impact on final overall survival. We will investigate tumour regression grade (TRG), modified TRG, dangerous halo, and morphological response as exploratory endpoints. These analyses will elucidate as to what is the best pathological assessment of prognosis for CRLM.

The present trial was limited by its small sample size, and so the statistical power was insufficient to enable the comparison of the two biologic agents. Furthermore, the OS is immature and needs longer-term follow-up. In addition, the inclusion criteria for initially unresectable CRLM are controversial. We followed the criteria used in previous studies, which included patients with CRLM with <5 metastases but with tumour(s) >5 cm in diameter.^11,12^ Some of these cases might have been resectable without chemotherapy, and this may have confounded the results.

In conclusion, CET showed a higher response rate than BEV among patients with initially unresectable liver-limited metastases and resulted in good PFS, especially for patients with fewer but larger metastases. However, there were no significant differences between BEV and CET in efficacy regarding resection rate, and both biologic agents had acceptable safety profiles. Both agents are viable treatment options for liver metastases with suboptimal resectability.

## Supplementary information

Supplementary Files

Trial Protocol

List of name all institutional committees

## Data Availability

The data sets used and/or analysed during the current study are available from the corresponding author on reasonable request.
