# Supplementary material for: Randomised phase II trial of mFOLFOX6 plus bevacizumab versus mFOLFOX6 plus cetuximab as first-line treatment for colorectal liver metastasis (ATOM trial)
Source: Br J Cancer. 2019 Jul 9;121(3):222–9. doi: 10.1038/s41416-019-0518-2 (PMC6738101; doi:10.1038/s41416-019-0518-2)
Supplement: Supplementary file 1 — Supplementary Files [file 41416_2019_518_MOESM1_ESM.docx]

**FIGURE LEGENDS for supplementary figures and tables**


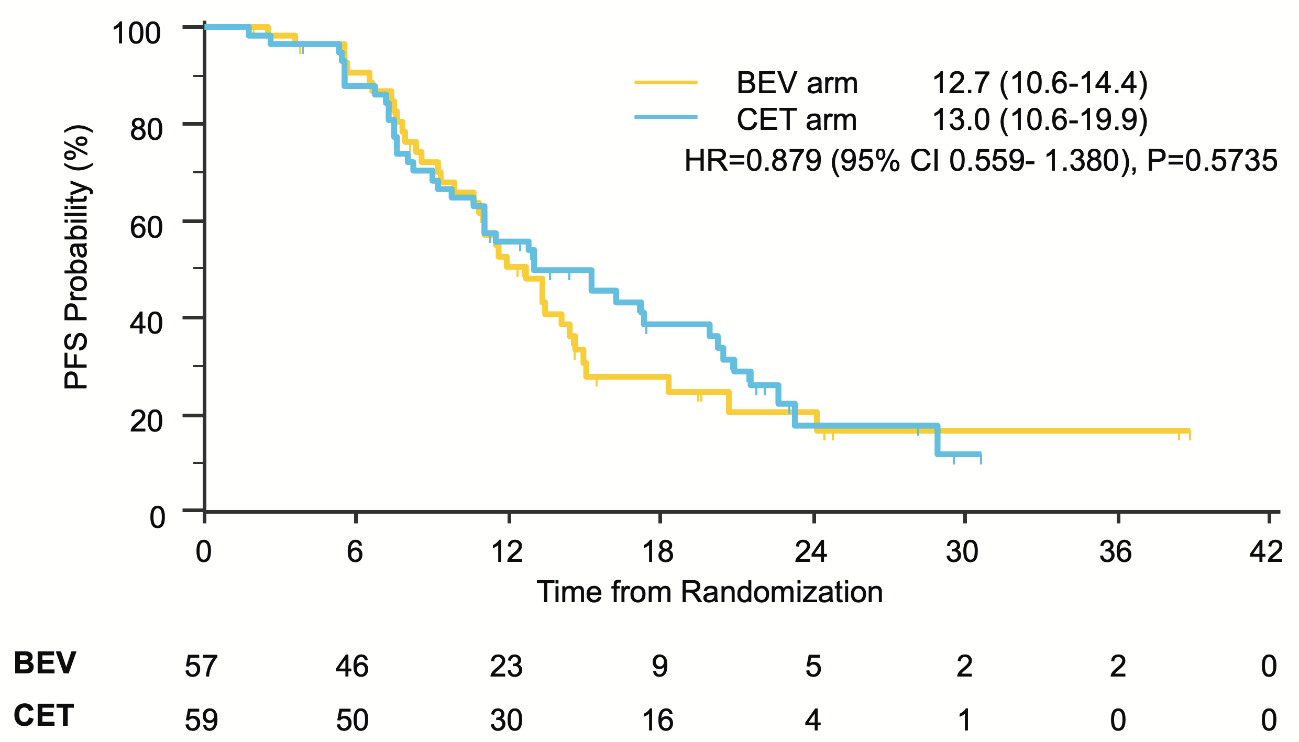


**Supplementary Fig. S1** Kaplan–Meier estimates of progression-free survival from investigator assessments. Yellow line: mFOLFOX6 + bevacizumab, blue line: mFOLFOX6 + cetuximab.


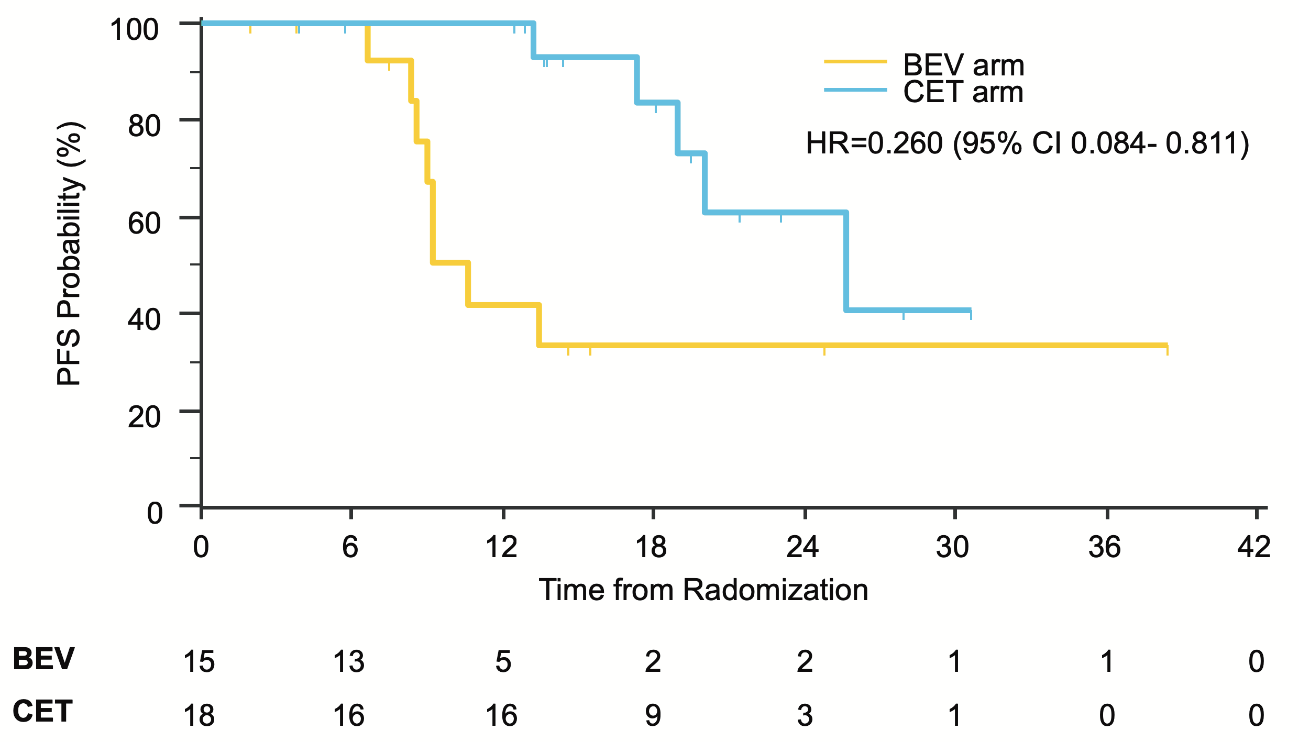


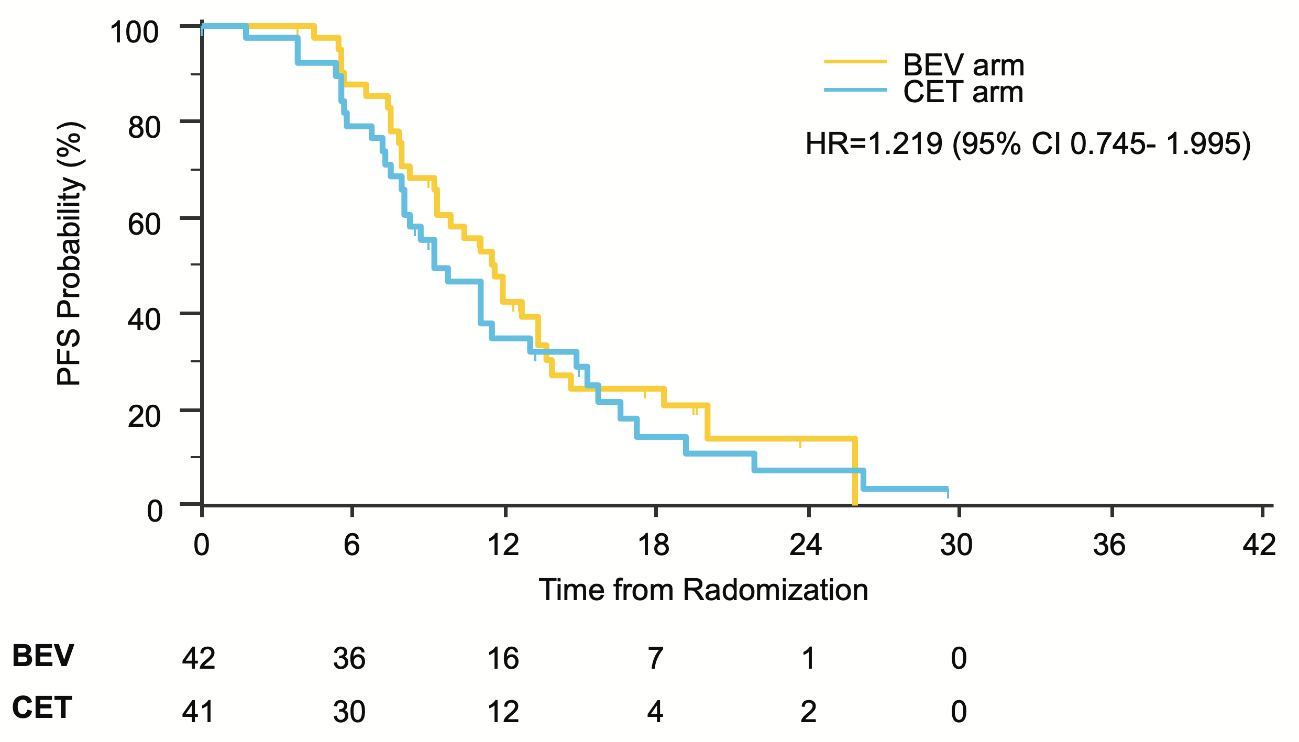


**Supplementary Fig. S2** Kaplan–Meier estimates of progression-free survival by patients with (A) 1–4 tumors or (B) ≥ 5 tumors.

**
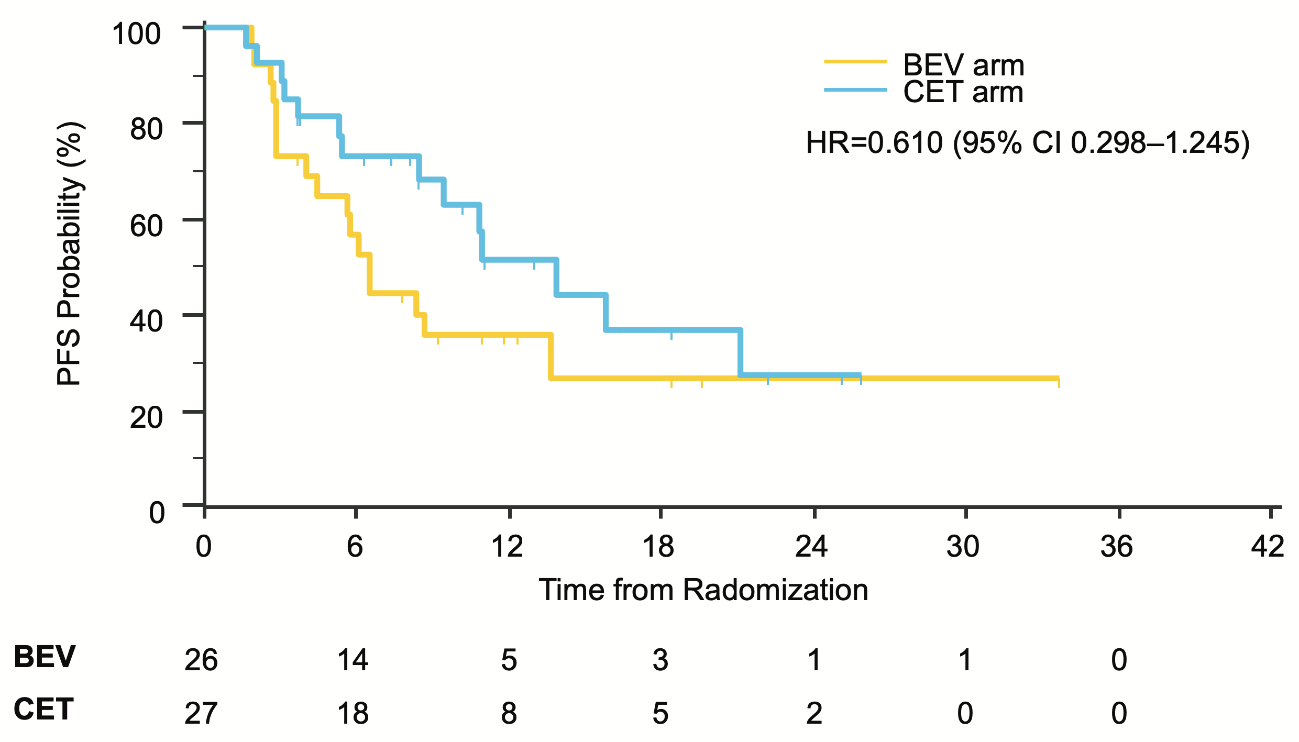
**

**Supplementary Fig.** **S3** Kaplan–Meier estimates of relapse-free survival after first hepatectomy. Yellow line: mFOLFOX6 + bevacizumab, blue line: mFOLFOX6 + cetuximab.

Supplementary Table S1. Pathological responses

| Grade | mFOLFOX6+BEV | mFOLFOX6+CET | Total |
| --- | --- | --- | --- |
|  | 30 | 27 | 57 |
| 0 | 0 (0.0%) | 0 (0.0%) | 0 (0.0%) |
| 1a | 10 (33.3%) | 2 (7.4%) | 12 (21.1%) |
| 1b | 7 (23.3%) | 12 (44.4%) | 19 (33.3%) |
| 2 | 13 (43.3%) | 12 (44.4%) | 25 (43.9%) |
| 3 | 0 (0.0%) | 1 (3.7%) | 1 (1.8%) |

BEV: bevacizumab; CET: cetuximab; mFOLFOX6: 5-fluorouracil/folinic acid, oxaliplatin

Supplementary Table S2. Adverse events (grade 3/4)

|  | mFOLFOX6+BEV | | mFOLFOX6+CET | |
| --- | --- | --- | --- | --- |
|  | *N* | % | *n* | % |
| Neutropenia | 21 | 36.8% | 30 | 50.8% |
| Thrombocytopenia | 1 | 1.8% | 1 | 1.7% |
| Anemia | 2 | 3.5% | 2 | 3.4% |
| Febrile neutropenia | 1 | 1.8% | 0 | 0.0% |
| Mucositis | 1 | 1.8% | 3 | 5.1% |
| Nausea | 1 | 1.8% | 1 | 1.7% |
| Vomiting | 1 | 1.8% | 1 | 1.7% |
| Diarrhea | 1 | 1.8% | 2 | 3.4% |
| Fatigue | 1 | 1.8% | 4 | 6.8% |
| Peripheral neuropathy | 4 | 7.0% | 6 | 10.2% |
| Paronychia | 0 | 0.0% | 9 | 15.3% |
| Dermatitis acneiform | 0 | 0.0% | 9 | 15.3% |
| Hypertension | 9 | 15.8% | 0 | 0.0% |
| Perforation | 0 | 0.0% | 0 | 0.0% |

BEV: bevacizumab; CET: cetuximab; mFOLFOX6: 5-fluorouracil/folinic acid, oxaliplatin

Supplementary Table S3. Surgical adverse events (AEs)

|  | mFOLFOX6 + BEV | | | | mFOLFO6 + CET | | | |
| --- | --- | --- | --- | --- | --- | --- | --- | --- |
|  | All grade | | ≥ Grade 3 | | All grade | | ≥ Grade 3 | |
|  | *N* | % | *n* | % | *n* | % | *n* | % |
| All postoperative AEs | 8 | 24.2% | 4 | 12.1% | 12 | 41.4% | 6 | 20.7% |
| Bleeding | 2 | 6.1% | 0 | 0.0% | 0 | 0.0% | 0 | 0.0% |
| Bile leakage | 6 | 18.2% | 4 | 12.1% | 7 | 24.1% | 5 | 17.2% |
| Biliary abnormality | 1 | 3.0% | 0 | 0.0% | 0 | 0.0% | 0 | 0.0% |
| Ileus | 3 | 9.1% | 0 | 0.0% | 0 | 0.0% | 0 | 0.0% |
| Other GI abnormality | 0 | 0.0% | 0 | 0.0% | 1 | 3.4% | 0 | 0.0% |
| Abscess | 3 | 9.1% | 1 | 3.0% | 3 | 10.3% | 2 | 6.9% |
| Liver abscess | 2 | 6.1% | 0 | 0.0% | 0 | 0.0% | 0 | 0.0% |
| Infection | 0 | 0.0% | 0 | 0.0% | 1 | 3.4% | 0 | 0.0% |
| Other | 0 | 0.0% | 0 | 0.0% | 1 | 3.4% | 0 | 0.0% |

BEV: bevacizumab; CET: cetuximab; GI: gastrointestinal; mFOLFOX6: 5-fluorouracil/folinic acid, oxaliplatin
