# Supplementary material for: Randomised phase II trial of mFOLFOX6 plus bevacizumab versus mFOLFOX6 plus cetuximab as first-line treatment for colorectal liver metastasis (ATOM trial)
Source: Br J Cancer. 2019 Jul 9;121(3):222–9. doi: 10.1038/s41416-019-0518-2 (PMC6738101; doi:10.1038/s41416-019-0518-2)
Supplement: Supplementary file 2 — Trial Protocol [file 41416_2019_518_MOESM2_ESM.pdf]

Randomized Phase ll Study of mFOLFOX6 plus Bevacizumab or mFOLFOX6 plus Cetuximab in Liver-only Metastasis from *KRAS* Wild-Type Colorectal Cancer

Achievement of improved survival by molecular Targeted chemotherapy and liver resection for not Optimally resectable colorectal liver Metastases

ATOM study

Clinical Study Protocol

Principal Investigators

Yoshihiko Maehara

Department of Surgery and Science, Graduate School of Medical Sciences, Kyushu University

Department of Surgery II

3-1-1 Maidashi, Higashi-ku, Fukuoka, Fukuoka Prefecture 812-8582. Tel: 092-642-1151 (main)

Naohiro Tomita

Division of Lower Gastrointestinal Surgery, Department of Surgery, Hyogo College of Medicine

1-1 Mukogawa-cho, Nishinomiya, Hyogo Prefecture 663-8501. Tel: 0798-45-6111 (main)

Ichinosuke Hyodo

Gastroenterology, Graduate School of Comprehensive Human Sciences, Institute of Clinical Medicine, University of Tsukuba

1-1-1 Tennodai, Tsukuba, Ibaraki Prefecture 305-8577. Tel: 029-853-3109 (direct)

Michiaki Unno

Division of Gastroenterological Surgery, Tohoku University Graduate School of Medicine

1-1 Seiryo-machi, Aoba-ku, Sendai, Miyagi Prefecture 980-8674. Tel: 022-717-7000 (main)

Study Secretariat - Responsible Person

Yasunori Emi

Department of Surgery and Science, Graduate School of Medical Sciences, Kyushu University

Department of Surgery II

Chair, Protocol Preparation Committee

Kei Muro

Department of Clinical Oncology, Aichi Cancer Center Hospital

Data Center

Data Management Center 1, Clinical Information Division, EPS Corporation

2-3-19 Koraku, Bunkyo-ku,Tokyo 112-0004. Tel: 03-5684-7767

E-mail: prj-atomdc@eps.co.jp

Ver. 1.0, 17 December 2012
Ver. 1.1, 14 November 2013
Ver. 1.2, 14 July 2014
Ver.2.0, 16th Jan, 2015

0. Synopsis

<table>
<tr><td>Protocol Title</td><td>Randomized Phase ll Study of mFOLFOX6 plus Bevacizumab or mFOLFOX6 plus Cetuximab in Liver-only Metastasis from KRAS Wild-Type Colorectal Cancer</td></tr>
<tr><td>Schema</td><td>

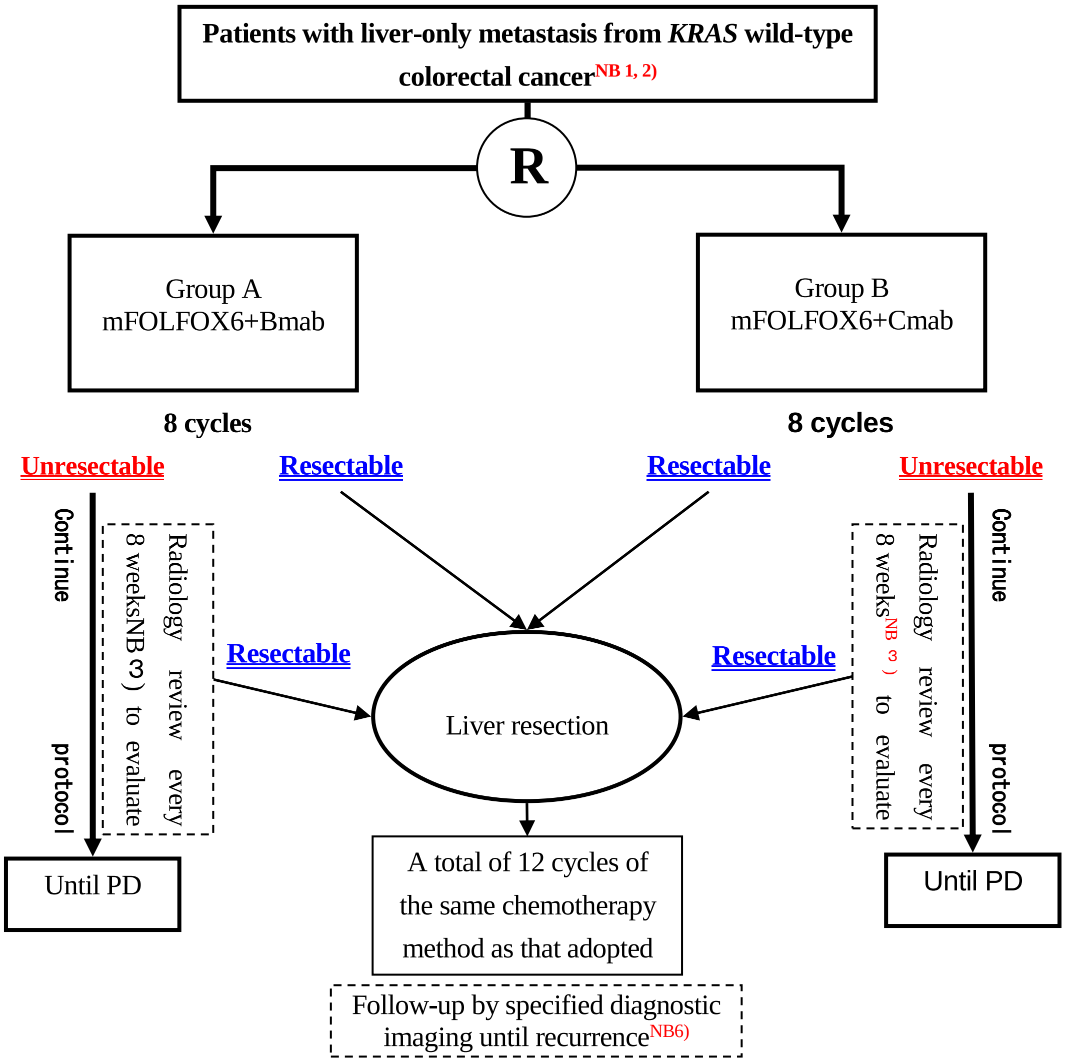

Bmab = bevacizumab, Cmab = cetuximab; PD = progressive disease

NB1: RAS test for wild-type status should confirm the presence of mutations in at least KRAS Codons 12 and 13 (Exon 2). RAS test (except for KRAS Codons 12 and 13 (Exon 2)) is not considered for registration. In case of assessment for KRAS Codons 59, 61 and 146 (Exon 3 and 4) and NRAS Exon 2, 3, 4 and etc, all status are confirmed to be wild type.

NB2: If the endscpe can not pass the primary tumor, enrollment should follow primary resection or creation of colostomy. In this case, patients will be regisotred after 14 days from finishing surgery.

NB3: The radiology review every eight weeks can be performed within a time window of plus or minus one week.

NB4: The diagnostic imaging method to be used will be contrast-enhanced CT of the trunk (chest, upper abdomen, pelvis) If contrast-enhanced CT is not possible for reasons such as allergy to iodine contrast agents, unenhanced CT of the trunk plus liver (contrast-enhanced) MRI will be performed. For the radiology review in such circumstances, evaluations will be continued using the same diagnostic imaging method as that adopted at baseline (the scanning method used before enrollment).

NB5: For protocol chemotherapy delivered beyond eight cycles too, radiology review will be performed every eight weeks (±one week). After each review, liver resectability will be evaluated, and if the subsubinvestigator decides that it is possible, liver resection can proceed.

NB6: Patients who undergo liver resection will be followed until new lesions (including recurrence) are evident, and response will be assessed by the diagnostic imaging method used at enrollment. As a general rule, this will be done every 12 weeks (±one week) in the first year, every 16 weeks (±one week) in the second year, and every 24 weeks (±one week) in the third year after resection for patients with R0 and R1 resections, and every eight weeks (±one week) for patients with R2 resections (counting from the day of liver resection).

</td></tr>
</table>

Objectives	To evaluate the efficacy and safety of mFOLFOX6 plus bevacizumab and mFOLFOX6 plus cetuximab in patients with liver-only metastasis from *KRAS* wild-type colorectal cancer.<br><br>Primary endpoint: Progression-free survival (PFS); central radiology review<br><br>Secondary endpoints: Response rate (RR)<br>Tumor shrinkage rate at eight weeks<br>Liver resection rate<br>R0 liver resection rate (pathologic diagnosis)<br>Progression-free survival (PFS); subinvestigator review<br>Time to treatment failure (TTF)<br>Overall survival (OS)<br>Quality of Life (QoL)<br>Incidence of adverse events (chemotherapy-related, surgery-related)<br><br>Exploratory endpoints: Tumor regression grade (TRG)<br>Modified tumor regression grade (mTRG)<br>Dangerous halo (HALO)<br>Sinusoidal obstruction syndrome (SOS)<br>Exploratory histopathological investigations as required, in addition to TRG, mTRG, HALO, and SOS<br>Spleen volume index (SVI), Morphologic Response
Subjects	Eligibility Criteria<br>Patients who meet all of the following criteria will be eligible for enrollment in the study. Both men and women may be enrolled.<br>1. Histopathologically confirmed colorectal cancer (adenocarcinoma). Appendix cancer and anal cancer are excluded.<br>2. *KRAS* wild type[NB)].<br>NB: *RAS* test for wild-type status should confirm the presence of mutations in at least *KRAS* Codons 12 and 13 (Exon 2). *RAS* test (except for *KRAS* Codons 12 and 13 (Exon 2)) is not considered for registration. In case of assessment for *KRAS* Codons 59, 61 and 146 (Exon 3 and 4) and *NRAS* Exon 2, 3, 4 and etc, all status are confirmed to be wild type.<br>3. Synchronous or metachronous liver-only metastasis, with no history of or coexisting extrahepatic distant metastasis or recurrence.<br>However, for cases of synchronous liver metastasis with the primary lesion present at enrollment, the primary lesion must be no more than two-thirds of the circumference and be resectable. If the primary lesion is greater than two-thirds of the circumference, the patient can be enrolled after resection.<br>NB: If the endscpe can not be passed to the primary tumor, enrollment should follow primary resection or creation of colostomy. In this case, patients will be regisotred after 14 days from finishing surgery.<br>NB: If the endscope cac be passed to permary tumor, patients will be registered without primary tumor resection. If the primary tumor resection will be performed, patients will be regisotred after 14 days from finishing surgery.<br>NB: In case patient who had intestinal obstruction and performed colostmy in synchronous liver limited mCRC at the time of registration can be enrolled after 14 days from finishing surgery. (in case of stent placement, patient can not be enrolled)<br>NB: Superior mesenteric lymph node metastases (#214) and periaortic lymph node metastases (#216) are not included in the primary regional lymph nodes. Patients with positive hepatic hilar lymph node metastases are not included in those with liver-only metastases.<br>NB: Synchronous multiple colorectal cancers that fall within the scope specified in the protocol can be enrolled (curative resection possible)<br>NB: Defined specifically in this study<br>4. Patients with at least one measurable lesion (RECIST Ver. 1.1) with long axis length 1 cm or more on diagnostic imaging (contrast-enhanced CT or contrast-enhanced liver MRI) that can be

continuously evaluated in accordance with the protocol requirements, who meet any of the following criteria[NB)].

1) Five or more liver metastases.
2) Liver metastasis with maximum diameter exceeding 5 cm.
3) Technically unresectable, in light of remaining hepatic function.
4) Invasion into all hepatic veins or inferior vena cava.
5) Invasion into both right and left hepatic arteries or portal veins.

NB: Standards for difficult-to-resect liver metastases from colorectal cancer defined by the Protocol Preparation Committee in this study.

NB: The diagnostic imaging method which is assessed for liver limited metastasis should be contrast CT (chest, abdomen, pelvis). If contrast CT will not be arranged due to iodine contrast media allergy, non-contrast CT (abdominal) + MRI (liver) should be arranged and the same diagnostic imaging method will be reccomeded for futher tumor assessment.

5. No prior chemotherapy (including hepatic arterial infusion) for colorectal cancer.
Adjuvant chemotherapy and neoadjuvant chemo-radiotherapy (excluding patients with synchronous liver metastases from lower rectal cancer) are not included in the chemotherapies listed above (however, if oxaliplatin-containing adjuvant chemotherapy has been given, 24 weeks must have elapsed after the last dose of oxaliplatin.
6. No history of surgical resection, radiofrequency ablation or other thermal coagulation therapy, cryotherapy, chemotherapy, or other treatment for liver metastases or other organ metastases.
7. Age at enrollment ≥20 y and ≤80 y.
8. Eastern Cooperative Oncology Group (ECOG) performance status of 0 or 1.
9. Life expectancy of three months or longer from date of enrollment.
10. Major organ function that meets the criteria below within 14 days before enrollment. If multiple test results are available within the particular period, those obtained nearest the time of enrollment will be used, and blood transfusions and use of hematopoietic growth factors or similar products are not permitted within 14 days before the day of testing.
    1) Neutrophil count ≥1,500/mm^3
    (Calculated from the white blood cell count and the neutrophil ratio (%) in the differential white blood cell count)
    2) Platelet count ≥10.0×10^4 /mm^3
    3) Hemoglobin ≥9.0 g/dl
    4) Total bilirubin ≤2.0 mg/dl
    5) AST, ALT ≤200 IU/I
    6) Serum creatinine ≤1.5 mg/dl
    7) INR <1.5
    8) Urinary protein ≤2+[NB)]

NB: See the chart below for conversion of proteinuria grade, and qualitative and quantitative values.

Grade	CTCAE v4.0	
	Qualitative	Quantitative
1	1+	<1.0g/24h
2	2+	1.0-3.5g/24h (adult)
3	−	≥3.5g/24h (adult)

11. Personally signed and dated informed consent forms for participation in this study, obtained before enrollment.

Exclusion criteria

Patients who meet any of the following criteria will be excluded from the study.

1. History of serious drug hypersensitivity.
2. Receiving treatment with anti-platelet drugs (aspirin products with daily dose ≥325 mg) or NSAIDs[NB)].

NB: As-needed use of NSAIDs for cancer pain is permitted.

	3. Continuous use of systemic steroids.<br>4. Surgery or biopsy with skin incision, or traumatic injury sutured within 14 days before enrollment.<br>Excludes sutures used for securing an implantable venous port.<br>5. Serious postoperative complications. (e.g., postoperative infection, ruptured sutures, or paralytic ileus that has not resolved by enrollment)<br>6. An unequivocal diagnosis of hereditary colorectal cancer[NB)].<br>NB: Familial adenomatous polyposis (FAP) or hereditary non-polyposis colorectal cancer (HNPCC)<br>7. Active double cancer[NB)].<br>NB: Synchronous double cancer or metachronous double cancer with a disease-free period of five years or less. However, carcinoma in situ found to have been cured by local treatment or lesions corresponding to intramucosal carcinoma are not included in active double cancers. Multiple colorectal cancers are permitted if they are all adenocarcinomas. Cancers with histologic type other than adenocarcinoma will be excluded.<br>8. Concurrent or previous cerebrovascular disease symptoms within one year before enrollment.<br>9. Ascites, or pleural or cardiac effusion requiring drainage.<br>10. Fresh hemorrhage from the gastrointestinal tract, intestinal paralysis, intestinal obstruction, or peptic ulcer.<br>11. Concurrent or previous gastrointestinal perforation within one year before enrollment.<br>12. Active infection.<br>13. HBs antigen positive or HCV antibody positive[NB)].<br>NB: HBs and HCV tests are essential.<br>14. Any of the following coexisting diseases.<br>1) Poorly controlled hypertension<br>2) Poorly controlled diabetes mellitus<br>3) Poorly controlled arrhythmia<br>4) Other clinically significant disease (e.g., heart disease, interstitial pneumonia, renal failure)<br>15. Grade 2 or higher diarrhea.<br>16. Grade 1 or higher peripheral neuropathy.<br>17. Pregnant women, nursing mothers, women of childbearing potential (intention), and men whose partners wish to become pregnant.<br>18. Concurrent psychiatric illness or symptoms that may hinder participation in the study.<br>19. Judged by the subinvestigator to be unsuitable for entry into this study.
Planned number of subjects<br>Planned enrollment period<br>Planned follow-up period<br>Planned overall study period	Two groups: 120 subjects (60 subjects in each)<br>Enrollment period: May 2013 to April 2016 (three years)<br>Follow-up period: one years after the end of enrollment<br>Overall study period: May 2013 to April 2017 (four years)

Contact for further inquiries	Contact for inquiries of a medical nature.<br>Study Secretariat: Responsible person Yasunori Emi<br>Satomi Abe<br>E-mail：abesato@surg2.med.kyushu-u.ac.jp<br>Department of Surgery and Science, Graduate School of Medical Sciences, Kyushu University<br>Department of Surgery II<br>3-1-1 Maidashi, Higashi-ku, Fukuoka, Fukuoka Prefecture 812-8582.<br>Tel: 092-642-5466<br><br>Contact for non-medical inquiries<br>Data center: Responsible person Tatsumi Shimizu<br>DM Center, Clinical Information Division, EPS Corporation<br>2-3-19 Koraku, Bunkyo-ku,Tokyo 112-0004<br>Tel: 03-5684-7767<br>E-mail: prj-atomdc@eps.co.jp<br>Monday to Friday, 0900 to 1700<br>(excluding Saturdays, Sundays, national holidays, and December 29 to January 4)

1. Objectives

To evaluate the efficacy and safety of mFOLFOX6 plus bevacizumab and mFOLFOX6 plus cetuximab in patients with liver-only metastasis from *KRAS* wild-type colorectal cancer.

Primary endpoints: Progression-free survival (PFS); central radiology review

Secondary endpoints: Response rate (RR)
Tumor shrinkage rate at 8 weeks
Liver resection rate
R0 liver resection rate (pathologic diagnosis)
Progression-free survival (PFS); subinvestigator review
Time to treatment failure (TTF)
Overall survival (OS)
Quality of Life (QoL)
Incidence of adverse events (chemotherapy-related, surgery-related)

Exploratory endpoints: Tumor regression grade (TRG)
Modified tumor regression grade (mTRG)
Dangerous halo (HALO)
Sinusoidal obstruction syndrome (SOS)
Exploratory histopathological investigations as required, in addition to TRG, mTRG, HALO, and SOS, Spleen volume index (SVI), Morphologic Response

2. Background and Rationale for Study Plan

2.1. Subjects

2.1.1. Epidemiology

Morbidity and mortality rates for colorectal cancer are increasing in Japan. According to Cancer Statistics 2010[1] published by the Foundation for Promotion of Cancer Research, more women die from colorectal cancer than from any other malignant neoplasm, and among men, it is third most common cause of death from malignancy, after lung cancer and gastric cancer.

The five-year survival rate for curatively resectable Stages I to III colorectal cancer is nearly 80%, but the five-year survival rate for Stage IV colorectal cancer, which accounts for about 18% of cases, is an unsatisfactory 13%[2]. Among Stage IV colorectal cancers, liver metastases develop in just under 60% of cases (Table 1)[2]. By contrast, liver recurrence occurs in 9% to 13% of cases after curative resection of colorectal cancer (Table 2)[2]. To improve the prognosis for patients with colorectal cancer, it is vital to improve these therapeutic outcomes for liver metastasis.

Table 1. Frequencies of Synchronous Distant Metastases From Colorectal Cancer (data for 4,746 cases of synchronous distant metastases from colorectal cancer from among 26,091 cases of colorectal cancer[2]

	Liver	Lungs	Peritoneum	Other sites				
				Bone	Brain	Virchow	Other	Total
Frequency (%)	57.5%	8.9%	27.5%	1.7%	0.4%	0.4%	2.5%	5.0%
No. of patients	2,779	422	1,307	80	17	20	121	238

Table 2. Recurrence Rate by Site After Curative Resection of Colorectal Cancer (among 5,230 patients with

curative resection of colorectal cancer)[2)]

	Liver	Lungs	Local	Anastomotic site	Other
Recurrence rate (%)	7.1%	4.8%	4.0%	0.4%	3.8%
No. of patients	373	250	209	22	199

The Colorectal Cancer Study Group has proposed a classification scheme for liver metastases, which combines findings on the presence or absence of liver metastases, and the number and size of metastases (Table 3)[2)]. Analysis of registry cases by a study group funded by MHLW grants-in-aid for cancer research (Kato Group)[4)] using this classification scheme shows that the percentages of patients undergoing liver resection in categories H2 and H3 are smaller (Table 4) and that the prognoses are poorer than that in H1 (Figure 1).

Table 3. Classification of Liver Metastases[2)]

HX: Liver metastasis cannot be assessed.

H0: No liver metastasis

H1: One to four metastatic tumors, all of which are 5 cm or less in maximum diameter

H2: Other than H1 or H3.

H3: Five or more metastatic tumors at least one of which is more than 5 cm in maximum diameter.

Table 4. Liver Resection Rate[3)]

	H1		H2		H3	
No. of patients	321		151		16	
No. with liver resection	294	91.6%	120	79.5%	6	37.5%
No. without liver resection	27	8.4%	31	20.5%	10	62.5%

Figure 1. Survival Curves (H factor)[4)]

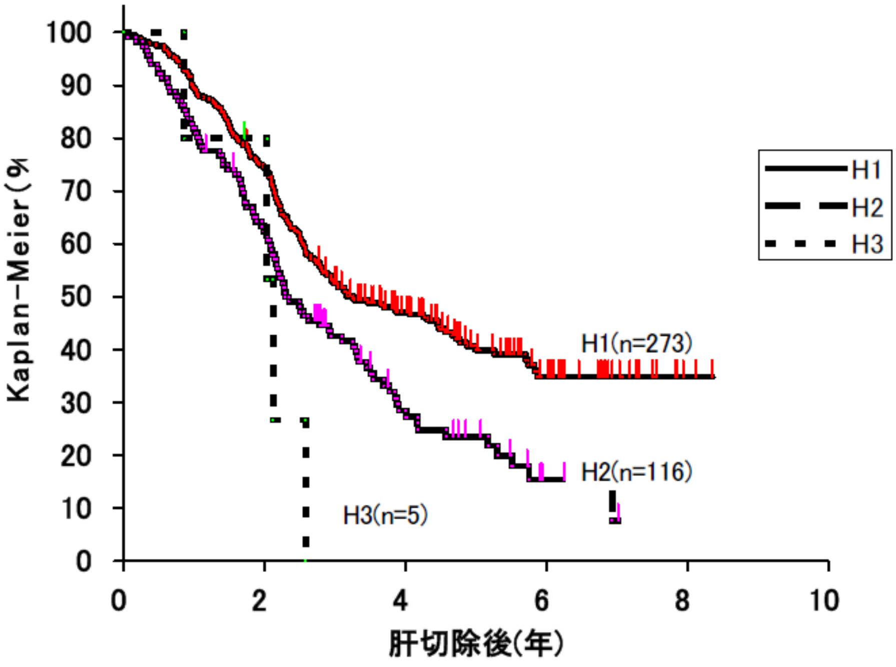

2.2. Standard Therapies for the Target Disease

The treatment options for liver metastases from colorectal cancer include liver resection, coagulation therapy, hepatic arterial infusion chemotherapy, and systemic chemotherapy. The five-year survival rates following curative liver resection are reported to be 29% to 48% (Figure 2)[4, 5], and the most definitive therapy which promises a cure is liver resection.

According to the guidelines for the treatment of colorectal cancer, curative liver resection is recommended if the liver can be resected without leaving residual metastases, if the primary tumor is controlled or can be controlled, if there are no extrahepatic metastases or they can be controlled, and if remnant liver function can be preserved after resection[2].

The NCCN guidelines[6] indicate that the treatment options for liver or lung-limited synchronous metastases depend on their resectability. Similar guidelines are applicable for metachronous cancers.

For unresectable liver or lung-only synchronous metastases, the recommendation is for FOLFOX/XELOX/FOLFIRI with or without bevacizumab, FOLFOX/FOLFIRI with or without panitumumab, or FOLFIRI with or without cetuximab (for *KRAS* wild type only), or FOLFOXIRI, followed by evaluations for resectability every two months; if the metastases are assessed as resectable, surgery is performed.

In cases of unresectable metachronous metastases, the recommended treatment for recurrence within one year after postoperative adjuvant chemotherapy using FOLFOX is FOLFIRI plus bevacizumab, panitumumab, or cetuximab (for *KRAS* wild type only), and for subsequent recurrence, the recommendation is for chemotherapy according to the standard regimen for advanced or recurrent colorectal cancer. If liver resection is deemed possible following evaluations of resectability every two months, it is recommended that liver resection be performed.

The ESMO guidelines[7] propose that treatment selection be guided by the treatment intensity deemed necessary in advanced or recurrent colorectal cancer (Tables 5, 6). In cases of wild-type liver-only disease, the recommendation is for two-drug combination chemotherapy plus bevacizumab or cetuximab, and if the metastases are found to be resectable, the recommendation is to proceed with their resection.

In Europe, there is a consensus proposed by Nordlinger and colleagues in the European Colorectal Metastases Treatment Group[8]. Disease is categorized as resectable, not optimally resectable, or unresectable. Not optimally resectable is defined as "difficult to resect for technical reasons (proximity to hepatic vein, portal vein branches" or "technically possible to resect, but oncologically problematic (number of liver metastases greater than 4, maximum diameter 5 cm or more, synchronous liver metastases, primary lymph node metastasis positive, high levels of tumor markers)". Chemotherapy in combination with molecular targeted drugs is recommended, followed by curative resection if a response is achieved.

Table 5. Clinical Groups for First-line Treatment Stratification [7]

Group	Clinical presentation	Treatment aim	Treatment intensity
0	Clearly R0-resectable liver and/or lung metastases	• Cure, decrease risk of relapse	Nothing or moderate (FOLFOX)
1	Not R0-resectable liver and/or lung metastases only Which<br>• Might become resectable after response to induction chemotherapy<br>• ±Limited/localized metastases to other sites, e.g. locoregional lymph nodes<br>• Patient is physically able to undergo major surgery (biological age, heart/lung condition) and more intensive	• Maximum tumor shrinkage	Upfront most active combination regimen

	Chemotherapy		
2	Multiple metastases/sites, with<br>• Rapid progression and/or<br>• Tumor-related symptoms and/or risk of rapid deterioration<br>• Co-morbidity allows intensive treatment	• Clinically relevant tumor shrinkage as soon as possible<br>• At least achieve control of progressive disease	Upfront active combination: at least doublet
3	Multiple metastases/sites, with<br>• Never option for resection<br>• and/or no major symptoms or risk of rapid deterioration<br>• and/or severe comorbidity (excluding from later surgery and/or intensive systemic treatment, as for groups 1 + 2)	• Abrogation of further progression<br>• Tumor shrinkage less relevant<br>• Low toxicity most relevant	Treatment selection according to disease characteristics and patients preference re toxicity and efficacy:<br>• “Watchful waiting” (exceptional)<br>• Sequential approach: start with<br>• Single agent, or<br>• Doublet with low toxicity<br>• Exceptional triplets

Table 6. Options for First-line Treatment According to Clinical Groups and Grading (defined by treatment aim, available data and expert recommendation)[7)]

Group	*KRAS* wild-type	Recommendation[a]	*KRAS* mutant	Recommendation[a]
1	FOLFIRI + Cet	+++	FOLFOX/XELOX + Bev	+++
	FOLFOX + Pan/Cet	+++	FOLFOXIRI	++(+)[b]
	FOLFOX/XELOX + Bev	++(+)	FOLFIRI/XELIRI + Bev	++(+)[c]
	FOLFOXIRI	++(+)[b]	FOLFOX/XELOX	+
	FOLFIRI/XELIRI + Bev	++(+)[c]	FOLFIRI/XELIRI	+
	FOLFOX/XELOX	+	IRIS	+
	FOLFIRI/XELIRI	+		
	IRIS	+		
2	FOLFIRI + Cet	+++	FOLFOX/XELOX + Bev	+++
	FOLFOX + Pan	+++	FOLFIRI/XELIRI + Bev	++(+)[c]
	FOLFOX/XELOX + Bev	+++	FOLFOX/XELOX	++
	FOLFIRI/XELIRI + Bev	++(+)[c]	FOLFIRI/XELIRI	++
	FOLFOXIRI	+(+)[b]	FOLFOXIRI	++[b]
	FOLFOX + Cet	+(+)	IRIS	+
	FOLFOX/XELOX	+		
	FOLFIRI/XELIRI	+		
	IRIS	+		
3	FUFOL/Cape (mono)	+++	FUFOL/Cape (mono)	+++
	FUFOL/Cape + Bev	+++	FUFOL/Cape + Bev	+++
	XELOX/FOLFOX	++	XELOX/FOLFOX	++
	FOLFIRI/XELIRI	++	FOLFIRI/XELIRI	++
	IRIS	+	IRIS	+
	Cet/Pan (mono)	(+)	watchful waiting	+ selected pts.[d]
	watchful waiting	+ selected pts.[d]	triplets (±Bev)	+ option for spec. situations[e]
	triplets (+/−Bev or Cet/Pan)	+ option for spec. situations[e]		

a Consented recommendation, however decision might be modified based on individual objective and subjective parameters.
b FOLFOXIRI: only two (small) phase III studies with contradictory results.

c No randomized data for FOL(XEL)IRI + Bev.
d Option in case of low tumor burden, asymptomatic, indolent disease: close control until definitive progression (not until symptoms!).
e Patients who are group 3 but deserve (and tolerate) more intensive treatment due to specific reasons.
XELIRI, capecitabine + irinotecan; IRIS, irinotecan + S1.

According to the report by Adam et al., the five-year survival rate is 33% in patients who undergo liver resection after an initial assessment of unresectable, followed by chemotherapy to enable curative resection, versus a five-year survival rate of near zero for patients who do not undergo liver resection[9)] (Figure 2)[4)]. In a clinical study in patients with either liver-only metastases from colorectal cancer or advanced or recurrent colorectal cancer, Folprecht et al. found that response rate was strongly associated with the resection rate for metastases (liver-only metastases from colorectal cancer, $p=0.002$; advanced or recurrent colorectal cancer, $p<0.001$) (Figure 3)[5)]. However, this does not include clinical studies of concomitant treatment with molecular targeted drugs.

Additionally, in a 2009 paper in J Clin Oncol, Kopetz et al.[10)] reported that improvements in the liver resection rate since the arrival of oxaliplatin in 1998 and the emergence of molecular targeted drugs in 2004 have contributed substantially to improved overall survival in advanced and recurrent colorectal cancer. By contrast, according to the results of the LiverMetsurvey reported at the 2011 ASCO annual meeting by Adam et al[11)], molecular targeted drugs have not contributed to overall survival after curative liver resection.

Figure 2. Survival for patients who underwent resection at initial presentation (n=335) and patients with initially unresectable disease who underwent resection after chemotherapy (n=138)[5)]

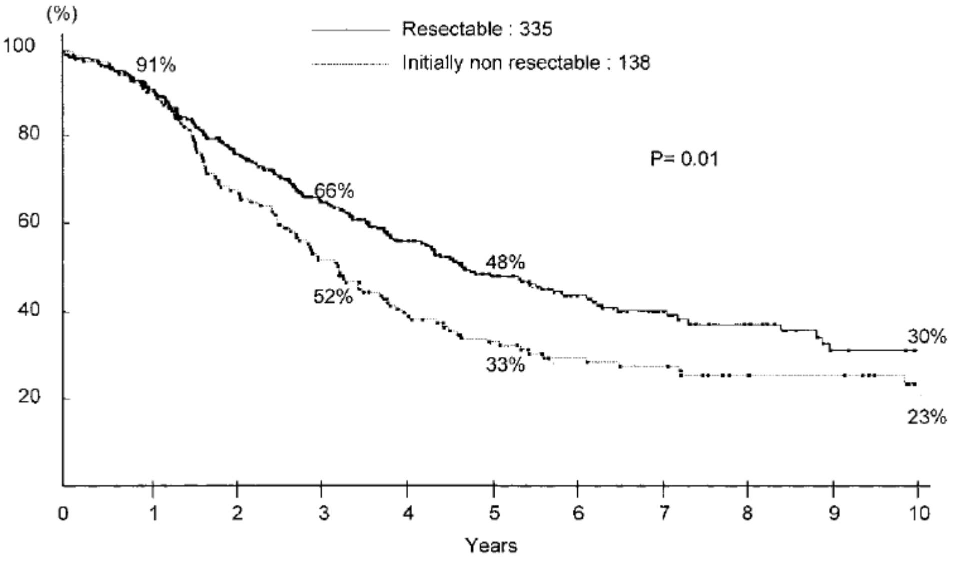

Figure 3. Relationship between post-chemotherapy liver resection rate and response rate

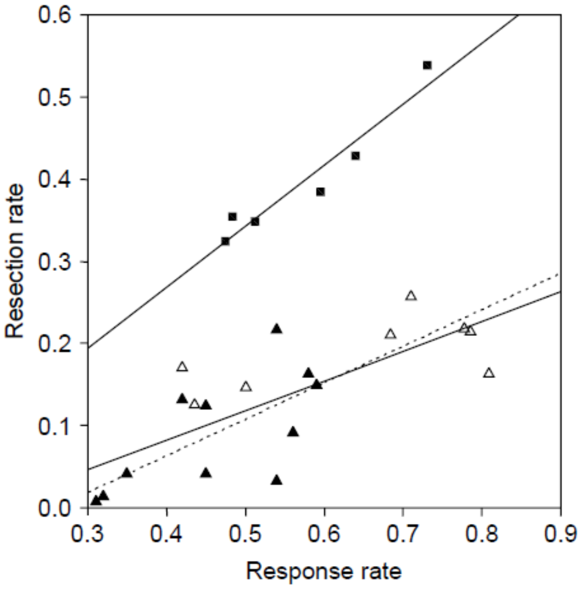

■ Clinical studies for liver-only metastases (r=0.96; p=0.002)

△▲ Clinical studies for advanced or recurrent colorectal cancer (Solid line: r=0.74; p<0.001)

▲ Phase III clinical studies for advanced or recurrent colorectal cancer (Dotted line: r=0.67; p=0.024)

2.2.1. Clinical Studies for Resectable Liver Metastases from Colorectal Cancer

The usefulness of neoadjuvant chemotherapy (perioperative chemotherapy) for patients with colorectal cancer with up to four liver metastases was verified in study EORTC 40983[12)]. In total, 364 patients were randomly assigned to treatment with either six cycles of pre- and post-operative FOLFOX4 (n=182) or resection only (n=182). In the ITT analysis, the 3-year progression-free survival rates in the perioperative chemotherapy group and resection-only group were not significantly different: 35.4% and 28.1% (hazard ratio=0.79; p=0.058). However, the survival rates were 36.2% and 28.1% (hazard ratio=0.77; p=0.041) in eligible patients, and 42.4% and 33.2% (hazard ratio=0.73; p=0.025) in patients who underwent resection, demonstrating that prognosis was significantly better in the perioperative chemotherapy group.

At the 2012 ASCO annual meeting, overall survival data were reported, but no significant difference was seen between the two groups (ITT hazard ratio=0.88; p=0.339; eligible patients hazard ratio=0.87; p=0.303)[13)].

2.2.2. Clinical Studies for Unresectable or Difficult-to-Resect Liver Metastases from Colorectal Cancer

Several clinical studies have found that the liver became resectable during subsequent chemotherapy in patients who were deemed to have initially unresectable disease. (Table 7 (i), Table 7 (ii))

Table 7 (i). Results of Liver Resection in Clinical Studies for Advanced or Recurrent Colorectal Cancer

Study title	No. of patients (No. of patients with liver metastases)	Liver resection rate (% of patients with liver metastases)	R0 liver resection rate (% of patients with liver metastases)
**First-BEAT**[14] Oxaliplatin-based CT + Bmab	349	20.3%	15.4%
Irinotecan-based CT + Bmab	230	14.3%	11.7%
**NO16966**[15] FOLFOX/XELOX ± Bmab	417	Not Reported	17.1% vs. 12.6%
**CRYSTAL**[16] FOLFIRI ± Cmab	256	Not Reported	9.8% vs. 4.3%
**OPUS**[17] FOLFOX ± Cmab	337 (Total no. of patients)	6.5% vs. 3.6% (% of total no. of patients)	4.7% vs. 2.4% (% of total no. of patients)

Table 7 (ii). Results of Liver Resection in Clinical Studies for Liver Metastases from Colorectal Cancer that are Difficult to Curatively Resect

Study title	No. of patients	Response rate	Liver resection rate	R0 liver resection rate	Prognosis
**Alberts, et al.**[18] FOLFOX	42	60%	40%	33%	Median OS: 26 months
**BOXER**[19] XELOX + Bmab	45	78%	CR rate: 9% Liver resection rate: 36%	CR rate: 9% R0 liver resection rate: 20%	1-year PFS rate: 50% 1-year OS rate: 86%
**CELIM**[20] FOLFOX + Cmab	53	68%	51%	38%	Median PFS: 11.2 months - Median OS: 35.7 months
FOLFIRI + Cmab	53	57%	49%	30%	Median PFS: 10.5 months Median OS: 29.0 months
**POCHER**[21] FOLFOXIRI + Cmab	43	79%	—	60%	Median PFS: 14 months Median OS: 37 months

The definitions of "difficult to curatively resect at initial presentation" in clinical studies for liver-only metastases from colorectal cancer that were difficult to curatively resect at initial presentation are tabulated below (Table 8).

Table 8. Definitions of Curatively Unresectable at Initial Presentation for Liver Resection in Clinical Studies

Study	Definition
**Alberts, et al.**[18]	Multiple liver metastases in both hepatic lobes<br>Proximity of tumor to major vascular structures, preventing preservation of an adequate hepatic remnant<br>Large tumor jeopardizing remnant liver function
**BOXER**[19]	Five or more liver metastases<br>Liver metastasis diameter larger than 5 cm<br>Location and distribution of metastatic disease within the liver unsuitable for resection<br>Residual liver parenchyma volume not adequate for maintaining viable liver function<br>Unable to retain adequate vascular flow to maintain viable liver function<br>Synchronous liver metastases
**CELIM**[20]	Five or more liver metastases<br>For technical reasons, is concluded to be unresectable or difficult-to-resect<br>▪ Judged to be technically unresectable, in light of remaining hepatic function<br>▪ Invasion into all hepatic veins is evident<br>▪ Invasion into both right and left hepatic arteries or portal veins is evident
**POCHER**[21]	Five or more liver metastases<br>Diameter larger than 5 cm<br>Hilar metastasis, extrahepatic distant metastasis (except micronodular lung metastases)

2.2.3. Clinical Studies of Adjuvant Chemotherapy for Liver Metastases of Colorectal Cancer

In an investigation of adjuvant chemotherapy following liver resection, the FFCD ACHBTH AURC 9002 study compared two treatments after curative liver resection: 5-FU/LV for six months (n=86) versus surgery alone (n=85)[22]. The five-year disease-free survival rates in the 5-FU/LV group and the surgery-only group were 33.5% and 26.1% (odds ratio=0.66; p=0.028), and the five-year overall survival rates were 51.1% and 41.1% (odds ratio=0.73; p=0.13). A pooled analysis of the results of the French FFCD study and the English ENG study also showed that adjuvant 5-FU/LV is potentially more useful than resection alone (the median progression-free survival was 27.1 months versus 18.8 months, hazard ratio=1.32; p=0.058)[23].

A confirmatory study to compare FOLFIRI versus 5-FU/LV after curative liver resection failed to demonstrate that FOLFIRI was useful. In the field of molecular targeted drugs, a confirmatory study to compare XELOX plus bevacizumab versus XELOX was ended after 72 patients of the planned 500 were evaluated. The two-year disease-free survival rates for these 72 patients were 70% and 52% (p=0.072), respectively[24].

In Japan, the JCOG0603 study, which aims to compare FOLFOX with resection only after curative resection, is currently underway[25].

2.3. Rationale for the Study Plan

2.3.1. Treatment Regimen in this Study

While it was not a comparison of chemotherapy regimens in liver metastases from colorectal cancer, a study conducted by Tournigand et al. to compare the usefulness of FOLFIRI versus FOLFIRI followed by FOLFOX as second-line therapy in advanced or recurrent colorectal cancer after FOLFOX as first-line chemotherapy[26] showed that the rate of liver resection in the FOLFIRI-first group was 9%, versus 22% in the FOLFOX-first group (p=0.02). Other reported data show that neoadjuvant chemotherapy is associated with pathological changes of liver parenchyma[27], leading to concern about toxicity to remnant liver caused by neoadjuvant chemotherapy. There are two types of liver injury: the first involves vascular changes caused by oxaliplatin-based chemotherapy (sinusoidal dilatation with engorgement of red blood cells, associated with sinusoidal obstruction syndrome such as that seen in perisinusoidal fibrosis or venous obstruction; Rubbia-Brandt[28] Grades 2-3: 18.9%[27]), and the other is steatohepatitis caused by irinotecan-based chemotherapy (with severe steatosis, lobular inflammation, or hepatocyte ballooning; Kleiner [29] score ≧4; 20.2%[27]). The possibility that steatohepatitis due to irinotecan-based chemotherapy will increase the 90-day mortality rate (14.7%) is a cause for concern.

In combination with chemotherapy, the antiangiogenic drug bevacizumab protects against pathological changes of liver parenchyma caused by chemotherapy, and its pathological benefits suggest that it could potentially improve prognosis.

In a retrospective study of sinusoidal dilatation in 105 patients who underwent liver resection after 5-FU/oxaliplatin therapy with or without concomitant bevacizumab, Ribero et al.[30] showed that the incidence of Rubbia-Brandt Grade 2-3 sinusoidal dilatation was 27.9% in patients treated without bevacizumab, versus 8.1% in patients treated with bevacizumab (p=0.006). Recently, it has also been suggested that in addition to inhibiting the development of oxaliplatin-induced sinusoidal dilatation, and by inhibiting portal hypertension, bevacizumab could potentially inhibit splenomegaly (defined as a spleen volume increase of at least 30%: 32% with bevacizumab versus 48% without bevacizumab; p＝0.013) and thrombocytopenia (platelet count <150,000/μL; at 3 months, 24% with bevacizumab versus 43% without bevacizumab; p＝0.006; from 3 months onwards, p＝0.003)[31].

In a retrospective study of 305 patients who underwent liver resection after chemotherapy regimens with or without concomitant bevacizumab, Blazer et al.[32] sought to identify the independent pathologic response factors that influence prognosis following liver resection. In 81 patients receiving concomitant bevacizumab with oxaliplatin-based chemotherapy before liver resection, the pathologic response was 62.9% (complete response = 0% residual cancer cells: 8.6%; major response = 1% to 49% residual cancer cells: 54.3%), and in 50 patients receiving oxaliplatin-based chemotherapy alone, the pathologic response was 44.0% (complete response = 0% residual cancer cells: 12.0%; major response = 1% to 49% residual cancer cells: 32.0%), suggesting that combination therapy with bevacizumab improves the percentage of residual cancer cells, an independent predictor of prognosis.

Meanwhile, Rubbia-Brandt et al.[33] found that the tumor regression grade (TRG; Table 9) proposed by Mandard and coworkers[34] in esophageal cancer is a factor that affects prognosis after liver resection in metastasis to liver from colorectal cancer. Klinger et al.[35] reported that a major histological response (MjHR, TRG 1-2) was achieved in 38.0% of patients, a partial histological response (PHR, TRG 3) in 28.0% of patients, and a non histological response (NHR, TRG 4 and 5) in 34.0% of patients on FOLFOX/XELOX plus bevacizumab therapy, while MjHR (TRG 1-2) was achieved in 10.0%, PHR (TRG 3) in 24.0%, and NHR (TRG 4 and 5) in 66.0% of patients on FOLFOX/XELOX therapy, suggesting that concomitant treatment with bevacizumab improves TRG. TRG is an evaluation marker of fibrosis, but Li-Chang et al. reported that mTGR (Table 10), an indicator of both fibrosis and necrosis which includes a classification of infarct-like necrosis (necrosis associated with therapeutic response, as distinguished from usual necrosis) similar to that of fibrosis, has a stronger association with prognosis[36].

Because of its cytostatic mechanism of action, it has been suggested that bevacizumab cannot be adequately evaluated using the RECIST guidelines for evaluating objective tumor response to conventional cytotoxic drugs[37],

and in the present research, we will undertake a prospective investigation that includes the association with prognosis for these pathologic responses to bevacizumab.

Table 9. Tumor Regression Grade (TRG)[33]

Grade	Evaluation
TRG1	Tumor cells have disappeared, replaced by "fibrosis" (pCR)
	(absence of tumor cells replaced by abundant fibrosis)
TRG2	Scarce, scattered residual tumor cells only, mostly replaced by "fibrosis"
TRG3	Abundant "fibrosis" is seen, but residual tumor cells are still numerous
	(more residual tumor cells throughout a predominant fibrosis)
TRG4	Scant "fibrosis" is present, and residual tumor cells are still numerous
	(large amount of tumor cells predominating over fibrosis)
TRG5	No "fibrosis" seen, tumor cells predominate
	(most exclusively to tumor cells without fibrosis)

Table 10. mTRG (modified Tumor Regression Grade)[36]

Grade	Evaluation
mTRG1	Tumor cells have disappeared, replaced by "fibrosis/infarct-like necrosis" (pCR)
mTRG2	Scarce, scattered residual tumor cells only, mostly replaced by "fibrosis/infarct-like necrosis"
mTRG3	Scant "fibrosis/infarct-like necrosis" is present, and residual tumor cells are still numerous
mTRG4	Scant "fibrosis/infarct-like necrosis" is present, and residual tumor cells are still numerous
mTRG5	No "fibrosis/infarct-like necrosis" seen, tumor cells predominate

In coadministration with chemotherapy, the anti-EGFR antibody cetuximab improves the response rate and yields a excellent rate of curative liver resection, for which it has attracted attention.

Folprecht et al. reported the results of a randomized Phase II study of FOLFOX/FOLFIRI plus cetuximab in patients with liver-only metastases from colorectal cancer (with five or more metastatic nodules that are technically difficult to resect)[20]. Among 106 patients evaluated, the response rate was 68% and the R0 resection rate 38% in the 53 patients receiving FOLFOX plus cetuximab, and the response rate was 70% and the R0 resection rate was 33% in the 67 patients with *KRAS* wild-type status. As well, the response rate was 60%, the liver resection rate was 41%, and the R0 liver resection rate was 34% in the 60/106 patients in whom radiology review was possible.

For the 106 patients in total, the median progression-free survival was 10.8 months, the median overall survival was 33.1 months, and in the 33 patients with KRAS wild-type status who received FOLFOX plus cetuximab, the median progression-free survival was 12.1 months and the median overall survival was 35.8 months, and in the 36 patients who underwent R0 liver resection, the median progression-free survival was 15.4 months (the median recurrence-free survival after R0 liver resection was 9.9 months) and the median overall survival was 46.7 months.

Meanwhile, the compatibility of cetuximab with oxaliplatin-based chemotherapy obtained in a Phase III clinical study appears to vary with the regimen.

In a Phase III clinical study which compared OxMdG/XELOX with or without concomitant cetuximab[38], the median overall survival in *KRAS* wild-type disease was 17.9 months in the OxMdG/XELOX group (n=367), versus 17.0 months in the OxMdG/XELOX plus cetuximab group (n=362). There was no significant difference between the two groups with respect to median overall survival (hazard ratio=1.04; p=0.67). Additionally, the median progression-free survival was 8.6 months in the OxMdG/XELOX group (n=367) and 8.6 months in the OxMdG/XELOX plus cetuximab group (n=362). There was no significant difference between the two groups with respect to median progression-free survival (hazard ratio=0.96; p=0.60), and subgroup analysis by combination therapy regimen showed an interaction between OxMdG and XELOX (p=0.07), raising concerns about compatibility with XELOX.

The guidelines for the treatment of colorectal cancer have been indicated two points regarding corraration between efficacy of anti-EGFR antibody and level of EGFR gene expression.

1. The cetuximab have only been included in EGFR positive cases in almost clinical trials and have been endorced for EGFR positive mCRC by drug approval. On the other hand, although, panitumumab have been same situation in clinical trials, but it have been endorced for all EGFR status by drug approval.
2. Wierzbicki et al. reported the pahse II study of cetuximab efficacy in patients with pretreated EGFR negative metastatic colorectal cancer. Among 85 patients evaluated, overall response was 8.2% (7pts) and stable disesase was 41.2% (35pts)[59)]. Its data showed the non-inferiority of cetuximab efficacy for EGFR positive cases.

Based on two points, EGFR status is not needed for criteria in ATOM trial.

In summary, when contemplating the multidisciplinary treatment strategy for *KRAS* wild-type liver-limited, initially unresectable colorectal cancer, despite the presence of data suggesting that "liver resection rate", "improvement of response rate", and "pathologic improvement" improve prognosis, there is no unequivocal evidence that molecular targeted therapy in combination with chemotherapy is better with either anti-VEGF antibody products or anti-EGFR antibody products.

Therefore, in the present research, we planned a randomized Phase II clinical study to conduct an exploratory comparison of mFOLFOX6 plus bevacizumab versus mFOLFOX6 plus cetuximab in *KRAS* wild-type, difficult-to-resect, liver-only metastases from colorectal cancer.

Efficacy and safety in the two treatment groups will be compared via endpoints including progression-free survival, curative liver resection, and post-resection life expectancy.

2.3.2. Ongoing Treatment

No ongoing treatment is to be specified. However, information on the details of any ongoing treatment will be collected at follow-up. Ongoing treatment could conceivably entail crossing over opposing groups (Group B after Group A, or Group A after Group B). After the protocol surgery is performed and the adjuvant chemotherapy is discontinued or completed on schedule (discontinuation of protocol treatment), patients will be monitored without treatment until recurrence. After recurrence, the selection of the same post-recurrence treatment as the protocol treatment will not be prohibited, in which instance it will be concluded that the same therapy has been selected as ongoing treatment.

After the toxicity-related discontinuation criteria have been met and an assessment of "protocol treatment discontinuation" has been made because of patient refusal, it is not recommended that the same regimen as the protocol treatment be continued as "ongoing treatment". If "the discontinuation criteria are met, but the same treatment is to be continued at the discretion of the subinvestigator and the patient's wishes", the situation will be regarded as "protocol treatment continued after deviation from discontinuation criteria", rather than "protocol treatment discontinued → ongoing treatment". If the discontinuation criteria for the protocol treatment are met but it is deemed clinically appropriate to "continue the protocol treatment", the study secretariat should be consulted. With the agreement of the study secretariat and the subinvestigator, decide whether the situation should be regarded as "protocol treatment discontinued → ongoing treatment provided" or "deviation occurred and protocol treatment continued". If "deviation occurred and protocol treatment continued" occurs frequently, there is a possibility that the discontinuation criteria for protocol treatment are clinically inappropriate. In this situation, the study secretariat will consult with the study coordinating committee, and investigate whether to revise the discontinuation criteria for protocol treatment.

2.4. Study Design

2.4.1. Rationale for Endpoints

Evaluations of the relationships between overall survival and progression-free survival and response rate in advanced or recurrent colorectal cancer, and investigations of whether they can serve as surrogate markers for overall survival showed that progression-free survival is the most strongly correlated with overall survival, and that progression-free survival is appropriate as a surrogate marker for overall survival[39, 40]. Hence, progression-free survival, which allows usefulness to be evaluated sooner than overall survival, is designated as the primary endpoint in this study.

2.4.2. Clinical Hypothesis and Rationale for Sample Size

The main objective of this study is to select the more promising treatment method between mFOLFOX6 plus bevacizumab (Group A) and mFOLFOX6 plus cetuximab (Group B) in patients with liver-only metastases from unresectable, KRAS wild-type colorectal cancer.

Simple comparisons are difficult, but earlier research indicated that progression-free survival in *KRAS* wild-type advanced or recurrent colorectal cancer tended to be better with bevacizumab than cetuximab (Table 11), and it is therefore possible that mFOLFOX6 plus bevacizumab may be superior to mFOLFOX6 plus cetuximab. For the response rate in advanced or recurrent colorectal cancer, and for improvement in resection rate associated with improved response rate by contrast, it can be expected that cetuximab will be superior and prognosis improved in resected cases (Table 12). Therefore, in the planned study involving liver-only metastases, it is also possible that mFOLFOX6 plus cetuximab will be superior to mFOLFOX6 plus bevacizumab. Hence, it was decided to adopt a randomized, two-phase selection design for identifying the most promising treatment method.

Table 11. Median Progression-free Survival in *KRAS* Wild-type Advanced or Recurrent Colorectal Cancer (reports of Phase III studies)

Study title	Treatment	No. of patients	Median progression-free survival (*KRAS* wild type)
**AVF2107g**[41]	IFL + Bmab	85 patients	13.5 months
**CAIRO2**[42]	XELOX + Bmab	155 patients	10.6 months
**PACCE Ox-CT**[43]	Oxaliplatin-based chemotherapy + Bmab	203 patients	11.5 months
**PACCE Iri-CT**[43]	Irinotecan-based chemotherapy + Bmab	58 patients	12.5 months
**MACRO**[44]	XELOX + Bmab	216 patients	10.9 months
**CRYSTAL**[16]	FOLFIRI + Cmab	172 patients	9.9 months
**COIN**[38]	Oxaliplatin-based chemotherapy + Cmab	362 patients	8.6 months
**NORDIC VII**[45]	FLOX + Cmab	97 patients	7.9 months

Table 12. Improvements in the Response Rate and R0 Resection Rate Reported in Previous Phase III Clinical Studies in Advanced or Recurrent Colorectal Cancer (ITT)

Study title	Treatment	Response rate	Improvement in R0 resection rate (versus control group)
**NO16966**[15]	FOLFOX/XELOX + Bmab (n=699)	38%	8.4% vs. 6.1%
**CRYSTAL**[16]	FOLFIRI + Cmab (n=599)	46.9%	4.8% vs. 1.7%

In the main analysis, the hazard ratio for Group B versus Group A will be estimated for the primary endpoint, progression-free survival, based on a stratified proportional-hazard model using assignment adjustment factors other than study site. If the point estimate of the hazard ratio of Group B to Group A is less than 1.00, it will be concluded that Group B is the more promising treatment, and conversely, if the point estimate of the hazard ratio is 1.00 or higher, it will be concluded that Group A is the more promising treatment. However, it is also possible that summarization using hazard ratios is inappropriate, for example, when the shapes of the survival curves in each group are substantially different and do not intersect. In such cases, the question of which treatment is more promising will be decided by a comprehensive assessment, taking into account the toxicity of each. For reference purposes, estimates including the cumulative progression-free survival curves, median progression-free survival, and annual progression-free survival rate will be calculated using the Kaplan-Meier method.

The one-year progression-free survival rates for mFOLFOX6 plus bevacizumab and mFOLFOX6 plus cetuximab expected in *KRAS* wild-type disease are hypothesized to be 50%. This value was established with reference to the one-year progression-free survival rate in the XELOX plus bevacizumab group in the BOXER study and the one-year progression-free survival rate for FOLFOX plus cetuximab in *KRAS* wild-type disease in the CELIM study.

The selection design proposed by Liu et al. (Liu et al, Biometrics 49: 391–398, 1993) was used to calculate the number of patients needed to select the treatment group at a high probability if the true one-year progression-free survival rate in the better study group was to be 5% to 8% higher than in the other group. The probability of correctly selecting the treatment method at a 5% higher survival rate (50% to 55%) when 80 patients are assigned to each group (a total of 160 patients in two groups) is 80.7%, and the probability of correct selection at an 8% higher rate (50% to 58%) is 90.0% (however, this assumes that both groups follow an exponential distribution, an enrollment period of two years, a follow-up period of two years, and a uniform accumulation rate. Calculations are based on SAS v.9.3 and an in-house program prepared using MATLAB v.2011b). The selection design will require a relatively large-scale study (for example, see Lee et al, J Clin Oncol 23: 4450-5, 2005), and we plan to accumulate this number of patients for selection with a high probability of detecting a clinically meaningful difference. Including an allowance for censoring and the likelihood of patients who may be found ineligible after enrollment, the planned number of patients was set at 90 per group, for a total of 120 patients in the two groups combined.

2.4.3. Rationale for Selection of Assignment Adjustment Factors

To determine prognostic/predictive factors for colorectal cancer, Köhne et al., analyzed prognosis for 3,825 subjects (2,549 subjects for learning and 1,276 for validation) in previously-reported clinical studies of treatment primarily with 5-FU for metastatic colorectal cancer (19 Phase III studies and three Phase II studies), and found that the tumor parameters responsible for poor prognosis were number of metastases - more than one or more than two - and the presence of liver metastases or peritoneal carcinomatosis (metastasis), and among the clinical parameters, ECOG PS 0 or 1 was a positive prognostic factor. Ultimately, they classified patients into three groups: low risk (PS 0 or 1; number of metastatic sites: 1; n=1, 111), intermediate risk (PS 0 or 1; number of metastatic sites: 2 or more; and ALP <300 U/l, or PS 2 or higher; number of metastatic sites: 1; WBC <10×10^9/l; n=904), and high risk (PS 0 or 1; number of metastatic sites: 2 or more; and ALP >300 U/l, or PS 2 or higher; number of metastatic sites: 2 or more, or PS 2 or higher and WBC >10×10^9/l; n=534), and reported that overall survival was 15 months in the low-risk group, 10.7 months in the intermediate-risk group, and 6.1 months in the high-risk group (P value for three risk groups: P <0.0001)[46). Following this, the data for combination bevacizumab plus chemotherapy in a Phase II/III clinical study reported by Kabbinavar et al. were analyzed for three risk groups, and similar results were reported for overall survival and progression-free survival[47). Because the subjects in the present study are required to have only one organ with metastases, the liver, and a PS of 0 or 1 only, adjustment factors other than the Köhne index were selected, as discussed below.

In an investigation into prognosis for synchronous and metachronous liver metastases in 155 patients, Tsai et al. reported that the five-year survival rate was 34.2% in patients with synchronous liver metastasis and 54.6% in metachronous liver metastasis, finding that the prognosis in synchronous liver metastasis was significantly worse when measured by the disease-free survival period (p=0.004)[48). It has also been reported that in advanced colorectal cancer with synchronous metastasis, there is a difference in prognosis between chemotherapy following primary resection and chemotherapy without prior primary resection. In the FFCD-9601 study reported by Ferrand et al. in 216 patients with synchronous metastases from colorectal cancer, the progression-free survival period in patients with or without primary resection before chemotherapy was 5.1 months in the resection group (n=156) versus 2.9 months (p=0.0002) in the non-resection group (n=60), and overall survival was 16.3 months in the former and 9.6 months (p<0.0001) in the latter group. According to Venderbosch et al., similar results were reported for both the CAIRO study and CAIRO2 study[50).

In research in 36 patients who underwent curative resection of liver metastases from among 106 patients with

unresectable liver-only metastases from colorectal cancer who received FOLFOX/FOLFIRI plus cetuximab, Folprecht et al. reported that the disease-free survival period by number of metastases was as follows: <5 (n=11): 16.8 months; 5-10 (n=22): 8.2 months; 11 or more (n=3): 2.5 months[20]. The Japanese Society of Hepato-Biliary-Pancreatic Surgery analyzed data for 727 patients who underwent curative liver resection at 11 sites between 2000 and 2004, to identify the independent prognostic factors that affect disease-free survival after liver resection, and scored each factor to create a nomogram. They identified the following six factors: synchronous metastases, primary lymph node metastasis, number of metastases, largest tumor diameter, extrahepatic metastasis, and CA19-9 level. Weighting these factors using the nomogram yielded an estimated median disease-free survival of 8.4 y for a score of zero, 1.9 y for a score of 5, 1.0 y for a score of 10, and 0.6 y for a score above 10[51]. Data have also been published on the five-year recurrence-free survival rate and five-year overall survival rate by number of metastases for 736 patients who underwent curative liver resection at the University of Tokyo and the Cancer Institute Ariake Hospital between 1993 and 2008. For fewer than four metastatic tumors (n=493), the rates were 29% and 51%, for four to seven tumors (n=141), the rates were 12% and 41%, and for eight or more tumors, the rates were 1.7% and 33%. Multivariate analysis showed that the following factors independently predicted outcome: primary lymph node metastasis, extrahepatic distant organ metastasis, maximum liver metastasis size greater than 5 cm, and tumor exposure during resection[52].

From these results, the following were selected as adjustment factors for the assignment process, representing factors that could conceivably affect the primary endpoint, progression-free survival: (i) synchronous liver metastases, primary lesion present/synchronous liver metastases, primary lesion absent/metachronous liver metastases; (ii) number of metastases (1-4/more than 5); (iii) maximum diameter of metastatic lesions (smaller than 5 cm/5 cm or bigger); and (iv) oxaliplatin use in adjuvant chemotherapy - yes/no.

Adjustment factors

(1) Synchronous liver metastases, primary lesion present/synchronous liver metastases, primary lesion absent/metachronous liver metastases
(2) Number of metastases (1-4/more than 5)
(3) Maximum diameter of metastatic lesions (smaller than 5 cm/5 cm or bigger)
(4) Oxaliplatin use in adjuvant chemotherapy - yes/no

2.5. Summary of Benefits and Disadvantages to be Expected from Study Participation

2.5.1. Expected Benefits

The drugs to be used in this study have approved indications under the health insurance scheme, and and the treatment methods are covered under health insurance. The drug costs and other treatment-related expenditure during the study period for patients participating in the study will be covered by the patient's health insurance or paid out of their own pockets; therefore, compared with routine treatment, there are no particular therapeutic or economic benefits to be gained by participating in this study.

2.5.2. Expected Disadvantages

It is expected that the adverse events described in section 7. "Expected Adverse Events" may occur, and depending on circumstances, it is also possible that treatment-related death might result. However, such events can also similarly occur in routine clinical practice, and will not be affected by participation in this study. Conversely, by establishing the selection criteria (see section 4) and the treatment plan and criteria for amendment of treatment (see section 6) and reinforcing their comprehension among members of the study organization, and by monitoring more frequently than in routine clinical practice, it may be possible to reduce the risk of adverse events and prevent adverse events from becoming more serious.

2.6. Significance of this Research

There is currently insufficient evidence to guide the selection of either mFOLFOX6 plus bevacizumab or mFOLFOX6 plus cetuximab for *KRAS* wild-type liver-only metastases from colorectal cancer. This study may yield valuable evidence for deciding on treatment options in this patient population.

As well, the true endpoint for the treatment of liver-only metastases from colorectal cancer lies in improving life expectancy through curative liver resection. In previous clinical studies, the evaluation of treatment for liver-only metastases from colorectal cancer has tended to use the rate of curative liver resection as the primary endpoint, but this study will be conducted with progression-free survival as the primary endpoint. Via this aspect too, the study may yield valuable evidence for deciding on treatment options in patients with liver-only metastases from colorectal cancer.

2.7. Supplementary Research

We will also conduct an exploratory investigation into the possibility of predicting the therapeutic effect of bevacizumab by "measurement of plasma pVEGF-A and other angiogenic factors".

This supplementary research will be described more thoroughly in a separate protocol.

This study plan does not include an investigation into long-term prognosis (3, 5, and 10-year survival rates, recurrence-free rates, etc.) in the applicable patient population, for reasons associated with the duration of the study. The researchers will separately plan an investigation into this question (the authority and responsibility for the surveillance research in this case lies with the principal investigator).

2.8. Drug Information

The drugs used in this research are as follows. The most recent prescribing information for each drug should be used to inform the details and handling of each.

2.8.1. Bevacizumab: Avastin for IV Infusion 100 mg/4 ml, 400 mg/16 ml

1. Mechanism of action and characteristics

   Bevacizumab is a recombinant humanized monoclonal antibody that binds to human vascular endothelial growth factor (VEGF), and by specifically binding to human VEGF, inhibits the binding of VEGF to VEGF receptors expressed on vascular endothelial cells. By inhibiting the biological activity of VEGF, bevacizumab inhibits tumor proliferation by suppressing angiogenesis in tumor tissue. Bevacizumab also reduces VEGF-enhanced vascular permeability and thereby reduces elevated interstitial pressure in tumor tissue.

2. Indications

   Unresectable advanced or recurrent colorectal cancer

3. Main adverse drug reactions (Japanese studies)

   1) Bevacizumab plus FOLFOX4 therapy (n=14)

      Overall, there were 207 adverse drug reactions in 14 patients (100%), and the main adverse drug reactions included neutrophil count decreased in 12 subjects (85.7%), white blood cell count decreased in 11 patients (78.6%), nausea in 11 patients (78.6%), anorexia in 11 patients (78.6%), and neurotoxicity in 10 patients (71.4%). (At approval)

   2) Bevacizumab plus 5-FU/I-LV therapy

      Overall, 186 adverse drug reactions were reported in 17 patients (94.4%). The main adverse drug reactions included nausea in 8 patients (44.4%), epistaxis in 8 patients (44.4%), diarrhea in 7 patients (38.9%), hypertension in 6 patients (33.3%), vomiting in 6 patients (33.3%), and stomatitis in 6 patients (33.3%).

4. Clinically significant adverse reactions

   Shock, anaphylactoid symptoms, infusion reaction (urticaria, dyspnea, lip edema, pharyngeal edema, etc.), gastrointestinal perforation, delayed wound healing, hemorrhage (tumor-associated hemorrhage, including that from the metastasis site (gastrointestinal tract hemorrhage (hematemesis, melena), pulmonary hemorrhage (hemoptysis), cerebral hemorrhage) and mucosal hemorrhage (epistaxis, gingival bleeding, vaginal bleeding)), thromboembolism (cerebrovascular accident, transient ischemic attack, myocardial infarction, angina pectoris, cerebral ischemia, cerebral infarction and other arterial thromboembolisms, and deep vein thrombosis, pulmonary embolism, and other venous thromboembolisms), hypertensive encephalopathy, hypertensive crisis

5. Contraindications

   History of hypersensitivity to any of the components of bevacizumab

6. Main drug interactions

   Contraindications for coadministration: None; Precautions for coadministration: None

2.8.2. Cetuximab: Erbitux Injection 100 mg

1. Mechanism of action and characteristics

   Cetuximab is a chimeric monoclonal antibody composed of a human IgG constant region and a murine antibody variable region, which binds with a high affinity for EGFR on EGFR-expressing cells.

   In various EGFR-expressing cancer cell lines, the in vitro proliferation-inhibiting activity of cetuximab was concentration-dependent. Additionally, the proliferation-inhibiting activity of cetuximab was verified in in vivo models using various EGFR-positive cancer cell lines (the human colorectal cancer cell line GEO and others).

2. Indications

   EGFR-positive unresectable advanced or recurrent colorectal cancer

3. Main adverse drug reactions (Japanese studies)

   Among 39 patients in the safety analysis set in a Japanese Phase II study in combination therapy with irinotecan hydrochloride in patients with EGFR-expressing colorectal cancer, the main adverse drug reactions were acne (87.2%), rash (61.5%), anorexia (56.4%), dry skin (51.3%), paronychia (51.3%), diarrhea (51.3%), stomatitis (51.3%), hypomagnesemia (51.3%), pruritus (43.6%), nausea (43.6%), fatigue (43.6%), and lymphocyte count decreased (30.8%).

4. Clinically significant adverse reactions

   Severe infusion reaction, severe skin symptoms, interstitial lung disease, cardiac failure, severe diarrhea

5. Contraindications

   Patients with a history of hypersensitivity to the components of this product

6. Main drug interactions

   Contraindications for coadministration: None; Precautions for coadministration: None

2.8.3. Oxaliplatin: Elplat IV Infusion Solution 50 mg, 100 mg

1. Mechanism of action and characteristics

   Platinum compound, a type of platinum-based pharmaceutical product. Oxaliplatin forms biological complexes (dichloro-1,2-diaminocyclohexane (DACH) platinum, mono-aquo-monochloro DACH platinum, diaquo DACH platinum) in the body, and by covalently binding to DNA in cancer cells, forms intra and inter-strand platinum-DNA cross-links. These cross-links interfere with DNA replication and transcription. Cell division is understood to be blocked by microtubule inhibition.

2. Indications

   Unresectable advanced or recurrent colorectal cancer

   Adjuvant chemotherapy in colon cancer

3. Main adverse reactions

   (In FOLFOX4 therapy, a total of 618 patients in a Phase II study outside Japan, and in monotherapy in Japan, Phase I and Phase II studies, and in combination therapy in Japan, a Phase I/II study)

   Hematologic toxicities: Leukopenia, neutropenia, hemoglobin decreased (anemia), thrombocytopenia; Gastrointestinal toxicities: diarrhea, nausea, vomiting, anorexia, stomatitis, constipation, abdominal pain; Liver: AST (GOT increased, ALT GPT) increased, total bilirubin increased; Psychoneurological: peripheral neurological symptoms; Other: fatigue, cough, alopecia

4. Clinically significant adverse reactions

   Peripheral neurological symptoms, shock or anaphylactoid symptoms, interstitial pneumonia or pulmonary fibrosis, myelosuppression, hemolytic uremic syndrome, visual field defect, visual field disorder, optic neuritis, or visual acuity reduced, thromboembolism, ventricular arrhythmia, myocardial infarction, hepatic vein occlusion, renal failure acute

5. Contraindications

   1) Oxaliplatin is contraindicated in patients with severe sensory abnormality or dysesthesia with functional impairment
   2) Patients with a history of hypersensitivity to any of the components of oxaliplatin or to other platinum-containing drugs
   3) Women who are pregnant and women of childbearing potential

6. Main drug interactions

   1) Contraindications in coadministration

      None

2) Precautions in coadministration:

Because it may enhance other therapies such as antineoplastic drugs, radiotherapy, or myelosuppression, the patient's symptoms should be monitored closely when given in coadministration, and if necessary, the dose reduced or the dosing interval increased. (Because its cytotoxic action is enhanced in coadministration.)

2.8.4. Fluorouracil: 5-FU Injection 250 Kyowa, etc., 250 mg

1. Mechanism of action and characteristics

The basic mechanism of the antitumor effect of 5-FU is thought to be the inhibition of DNA synthesis. 5-FU taken into tumor cells is metabolized to F-deoxy UMP through the same pathway as that for uracil. F-deoxy UMP antagonizes deoxy UMP in the thymidylate synthetase reaction, inhibiting the synthesis of thymidylate and in turn that of DNA. Meanwhile, 5-FU, like uracil, is known to be incorporated into RNA, forming F-RNA and inhibiting the formation of ribosomal RNA. These properties are also believed to contribute to the antitumor activity of this drug.

2. Indications

Remission of subjective and objective symptoms in the following diseases: Gastric cancer, colorectal cancer, breast cancer, pancreatic cancer, uterine cervical cancer, uterine body cancer, and ovarian cancer (for the following diseases however, 5-FU must be coadministered with another antineoplastic agent or radiation: esophageal cancer, lung cancer, head or neck cancer)

Combination therapy with other antineoplastic agents for the following malignancies: head and neck cancer

Combination therapy comprising levofolinate-fluorouracil IV infusion: colorectal cancer

3. Main Adverse Reactions

Data collected on the frequencies of adverse drug reactions reported at approval and up to February 1970 shows that among 1,936 patients, the main adverse drug reactions included 295 cases (15.2%) of anorexia, 239 cases (12.3%) of diarrhea or loose stools, 172 cases (8.9%) of malaise, 159 cases (8.2%) of nausea or vomiting, 153 cases (7.9%) of leukopenia, 129 cases (6.7%) of stomatitis, 92 cases (4.8%) of pigmentation, and 74 cases (3.8%) of alopecia.

4. Clinically significant adverse reactions

Dehydration symptoms, hemorrhagic enteritis, ischemic enteritis, necrotizing enteritis, and other serious forms of enteritis, pancytopenia, leukopenia, neutropenia, anemia, thrombocytopenia, and other forms of myelosuppression, shock or anaphylactoid symptoms, leukoencephalopathy, congestive heart failure, myocardial infarction, rest angina, acute renal failure, interstitial pneumonia, impaired liver function, jaundice, hepatic failure, gastrointestinal ulcer, severe stomatitis, acute pancreatitis, hyperammonemia with consciousness disturbance, hepatobiiary disorder (cholecystitis, bile duct necrosis, liver parenchymal injury, etc.), hand-foot syndrome, olfactory disturbance, loss of taste, fulminant hepatitis and other severe liver disorders, liver cirrhosis, ventricular tachycardia, nephrotic syndrome, oculomucocutaneous syndrome (Stevens-Johnson syndrome), toxic epidermal necrolysis (Lyell syndrome), hemolytic anemia

Phenytoin (brand names: Aleviatin, Hydantol, Phenytoin Powder, Phenytoin N): dyslalia, ataxia, disturbance of consciousness, and other forms of phenytoin intoxication may develop. (The mechanism is unknown, but fluorouracil may increase the blood concentration of phenytoin.)

Warfarin potassium: Because fluorouracil may enhance the activity of warfarin potassium, changes in coagulation function should be carefully monitored. (The mechanism is unknown.)

Other chemotherapies and radiotherapy: Because blood disorders, gastrointestinal tract disorders, and other adverse drug reactions may be enhanced, patient condition must be carefully monitored. If any abnormal findings occur, appropriate measures should be taken, such as reducing the dose or discontinuing the drug. (Adverse drug reactions may be mutually enhanced.)

5. Contraindications
   1) Patients with a history of serious hypersensitivity to the components of this product
   2) Receiving treatment with tegafur-gimeracil-oteracil potassium or with less than 7 days after discontinuation of such treatment
6. Main drug interactions
   1) Contraindications in coadministration
      Tegafur-gimeracil-oteracil (brand name: TS-1)
   2) Precautions in coadministration:
      Phenytoin, warfarin potassium, other chemotherapies or radiotherapy

2.8.5. Levofolinate calcium: Isovorin Inj. 25 mg, 100 mg

1. Mechanism of action and characteristics

   Activated folic acid preparation. Levofolinate enhances the antitumor effect of fluorouracil through a biological modulation process. Fluorodeoxyuridine monophosphate (FdUMP), the active metabolite of fluorouracil, binds to and inhibits the activity of thymidylate synthase (TS), thereby blocking thymidylate synthesis and in turn DNA synthesis. Levofolinate is converted to 5, 10-methylenetetrahydrofolate (5,10-CH2-THF) by a reduction process in cells. This 5,10-CH2-THF forms a ternary complex with FdUMP and TS, enhancing the antitumor effect of fluorouracil by delaying the dissociation of TS.
2. Indications
   1) Levofolinate-fluorouracil therapy
      Enhancement of the antitumor effect of fluorouracil in gastric cancer (unresectable or recurrent) and colorectal cancer
   2) Combination therapy with levofolinate-fluorouracil IV infusion
      Enhancement of the antitumor effect of fluorouracil in colorectal cancer
3. Main adverse reactions

   Among 336 patients for whom data on adverse drug reactions in treatment with levofolinate plus fluorouracil was collected, adverse drug reactions were reported in 297 patients (88.4%). The main adverse drug reactions were diarrhea, reported in 160 patients (47.6%), anorexia in 160 patients (47.6%), nausea and vomiting in 155 patients (46.1%), stomatitis in 69 patients (20.5%), and pyrexia in 64 patients (19.0%). The Grade 3 or higher adverse drug reactions were diarrhea, reported in 47 patients (14.0%), anorexia in 45 patients (13.4%), nausea and vomiting in 27 patients (8.0%), pyrexia in 5 patients (1.5%), and stomatitis in 3 patients (0.9%). The main changes in laboratory test values were white blood cell count decreased in 200 (60.7%) of 336 patients, hemoglobin decreased in 136 (40.5%) of 336 patients, total protein decreased in 48 (14.5%) of 332 patients, and platelet count decreased in 46 (13.7%) of 336 patients. The laboratory test parameters with Grade 3 or higher abnormal values were white blood cell count decreased, reported in 59 patients (17.6%), hemoglobin decreased in 30 patients (8.9%), and platelet count decreased in 8 patients (2.4%).
4. Clinically significant adverse reactions

   Severe diarrhea, serious enteritis, myelosuppression, shock or anaphylactoid symptoms, leukoencephalopathy or psychoneurological disorders, cardiac failure congestive, myocardial infarction, or rest angina, impaired hepatic function or jaundice, renal failure acute, interstitial pneumonia, gastrointestinal tract ulcer, serious stomatitis, hand-foot syndrome, disseminated intravascular coagulation (DIC), loss of smell, hyperammonemia, acute pancreatitis, fulminant hepatitis, liver cirrhosis, ventricular tachycardia, nephrotic syndrome, oculomucocutaneous syndrome (Stevens-Johnson syndrome), toxic epidermal necrolysis (Lyell syndrome), and hemolytic anemia
5. Contraindications

   Serious myelosuppression, diarrhea, comorbid serious infection, copious amounts of ascites or pleural

effusion, existing or previous serious cardiac disease, poor general condition, history of serious hypersensitivity to fluorouracil or the components of bevacizumab, currently undergoing treatment with tegafur/gimeracil/oteracil potassium or less than 7 days after discontinuation of such treatment

6. Main drug interactions

   1) Contraindications in coadministration

      Tegafur/gimeracil/oteracil potassium (brand name: TS-1): Because there is a risk of early serious hematology disorder, or diarrhea, stomatitis or other gastrointestinal disorders, this product should not be used in patients currently receiving treatment with tegafur/gimeracil/oteracil potassium or those for whom at least 7 days has not elapsed since discontinuing such treatment. (Gimeracil inhibits the catabolism of fluorouracil, markedly increasing the blood concentration of fluorouracil.)

   2) Precautions in coadministration:

      Phenytoin (brand names: Aleviatin, Hydantol, Phenytoin Powder, Phenytoin N): dyslalia, ataxia, disturbance of consciousness, and other forms of phenytoin intoxication may develop. (The mechanism is unknown, but fluorouracil may increase the blood concentration of phenytoin.)

      Warfarin potassium: Because fluorouracil may enhance the activity of warfarin potassium, changes in coagulation function should be carefully monitored. (The mechanism is unknown.)

      Other chemotherapies and radiotherapy: Because blood disorders, gastrointestinal tract disorders, and other adverse drug reactions may be enhanced, patient condition must be carefully monitored. If any abnormal findings occur, appropriate measures should be taken, such as reducing the dose or discontinuing the drug. (Adverse drug reactions may be mutually enhanced.)

3. Criteria and Definitions Used in this Study

3.1. Type

The colon is divided into the following 8 regions.

C: Cecum

[Attachment] Appendix (V)

A: Ascending colon

T: Transverse colon

D: Descending colon

S: Sigmoid colon

RS: Rectosigmoid

R: Rectum

Ra: Rectum above the peritoneal reflection

Rb: Rectum below the peritoneal reflection

P: Proctos

[Attachment] External skin (E)

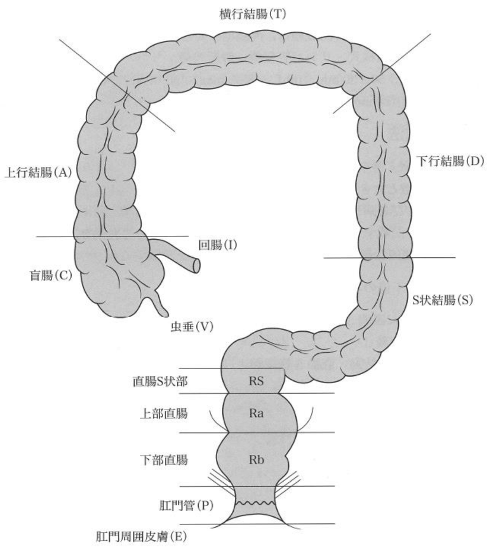

3.2. Staging criteria

Staging will be performed using the Japanese Classification of Colorectal Cancer (7th Edition) (January 2009, Japanese Society for Cancer of the Colon and Rectum).

The staging classification will determine the degree of progression corresponding to the following categories, and the highest of those is designated as the stage of the cancer.

<table>
<tr><th rowspan="2"></th><th colspan="3">H0, M0, P0</th><th>H1, H2, H3, M1,
P1, P2, P3</th></tr>
<tr><th>N0</th><th>N1</th><th>N2, N3</th><th>M1 (lymph nodes)</th></tr>
<tr><td>M</td><td>0</td><td colspan="3"></td></tr>
<tr><td>SM
MP</td><td>I</td><td rowspan="2">Ⅲa</td><td rowspan="2">Ⅲb</td><td rowspan="2">Ⅳ</td></tr>
<tr><td>SS, A
SE
SI, AI</td><td>II</td></tr>
</table>

3.3. Depth of Invasion

1. Depth of invasion

   M: Tumor has invaded the mucosa but not the submucosa

   SM: Tumor has invaded the submucosa but not the muscularis propria

   MP: Tumor has invaded the muscularis propria but no further

2. Sites with serosal layers

   SS: Tumor has invaded beyond the muscularis propria, but not into the serosal surface

   SE: Tumor invasion is observed on the serosal surface

   SI: Tumor has invaded adjacent organs

3. Sites without serosal layers

   A: Tumor has invaded beyond the muscularis propria

   AI: Tumor has invaded adjacent organs

3.4. Lymph Node Metastasis

NX: Degree of lymph node metastasis unknown

N0: No evidence of lymph node metastasis

N1: Three or fewer metastases to para-intestinal lymph nodes and intermediate lymph nodes

N2: Four or more metastases to para-intestinal lymph nodes and intermediate lymph nodes

N3: Metastases seen to main lymph nodes or lateral lymph nodes

3.5. Peritoneal Metastasis

PX: Peritoneal metastasis cannot be assessed

P0: No peritoneal metastasis

P1: Metastases only to adjacent peritoneum

P2: A few metastases to distant peritoneum

P3: Numerous metastases to distant peritoneum

3.6. Hepatic Metastasis

HX: Hepatic metastasis cannot be assessed.

H0: No hepatic metastasis

H1: One to four metastatic tumors, all of which are 5 cm or less in maximum diameter

H2: Other than H1 or H3.

H3: Five or more metastatic tumors at least one of which is more than 5 cm in maximum diameter.

3.7. Distant Metastasis to Extrahepatic Organs

MX: Distant metastasis cannot be assessed

M0: No distant metastasis

M1. Distant metastasis

3.8. Grade of Hepatic Metastasis

<table>
<tr><th></th><th>H1</th><th>H2</th><th>H3</th></tr>
<tr><td>N0
N1</td><td>A</td><td>B</td><td rowspan="3"></td></tr>
<tr><td>N2</td><td>B</td><td rowspan="2">C</td></tr>
<tr><td>N3
M1</td><td></td></tr>
</table>

3.9. Synchronous Liver Metastases and Metachronous Liver Metastases

Synchronous and metachronous liver metastases will be defined in this study as follows.

Synchronous liver metastases: Liver metastases present at the same time as the primary lesion, or liver metastases diagnosed within 24 weeks (six months) from the day of surgery for the primary lesion.

Metachronous liver metastases: Liver metastases that were not diagnosed at 24 weeks (six months) from the day of surgery for the primary lesion, but were diagnosed subsequently.

3.10. Evaluation of Residual Tumor After Resection

RX: Residual tumor cannot be assessed

R0: No residual tumor

R1: Tumor was removed, but cancer is exposed on the stump of the resected specimen or on the surface of the cut end

R2: Macroscopic residual tumor

The final decision on whether a protocol liver resection was a two-stage or multiple-surgery procedure for "evaluation of residual tumor" will be ultimately made by the study coordinating committee.

3.11. Evaluation of Tumor Regression Grade (TRG)[33)]

Grade	Evaluation
TRG1	Tumor cells have disappeared, replaced by fibrosis (pCR)
	(absence of tumor cells replaced by abundant fibrosis)
TRG2	Minimal residual tumor cells only, mostly replaced by fibrosis
	(rare residual tumor cells scattered throughout abundant fibrosis)
TRG3	Abundant fibrosis is seen, but residual tumor cells are still numerous
	(more residual tumor cells throughout a predominant fibrosis)
TRG4	Minimal fibrosis, but numerous residual tumor cells are still remaining
	(large amount of tumor cells predominating over fibrosis)
TRG5	No fibrosis, tumor cells predominate
	(most exclusively to tumor cells without fibrosis)

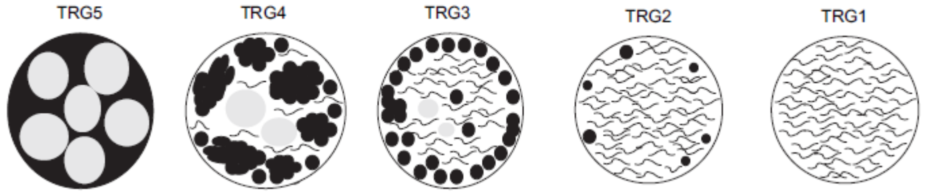

3.12. Evaluation of Modified Tumor Regression Grade (TRG)[36)]

Necrosis is defined as follows, and infarct-like necrosis (ILN) is evaluated as fibrosis.

	Evaluation
UN (usual necrosis)	Contains nuclear debris in a patchy distribution with necrosis and viable tumor cells mixed together (There is a rapid transition from viable cells to dying cells with pyknotic nuclei, or to anucleate cytoplasmic fragments.)
	containing nuclear debris in a patchy distribution, with the necrosis admixed and bordered by viable cells (There was a rapid transition from viable cell to dying cells with pyknotic nuclei, to anucleate cytoplasmic outlines.)
ILN (infarct-like necrosis)	Composed mostly of eosinophilic cytoplasmic remnants Located centrally within a lesion with absent or minimal nuclear debris. (Instead of viable tumor cells, the necrotic tissue is immediately surrounded by a layer of hyaline-like fibrosis with foamy macrophages.)
	being composed of large confluent areas of eosinophilic cytoplasmic remnants located centrally within a lesion with absent or minimal admixed nuclear debris. (Instead of viable tumor cells, the necrotic tissue was immediately surrounded by a layer of hyaline-like fibrosis with foamy macrophages.

The grading is in accordance with that for TRG.

Grade	Evaluation
mTRG1	Tumor cells have disappeared, replaced by "fibrosis/infarct-like necrosis" (pCR)
mTRG2	Rare residual tumor cells are scattered throughout only, replaced mainly by "fibrosis/infarct-like necrosis"
mTRG3	Adequate "fibrosis/infarct-like necrosis" is seen, but numerous residual tumor cells are still present
mTRG4	Minimal amount of "fibrosis/infarct-like necrosis" is seen, but numerous tumor cells still persist
mTRG5	No "fibrosis/infarct-like necrosis" is seen, and tumor cells account for most

3.13. Evaluation of Dangerous Halo (HALO)[53)]

Grade	Evaluation
Absent	None
Rare	Scattered tumor cells infiltrating the liver tissue for less than 10% of the circumference of the metastasis
Focal	Scattered cells infiltrating the liver tissue for less than 50% of the circumference of the metastasis
Diffuse	Scattered cells infiltrating the liver tissue for more than 50% of the circumference of the metastasis

3.14. Evaluation of Sinusoidal Obstruction Syndrome (SOS) (Rubbia-Brandt grade)[28)]

Grade	Evaluation
Grade 1	Mild (centrilobular sinusoidal dilatation limited to one-third of the lobular surface)
Grade 2	Moderate (centrilobular sinusoidal dilatation extending to two-thirds of the lobular surface);
Grade 3	Severe (complete lobular involvement of sinusoidal dilatation)

3.15. ECOG Performance Status

PS	Details
0	Fully active.<br>Able to carry on all pre-disease performance without restriction.
1	Restricted in physically strenuous activity but ambulatory and able to carry out work of a light or sedentary nature. e.g. Light house work, office work
2	Ambulatory and capable of all selfcare, but unable to carry out any work activities.<br>Up and about more than 50% of waking hours.
3	Capable of only limited selfcare.<br>Confined to bed or chair more than 50% of waking hours.
4	Completely disabled. Cannot carry on any selfcare.<br>Totally confined to bed or chair.

4. Inclusion Criteria

4.1. Eligibility Criteria

Patients who meet all of the following criteria will be eligible for enrollment in the study. Both men and women may be enrolled.

1. Histopathologically confirmed colorectal cancer (adenocarcinoma). Appendix cancer and anal cancer are excluded.
2. *KRAS* wild type[NB)].
   NB: RAS test for wild-type status should confirm the presence of mutations in at least KRAS Codons 12 and 13 (Exon 2). RAS test (except for KRAS Codons 12 and 13 (Exon 2)) is not considered for registration. In case of assessment for KRAS Codons 59, 61 and 146 (Exon 3 and 4) and NRAS Exon 2, 3, 4 and etc, all status are confirmed to be wild type.
3. Synchronous or metachronous liver-only metastasis, with no history of or coexisting extrahepatic distant metastasis or recurrence.
   However, for cases of synchronous liver metastasis with the primary lesion present at enrollment, the primary lesion must be no more than two-thirds of the circumference and be resectable.
   If the primary lesion is greater than two-thirds of the circumference, the patient can be enrolled after resection.
   NB: If the endscpe can not be passed to the primary tumor, enrollment should follow primary resection or creation of colostomy. In this case, patients will be regisotred after 14 days from finishing surgery.
   NB: If the endscope cac be passed to permary tumor, patients will be registered without primary tumor resection. If the primary tumor resection will be performed, patients will be regisotred after 14 days from finishing surgery.
   NB: In case patient who had intestinal obstruction and performed colostmy in synchronous liver limited mCRC at the time of registration can be enrolled after 14 days from finishing surgery. (in case of stent placement, patient can not be enrolled)
   NB: In case patient who had intestinal obstruction and performed colostmy in synchronous liver limited mCRC at the time of registration can be enrolled. (in case of stent placement, patient can not be enrolled)
   NB: Superior mesenteric lymph node metastases (#214) and periaortic lymph node metastases (#216) are not included in the primary regional lymph nodes. Patients with positive hepatic hilar lymph node metastases are not included in those with liver-only metastases.
   NB: Synchronous multiple colorectal cancers that fall within the scope specified in the protocol can be enrolled (curative resection possible)
   NB: Defined specifically in this study
4. Patients with at least one measurable lesion (RECIST Ver. 1.1) with long axis length 1 cm or more on diagnostic imaging (contrast-enhanced CT or contrast-enhanced liver MRI) that can be continuously evaluated in accordance with the protocol requirements, who meet any of the following criteria[NB)].
   1) Five or more liver metastases.
   2) Liver metastasis with maximum diameter exceeding 5 cm.
   3) Technically unresectable, in light of remaining hepatic function.
   4) Invasion into all hepatic veins or inferior vena cava.
   5) Invasion into both right and left hepatic arteries or portal veins.

   NB: Standards for difficult-to-resect liver metastases from colorectal cancer defined by the Protocol Preparation Committee in this study.
   NB: The diagnostic imaging method which is assessed for liver limited metastasis should be contrast CT (chest, abdomen, pelvis). If contrast CT will not be arranged due to iodine contrast media allergy, non-contrast CT (abdominal) + MRI (liver) should be arranged and the same diagnostic imaging method will be reccomeded for futher tumor assessment.
5. No prior chemotherapy (including hepatic arterial infusion) for colorectal cancer.
   Adjuvant chemotherapy and neoadjuvant chemo-radiotherapy (excluding patients with synchronous liver metastases from lower rectal cancer) are not included in the chemotherapies listed above (however, if oxaliplatin-containing adjuvant chemotherapy has been given, 24 weeks must have elapsed after the last dose of oxaliplatin.
6. No history of surgical resection, radiofrequency ablation or other thermal coagulation therapy, cryotherapy, chemotherapy, or other treatment for liver metastases or other organ metastases.

7. Age at enrollment ≥20 y and ≤80 y.
8. Eastern Cooperative Oncology Group (ECOG) performance status of 0 or 1.
9. Life expectancy of three months or longer from date of enrollment.
10. Major organ function that meets the criteria below within 14 days before enrollment. If multiple test results are available within the particular period, those obtained nearest the time of enrollment will be used, and blood transfusions and use of hematopoietic growth factors or similar products are not permitted within 14 days before the day of testing.
    1) Neutrophil count ≥1,500/mm^3
       (Calculated from the white blood cell count and the neutrophil ratio (%) in the differential white blood cell count)
    2) Platelet count: ≥10.0×10^4 /mm^3
    3) Hemoglobin ≥9.0 g/dl
    4) Total bilirubin ≤2.0 mg/dl
    5) AST, ALT ≤200 IU/I
    6) Serum creatinine ≤1.5 mg/dl
    7) INR <1.5
    8) Urinary protein ≤2+NB)

NB: See the chart below for conversion of proteinuria grade, and qualitative and quantitative values.

Grade	CTCAE v4.0	
	Qualitative	Quantitative
1	1+	<1.0g/24h
2	2+	1.0-3.5g/24h (adult)

11. Personally signed and dated informed consent forms for participation in this study, obtained before enrollment.

4.2. Exclusion Criteria

Patients who meet any of the following criteria will be excluded from the study.

1. History of serious drug hypersensitivity.
2. Receiving treatment with anti-platelet drugs (aspirin products with daily dose ≥325 mg) or NSAIDsNB).
   NB: As-needed use of NSAIDs for cancer pain is permitted.
3. Continuous use of systemic steroids.
4. Surgery or biopsy with skin incision, or traumatic injury sutured within 14 days before enrollment.
   Excludes sutures used for securing an implantable venous port.
5. Serious postoperative complications. (e.g., postoperative infection, ruptured sutures, or paralytic ileus that has not resolved by enrollment)
6. An unequivocal diagnosis of hereditary colorectal cancerNB).
   NB: Familial adenomatous polyposis (FAP) or hereditary non-polyposis colorectal cancer (HNPCC)
7. Active double cancerNB).
   NB: Synchronous double cancer or metachronous double cancer with a disease-free period of five years or less. However, carcinoma in situ found to have been cured by local treatment or lesions corresponding to intramucosal carcinoma are not included in active double cancers. Multiple colorectal cancers are permitted if they are all adenocarcinomas. Cancers with histologic type other than adenocarcinoma will be excluded.
8. Concurrent or previous cerebrovascular disease symptoms within one year before enrollment.
9. Ascites, or pleural or cardiac effusion requiring drainage.
10. Fresh hemorrhage from the gastrointestinal tract, intestinal paralysis, intestinal obstruction, or peptic ulcer.
11. Concurrent or previous gastrointestinal perforation within one year before enrollment.
12. Active infection.
13. HBs antigen positive or HCV antibody positive.
14. Any of the following coexisting diseases.
    1) Poorly controlled hypertension
    2) Poorly controlled diabetes mellitus

   3) Poorly controlled arrhythmia
   4) Other clinically significant disease (e.g., heart disease, interstitial pneumonia, renal failure)

15. Grade 2 or higher diarrhea.
16. Grade 1 or higher peripheral neuropathy.
17. Pregnant women, nursing mothers, women of childbearing potential (intention), and men whose partners wish to become pregnant.
18. Concurrent psychiatric illness or symptoms that may hinder participation in the study.
19. Judged by the subinvestigator to be unsuitable for entry into this study.

5. Enrollment and Assignment

5.1. Enrollment Procedures

Patient enrollment in this study will be performed using a web-based enrollment system.

5.1.1. Prior Acquisition of User ID and Password

To enroll patients, a user ID and password are required to log in to the web-based enrollment system. After approval by the ethics committee or IRB, the investigator or subinvestigator at the study site who will enroll patients obtains a personal user ID and password from the data center.

5.1.2. Enrollment

After checking that the candidate meets all the inclusion criteria and none of the exclusion criteria, the web-based enrollment system is accessed via the Internet. Patient enrollment is possible for 24 hours a day, except during system inspection or maintenance downtime. In accordance with the instructions in the enrollment system, the required entries are completed to enroll the patient.

Web-based enrollment: 24-hour enrollment (including Saturdays, Sundays, and national holidays)
https://edmsweb11.eps.co.jp/atom/

Contact for inquiries on web-based enrollment
Data Center: DM Center 1, Clinical Information Division, EPS Corporation
Tel: 03-5684-7767
E-mail: prj-atomdc@eps.co.jp
Monday to Friday, 0900 to 1700 (excluding Saturdays, Sundays, national holidays, and December 29 to January 4)

Contact for inquiries on patient selection criteria
Study Secretariat: Yasunori Emi
Satomi Abe
abesato@surg2.med.kyushu-u.ac.jp
Department of Surgery and Science, Graduate School of Medical Sciences, Kyushu University
Department of Surgery II
3-1-1 Maidashi, Higashi-ku, Fukuoka, Fukuoka Prefecture 812-8582.
Tel: 092-642-5466

5.1.3. Issuance and Notification of Enrollment Results

(1) An assigned group and enrollment number will be issued as evidence of enrollment.
(2) After eligibility is confirmed, enrollment is completed with the display of an enrollment number on the enrollment screen. Next, a confirmation of enrollment results is printed, which, after the contents are checked, must be retained in the medical records or other archives.

5.1.4. Special Notes for Enrollment

1. Patients cannot be enrolled after starting the protocol treatment, without exception (major violation).
2. If the protocol chemotherapy and protocol surgery components of the protocol treatment are to be conducted at different sites, separate rules will be established
3. All data required for enrollment are essential and must be true and correct. If a false enrollment is discovered after the fact, it will be treated as a major violation. The subsequent handling of such patients will be decided by the study secretariat, study coordinating committee, and efficacy and safety evaluation committee.
4. The protocol treatment must not be started before the results of enrollment have been decided. The protocol treatment is to be started promptly after completion of enrollment (as a general rule, within 14 days). If it is not possible to start within 14 days, the reason should be reported.
5. If the entered data are inadequate, enrollment cannot be accepted until all requirements have been met.
6. If an enrollment error or multiple enrollment is discovered, the study secretariat should be notified promptly. In every instance of multiple enrollment, the first enrollment information (enrollment number, assigned group) will be used.
7. Excluding instances of withdrawal of consent, which include rejection of the utilization of data for research

purposes, enrollment must not be canceled after a patient has been enrolled once.

8. The body surface area calculated from the height and weight at enrollment, and the planned dose of each drug must be double-checked against those of the subinvestigator. At the study site too, the figures must be calculated and checked.
9. The "date of enrollment" is defined as the day on which the results of enrollment (assigned group, enrollment number) were obtained. If a candidate is assessed as ineligible, this will be the "date of assessment of ineligibility", rather than the "date of enrollment".

5.1.5. Screening Report

If the candidate subject does not meet “inclusion criteria” or meet “exclusion criteria”, the subject can be reported to screening web system. In this case, the investigator need not to give informed consent for the candidate subject because of no personally identifiable information. For detail information, SOP will be arranged.

5.2. Randomized Assignment and Adjustment Factors

In the enrollment procedure, the treatment group will be randomly assigned using the assignment program in the web-based enrollment system.

Patients will be randomly assigned to groups, minimizing bias by use of the following adjustment factors: (i) Synchronous liver metastases, primary lesion present/synchronous liver metastases, primary lesion absent/metachronous liver metastases; (ii) number of metastases (1-4/more than 5); (iii) maximum diameter of metastatic lesions (smaller than 5 cm/5 cm or bigger); and (iv) oxaliplatin use in adjuvant chemotherapy - yes/no. Details of the randomized assignment method will not be disclosed to the researchers at the participating study sites.

5.2.1. [Eligible Patients] Enrollment to Protocol Treatment

=Liver

=Primary

CTx protocol chemotherapy

Liver resection

Postoperative CTx

Recurrence

Primary resection

Simultaneous primary and liver resection

Single-stage resection

Two-stage resection

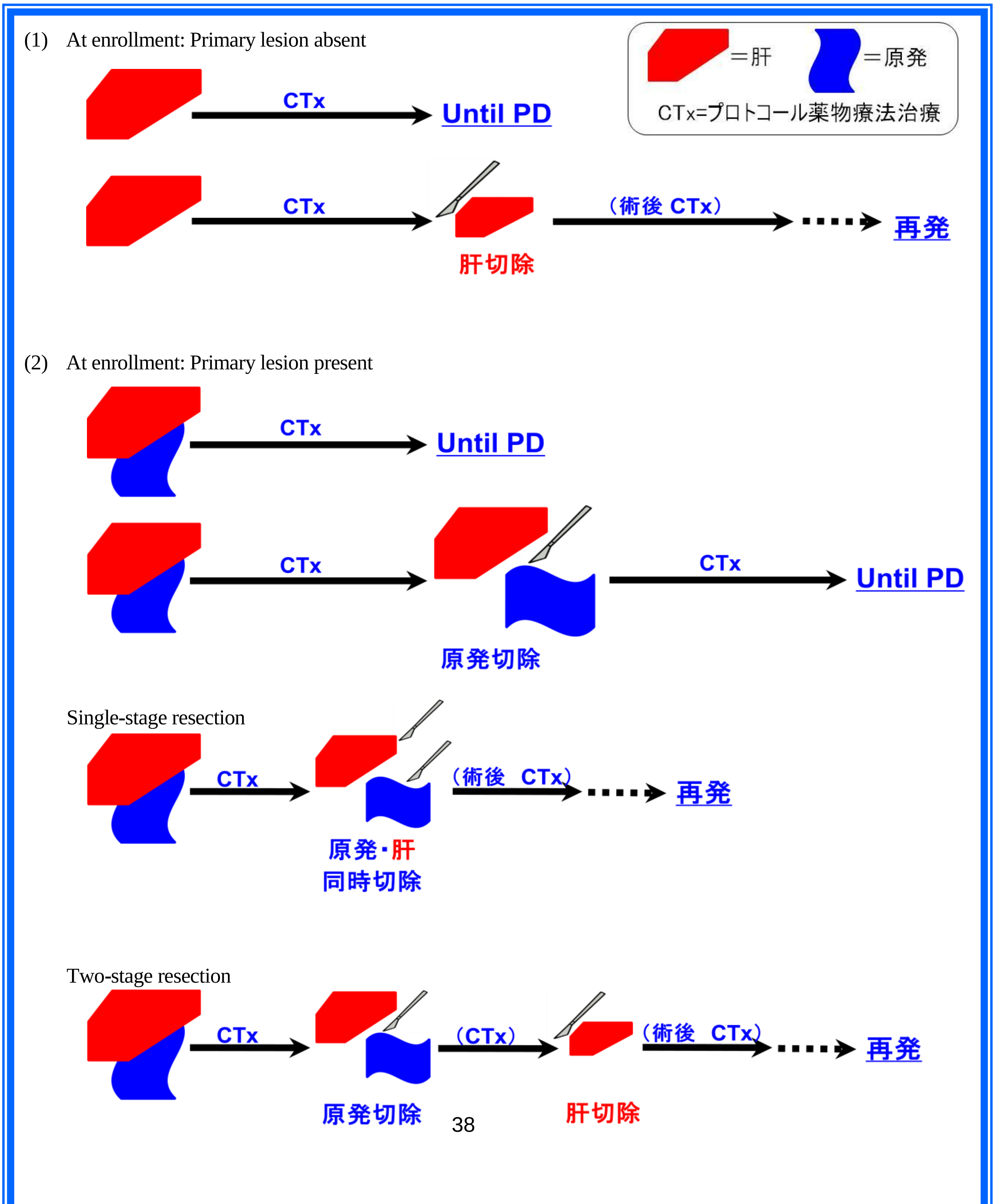

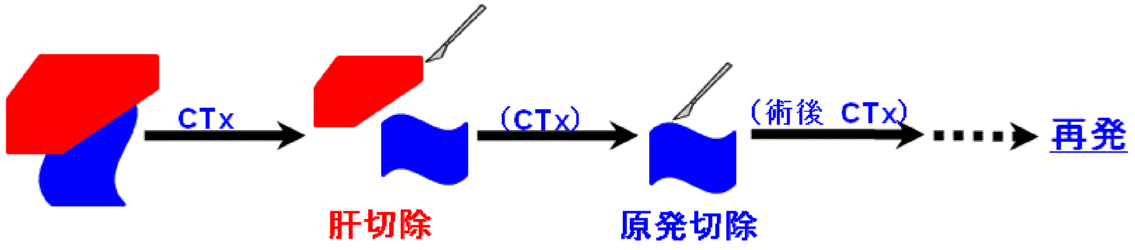

CTx
(CTx)
(術後 CTx)
再発
肝切除
原発切除

6. Treatment Plan and Criteria for Amending Treatment

6.1. Protocol Treatment

As initial therapy for *KRAS* wild-type liver-only metastases from colorectal cancer, the protocol chemotherapy and protocol surgery will be defined as the protocol treatment. Patients will be randomly assigned to either of the protocol chemotherapy options: mFOLFOX6 plus bevacizumab (Group A) or mFOLFOX6 plus cetuximab (Group B).

Within 14 days after enrollment, the protocol chemotherapy will be started for patients assigned to Group A or Group B. If starting within 14 days is not possible, the reason should be reported. Patients can be either inpatients or outpatients.

If the protocol chemotherapy and protocol surgery are conducted at different sites, separate rules will be established.

6.2. Definition of Protocol Chemotherapy

The protocol chemotherapy must not contravene the provisions of sections 6.4. "Scope of Protocol Chemotherapy", 6.5. "Criteria for Discontinuation of Protocol Treatment", and 6.6. "Criteria for Amendment of Treatment".

6.2.1. Group A: mFOLFOX6 plus bevacizumab

The regimen described below will be delivered in a two-week cycle, and as a rule, continued until progression (not RECIST progression, but clinical progression that necessitates a change in treatment).

Radiology review will be performed every eight weeks (±one week), at which the therapeutic response and suitability for liver resection will be evaluated. If the liver is deemed to be resectable, liver resection will be performed as the protocol surgery. However, if it is decided at the first radiology review that liver resection is feasible, the protocol surgery (liver resection) must be performed after delivering up to eight cycles of protocol chemotherapy. (If an evaluation of SD with increasing size is made or the lesion has shrunk, but liver function values are showing a worsening tendency at the first radiology review, liver resection is permitted.)

For patients who undergo liver resection, the delivery of a total of 12 cycles of the same neoadjuvant chemotherapy and adjuvant chemotherapy is recommended.

However, priority can be given to the decision of the subinvestigator as to the number of cycles of post-operative adjuvant chemotherapy. If more than 12 cycles of protocol chemotherapy have been delivered before resection, the decision on adjuvant chemotherapy will be the sole responsibility of the subinvestigator.

Otherwise, priority can be given to the decision of the subinvestigator on the performance of the protocol surgery and the continuation of protocol chemotherapy.

(However, this may be shortened or the start delayed, depending on whether there is a public holiday, or in consideration of the patient's personal circumstances regarding site visits.)

Table: mFOLFOX6 plus Bevacizumab Therapy

Drug	Dose	Dosing method		Day of dosing
Bev	5 mg/kg	div	90 → 60 → 30 min recommended[NB)]	Day 1
L-OHP	85 mg/m^2	div	120 min	Day 1
l-LV	200 mg/m^2	div	120 min	Day 1
5-FU	400 mg/m^2	iv	Within 15 min	Day 1
5-FU	2400 mg/m^2	civ	46 hr	Days 1-2

NB: The duration of infusion of bevacizumab can be determined at the discretion of the subinvestigator.

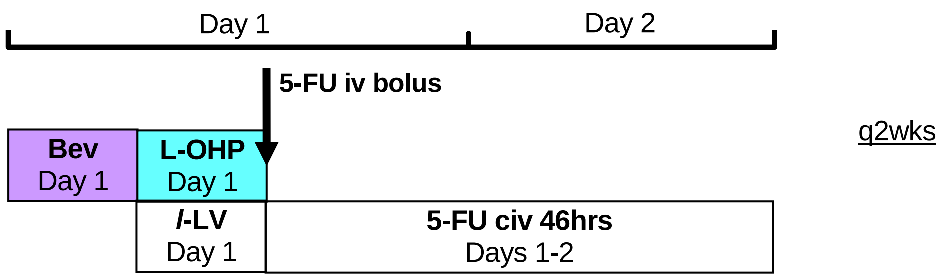
Day 1
Day 2
5-FU iv bolus
Bev
Day 1
L-OHP
Day 1
l-LV
Day 1
5-FU civ 46hrs
Days 1-2
q2wks

[Doses of mFOLFOX6 plus bevacizumab]

1. As a general rule, the doses will be calculated using the body surface area and body weight at enrollment, and any fractional doses calculated will be rounded as shown below. Depending on the changes in body weight at the start of each cycle, the dose will not be recalculated (the dose may be reduced because of Grade 2 or higher weight loss as an adverse event).

   Bev :Round to the nearest 25 mg.

   L-OHP :Round to the nearest 5 mg.

   5-FU :Round to the nearest 50 mg.

   I-LV :Round to the nearest 10 mg.

   As a general rule, the body surface area will be calculated using the DuBois formula.

   Body surface area (m^2) = Body weight (kg)$^{0.425}$×Height (m)$^{0.725}$×0.007184
2. Adjustment of the doses for pronounced changes in body weight after starting treatment shall be ultimately made at the discretion of the subinvestigator.

[Special notes on administration of bevacizumab]

1. Paying close attention to findings of concern such as infusion reaction, the subinvestigator can determine duration of infusion at his or her discretion.
2. The patients with synchronous liver metastases for whom primary resection has been performed before enrollment (and similar situations) or for patients for whom colostomy has been performed, the patients can be regitsored later than 14 days after finishing the surgery. In case of administration within 28 days after surgery, the first cycle of bevacizumab should not be administered.

   (Because the exclusion criteria stipulate a minimum 28-day gap between the day of primary resection and the day of enrollment, this is not essential. Whether the first dose is omitted or not, infusion of bevacizumab is mandatory from the second cycle onwards.)

6.2.2. Group B: mFOLFOX6 plus Cetuximab

The regimen described below will be delivered in a two-week cycle, and as a rule, continued until progression (not RECIST progression, but clinical progression that necessitates a change in treatment).

Radiology review will be performed every eight weeks (±one week), at which the therapeutic response and suitability for liver resection will be evaluated. If the liver is deemed to be resectable, liver resection will be performed as the protocol surgery. However, if it is decided at the first radiology review that liver resection is feasible, the protocol surgery (liver resection) must be performed after delivering up to eight cycles of protocol chemotherapy. (If an evaluation of SD with increasing size is made or the lesion has shrunk, but liver function values are showing a worsening tendency at the first radiology review, liver resection is permitted.)

For patients who undergo liver resection, the delivery of a total of 12 cycles of the same neoadjuvant chemotherapy and adjuvant chemotherapy is recommended.

However, priority can be given to the decision of the subinvestigator as to the number of cycles of post-operative adjuvant chemotherapy. If more than 12 cycles of protocol chemotherapy have been delivered before resection, the decision on adjuvant chemotherapy will be the sole responsibility of the subinvestigator.

Otherwise, priority can be given to the decision of the subinvestigator on the performance of the protocol surgery and the continuation of protocol chemotherapy.

(However, this may be shortened or the start delayed, depending on whether there is a public holiday, or in consideration of the patient's personal circumstances regarding site visits.)

Table: mFOLFOX6 plus Cetuximab

Drug	Dose	Route		Day of dosing
Cetuximab	250 mg/m^2*	div	60 min*	Days 1, 8
L-OHP	85 mg/m^2	div	120 min	Day 1
l-LV	200 mg/m^2	div	120 min	Day 1
5-FU	400 mg/m^2	iv	Within 15 min	Day 1
5-FU	2400 mg/m^2	civ	46 hr	Days 1-2

* Initially 400 mg/m^2 div, given over 120 minutes

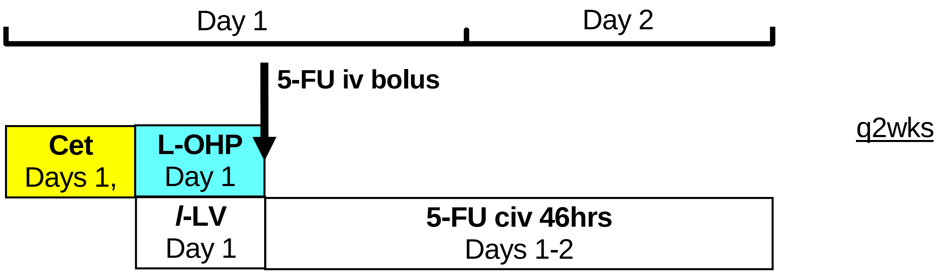

[Doses of mFOLFOX6 plus cetuximab]

1. As a general rule, the doses will be calculated using the body surface area and body weight at enrollment, and any fractional doses calculated will be rounded as shown below. Depending on the changes in body weight at the start of each cycle, the dose will not be recalculated (the dose may be reduced because of Grade 2 or higher weight loss as an adverse event).

   Cet : Round to the nearest 5 mg.

   L-OHP : Round to the nearest 5 mg.

   5-FU : Round to the nearest 50 mg.

   I-LV : Round to the nearest 10 mg.

   As a general rule, the body surface area will be calculated using the DuBois formula.

Body surface area (m^2) = Body weight (kg)$^{0.425}$×Height (m)$^{0.725}$×0.007184

2. Adjustment of doses for pronounced changes in body weight after starting treatment shall be ultimately made at the discretion of the subinvestigator.

[Special notes on administration of cetuximab]

1. Method of preparation of infusion solution and dosing rate

   The required amount of cetuximab is drawn into a syringe, then without dilution or diluted with physiological saline (JP) using an infusion bag or similar, administered by intravenous infusion over a two-hour period the first time, and over a one-hour period for the second and subsequent times. After completion of infusion, the line should be flushed with physiological saline (JP) at the same rate of infusion as that used for cetuximab.

2. Prevention of infusion reactions

   To reduce infusion reactions, premedication with an antihistamine or corticosteroid hormone before administration of cetuximab is recommended.

3. If a Grade 3 or higher infusion reaction occurs

   Infusion of cetuximab must be stopped immediately and not restarted.

   Depending on symptoms, epinephrine, bronchodilators, antihistamines, corticosteroid hormones, intravenous fluids, vasopressors, or other drugs, or inhaled oxygen therapy may be started.

4. If a Grade 1 or 2 infusion reaction occurs

   Infusion should be temporarily halted, and depending on symptoms, epinephrine, corticosteroid hormones, antihistamines, or other drugs, or inhaled oxygen therapy may be started. After symptomatic improvement, infusion is restarted with the rate of infusion reduced as appropriate. In subsequent cycles too, cetuximab should be infused at the reduced rate. If an infusion reaction recurs after reducing the rate of infusion, infusion should be halted and not restarted.

6.3. Definition of Protocol Surgery

The protocol surgery shall be defined as the specified primary resection and liver resection surgeries performed within the scope of continuation of the protocol chemotherapy (during continuation of the protocol treatment). While not recommended in the protocol, the protocol liver resection could be performed several times. Primary resection and liver resection procedures carried out after discontinuation (planned completion) of the protocol chemotherapy will be regarded as ongoing treatment.

6.3.1. Primary Resection

(If a primary lesion is present with synchronous liver metastases, primary resection before enrollment will not be subject to the requirements of this section)

For both Group A and Group B, primary resection may be carried out during the protocol chemotherapy, as appropriate, at the discretion of the subinvestigator. When the protocol chemotherapy is halted for longer than the prescribed time because of this surgery, the discontinuation criteria for protocol chemotherapy are not applicable.

For Group A, however, primary resection will be carried out at least 42 days after the last dose of bevacizumab, administering mFOLFOX6 and omitting bevacizumab from the protocol treatment immediately before surgery. <u>As a general rule, adjuvant chemotherapy will be started after at least 28 days have elapsed following surgery. If it starts within 28 days after surgery, the dose of bevacizumab will be omitted until at least 28 days have elapsed after surgery.</u>

The responsibility will lie solely with the subinvestigator to decide whether to perform both resections simultaneously, or to perform the primary resection first or the liver resection first. As well, the interval between the surgeries shall be decided by the subinvestigator and is not specified in the protocol. However, the degree of residual tumor (Rx) if primary resection is carried out with liver resection should be assessed after completion of the first liver resection and primary tumor resection.

At the discretion of the subinvestigator, as appropriate, it is possible to undertake primary resection in circumstances where liver resection is not scheduled (in this case too, it will be judged as being within the scope of protocol surgery).

Fundamentally, chemotherapy is not recommended between the liver resection and primary resection, but in unavoidable circumstances, it will be permitted (the reason should be reported). Naturally, it is mandatory that the treatment remain within the scope of the prescribed protocol chemotherapy.

If it is discovered intraoperatively that peritoneal dissemination or distant lymph node metastases, or other metastatic lesions or regional lymph node metastases including the primary lesion makes radical resection impossible, the subinvestigator will decide on the appropriate operative technique, which will not be specified in the protocol (the subinvestigator's decision can range from exploratory laparotomy to combined resection).

New lesions that were not identified on diagnostic imaging before surgery will not be classified as new lesions or events for the purpose of PFS, the primary endpoint of this study, even if ascertained intraoperatively (including intraoperative diagnostic imaging only). Above all, the PFS assessment shall be made only from the specified radiological findings (contrast-enhanced CT of the trunk, etc.).

6.3.2. Liver Resection

For both Group A and Group B, radiology review will be performed every eight weeks (±one week), and an assessment made of the therapeutic response and as to whether liver resection is appropriate. If it is decided that liver resection is possible in accordance with the criteria below, liver resection will be performed by either systematic resection or partial resection. Preoperative portal embolization and portal vein ligation at primary resection are permitted . Radiofrequency ablation or other form of coagulation therapy may not be added before liver resection. If radiofrequency ablation or other form of coagulation therapy becomes unavoidable intraoperatively during liver resection, the evaluation of the residual lesion after tumor resection will be classified as R2.

The decision on "liver resectability" before surgery in this study will not include "a plan for two-stage liver resection". However, if the subinvestigator decides that "a plan for two-stage liver resection " is necessary in the subject's interests, such a decision is permitted in this study. If it is decided preoperatively that single-stage liver resection is possible and it is found necessary from the intraoperative findings to proceed with split surgery in two stages, the same surgical plan can be used at the discretion of the subinvestigator (in the clinical study, for patients with unequivocal R2 residual tumor, the patient will be judged to have undergone a liver resection additional to the protocol, within the scope of continuing the protocol treatment). In patients with "remnant liver recurrence during adjuvant chemotherapy after protocol liver resection", patients in whom liver resection is feasible may undergo additional repeat liver resection as protocol surgery.

If it is decided that liver resection is possible, the ICG R15 min test must be performed, and the surgical technique considered with reference to the Makuuchi criteria[54)].

However, if it is decided at the first radiology review (after eight weeks) that liver resection is feasible, liver resection must be performed after delivering up to eight cycles of protocol chemotherapy. If an evaluation of SD with increasing size is made or the lesion has shrunk, but liver function values are showing a worsening tendency at the first radiology review, this is not applicable. If liver resection is performed before the specified number of cycles have been delivered, the reason should be reported. For patients for whom it is not possible to make an assessment that the liver is resectable by the end of eight cycles, the protocol liver resection can be performed for patients if it is concluded that the liver is resectable, as long as the protocol chemotherapy is being continued. However, the risk to liver resection caused by the increased number of cycles of protocol chemotherapy is to be assessed at the discretion of the subinvestigator. After the discontinuation or planned completion of the protocol chemotherapy, it is also possible to proceed with liver resection as ongoing treatment.

For both Group A and Group B, if it is decided that liver resection is possible, the procedure will be performed within eight weeks from the date of the decision. The protocol chemotherapy must be continued until 28 days before liver resection.

If liver resection is to be performed in Group A, mFOLFOX6 should be administered with bevacizumab omitted from the protocol chemotherapy before surgery, and with the day of administration of mFOLFOX6 designated as Day 1, surgery should be performed on Day 15, or Day 29 or thereafter.

(Counting from the day of the last dose of bevacizumab as Day 1, liver resection should be performed from Day 43 onwards.) If the protocol chemotherapy is halted for longer than the prescribed time because of this surgery, the discontinuation criteria for chemotherapy are not applicable.

	Drug administration					Surgery
Bevacizumab	↑	↑	• • •	↑	◀— Interval 42 days —▶	Liver resection performed

mFOLFOX6	↑	↑	・<br>・<br>・	↑	↑	(↑)	Interval<br>←→<br>14 days	

If liver resection is to be performed in Group B, it should be performed from Day 15 onwards, counting the day of administration of mFOLFOX6 plus cetuximab as Day 1.

	Drug administration						Surgery
Cetuximab	↑	↑	・・・	↑	↑	Interval<br>14 days<br>←→	Liver resection performed
mFOLFOX6	↑	↑	・・・	↑	↑		

In other situations, priority can be given to the decision of the subinvestigator on the timing of liver resection and the continuation of protocol chemotherapy.

Fundamentally, chemotherapy is not recommended between liver resection and primary resection, but will be permitted in unavoidable circumstances (the reason should be reported). Of course, it is mandatory that the treatment be within the scope of the specified protocol chemotherapy.

[Criteria for deciding on liver resectability]

The decisions on liver resectability will be reported for all patients in accordance with the guidelines.

At the time of preparation for liver resection by the subinvestigator for patients for whom protocol liver resection is planned (including refusal of surgery, consequent exploratory laparotomy, etc.). For patients for whom liver resection is not planned, reports will be made on the "decision on liver resectability" at the time of completion of eight cycles of protocol chemotherapy or at discontinuation for those with fewer than eight cycles and who have discontinued the protocol chemotherapy.

If the following criteria are met in a preoperative diagnostic procedure within six weeks before liver resection, the liver will be regarded as resectable.

1. Patients with no new lesions occurring as distant extrahepatic metastases, and for whom it is decided that single-stage resection will be possible without remnant liver metastases.

   This excludes patients for whom resection is deemed to be possible by proceeding in a planned manner, by local therapy such as radiofrequency ablation before liver resection or two-stage liver resection. However, if the subinvestigator concludes that "a plan for two-stage liver resection" is necessary in the subject's interests, other therapies are permitted in this study.
2. Patients for whom it is decided that post-resection remnant liver function can be adequately preserved
3. Patients with none or controllable ascites
4. Patients with ECOG performance status 0 or 1
5. Patients with preserved major organ function
   1) Neutrophil count $\geq$1,000/mm^3
   2) Platelet count: $\geq$80,000$\times$10^3/mm^3
   3) AST and ALT: $\leq$200 IU/L
   4) Serum total bilirubin: $\leq$2.0 mg/dl
   5) Serum creatinine: $\leq$1.5 mg/dl
6. Patients for whom the subinvestigator believes that there are no concerns with indications for surgery

* Informed consent for surgery given by the patient in person: The report should include whether the explanation

of informed consent was given with the patient classified as having a "resectable liver". Whether the person has given consent for that to occur should be documented.

For patients who exceed the above "liver resectability" criteria, this study protocol will permit that treatment if the subinvestigator deems that "resection" is necessary in the best interests of the patient (risk and effectiveness).

[Surgical technique for liver resection - selection criteria (Makuuchi criteria[54])]

If it is decided that liver resection is possible, the ICG R15 min test must be performed, and the surgical technique considered with reference to the Makuuchi criteria[54].

Ascites
None or controllable
Total bilirubin
Not controllable
Normal
Partial resection
Enucleation
No indication for surgery
Right liver resection
Left hepatic trisegmentectomy
Segmentectomy
Left liver resection
Subsegmentectomy
Partial resection
Detection

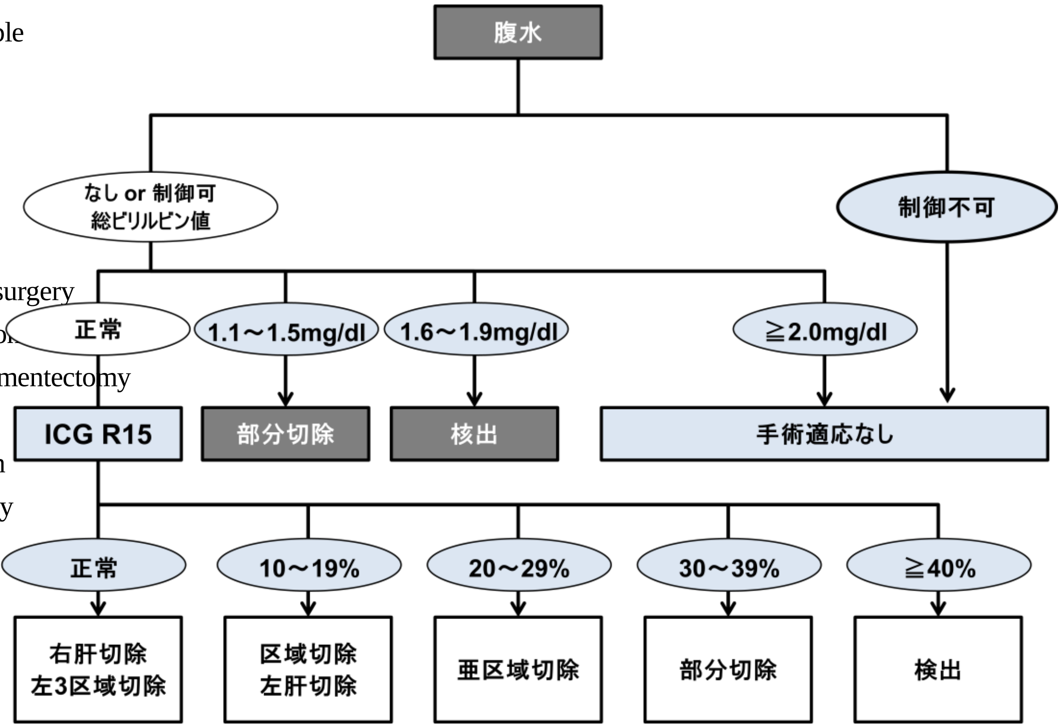

[Preparation and submission of pathological specimens after liver resection]

To perform the pathologic reviews specified in 11.4. "Exploratory Endpoints" (1) Tumor regression

grade (TRG), (2) modified Tumor Regression Grade (mTRG), (3) Dangerous halo (HALO), (4) Sinusoidal obstruction syndrome (SOS), and (5) Other, specimens should be submitted to the data center (21.12).

[A separate set of procedures will be prepared and established for the preparation and submission of pathologic specimens.]

6.3.3. Decisions During Liver Resection

As far as possible lesions will be explored intraoperatively, and at the discretion of the subinvestigator, liver resection will be performed by either systematic resection or partial resection. Radiofrequency ablation and other forms of local therapy are not permitted before liver resection, but if radiofrequency ablation or another form of local therapy becomes unavoidable during surgery, the evaluation of residual tumor after resection will be R2. If it is feasible to do single-stage liver resection before surgery, but intraoperative findings lead to the decision that two-stage surgery is necessary, the same surgical plan can be adopted at the discretion of the subinvestigator. As a clinical study, for patients with unequivocal R2 residual tumor, the patient will be judged to have undergone a liver resection additional to the protocol, within the scope of continuing the protocol treatment. If the protocol treatment is discontinued and liver resection is performed after a drug or therapy not specified in the protocol is performed, it will be concluded that liver resection has been performed as ongoing treatment.

If it is discovered intraoperatively that peritoneal dissemination or distant lymph node metastases, or other metastatic lesions or regional lymph node metastases including the primary lesion makes radical resection impossible (excluding regional lymph node metastases that include a curatively resectable primary lesion), the subinvestigator will decide on the appropriate operative technique, which will not be specified in the protocol (the subinvestigator's decision can range from exploratory laparotomy to combined resection). For resections in which metastasis-positive hepatic hilar lymph nodes are discovered for the first time intraoperatively, the decision on how to proceed will be the subinvestigator's, and will not be specified in the protocol. The addition of cholecystectomy is also not specified in the protocol.

New lesions that were not identified on diagnostic imaging before surgery will not be handled as new lesions or events for the purpose of PFS, the primary endpoint of this study, even if discovered on intraoperative findings (including intraoperative diagnostic imaging only). Above all, the PFS assessment shall be made only from the specified radiological findings (contrast-enhanced CT of the trunk, etc.).

6.3.4. Adjuvant Chemotherapy after the Protocol Surgery (primary lesion and liver resection)

If the protocol surgery (primary lesion and liver resection) has been performed in either Group A and Group B, the assigned therapy for Group A or Group B should be delivered for up to 12 cycles, comprising neoadjuvant and adjuvant chemotherapy combined.

However, priority shall be given to the decision of the subinvestigator on the number of cycles of adjuvant chemotherapy following the protocol surgery (primary lesion and liver resection). If more than 12 cycles of protocol chemotherapy have been delivered before resection, the subinvestigator will be solely responsible for the decision of whether to give adjuvant chemotherapy, and the planning for the number of cycles to be given if that is the case.

When planned adjuvant chemotherapy has been completed after R0 or R1 resection, it will be concluded that the protocol treatment (planned completion) has been discontinued.

For Group A, as a general rule, adjuvant chemotherapy will be started after at least 28 days have elapsed following surgery. If it is started within 28 days after surgery, bevacizumab will be omitted until at least 28 days after surgery has elapsed.

If the response to the protocol chemotherapy before resection is inadequate (PD or SD, but with a tendency to increase in size, etc.), the protocol treatment should be discontinued and another chemotherapy can be selected for the

adjuvant chemotherapy as ongoing treatment. The day of discontinuation of protocol treatment in this situation shall be defined as the day of protocol surgery (the day of primary resection or day of liver resection, whichever is the later).

If assessments of SD and inadequate response are made, liver resection performed and the protocol treatment discontinued, the protocol-specified diagnostic imaging should be continued and the patient's clinical course monitored until recurrence, without making a decision on censoring as an event for the purpose of the primary endpoint, PFS.

6.4. Scope of Protocol Chemotherapy

In both groups, the scope of protocol chemotherapy is defined as follows.

6.4.1. Group A: mFOLFOX6 plus bevacizumab

1. If bevacizumab only is held or discontinued and mFOLFOX6 is continued, included in the definition of protocol chemotherapy.
2. If oxaliplatin is held or discontinued and sLV5FU2 plus bevacizumab is continued, included in the definition of protocol chemotherapy.
3. If bevacizumab and oxaliplatin are held or discontinued and sLV5FU2 are continued, included in the definition of protocol chemotherapy.
4. If bolus infusion of 5-FU only is held or discontinued, included in the definition of protocol chemotherapy.
5. None of the following are permitted: bevacizumab monotherapy, oxaliplatin monotherapy, or oxaliplatin plus bevacizumab.
6. The dose of each drug shall be at least the specified level (including the allowable ranges).

Changes to treatment	Definition of protocol chemotherapy
mFOLFOX	○
sLV5FU2 + Bevacizumab	○
sLV5FU2	○
Omit 5-FU intravenous bolus	○
Bevacizumab monotherapy, oxaliplatin monotherapy	×
Oxaliplatin + bevacizumab	×

6.4.2. **Group B**: mFOLFOX6 plus cetuximab

1. If cetuximab only is held or discontinued and mFOLFOX6 is continued, included in the definition of protocol chemotherapy.
2. If oxaliplatin is held or discontinued and sLV5FU2 plus cetuximab is continued, included in the definition of protocol chemotherapy.
3. If cetuximab and oxaliplatin are held or discontinued and sLV5FU2 is continued, included in the definition of protocol chemotherapy.
4. If bolus infusion of 5-FU only is held or discontinued, included in the definition of protocol chemotherapy.
5. If mFOLFOX6 is held or discontinued and cetuximab monotherapy is continued, included in the definition of protocol chemotherapy.
6. Neither oxaliplatin monotherapy nor oxaliplatin plus cetuximab therapy is permitted.
7. The dose of each drug shall be at least the specified level (including the allowable ranges).

Changes to treatment	Definition of protocol chemotherapy
mFOLFOX	○
sLV5FU2 + Cetuximab	○
sLV5FU2	○
Omit 5-FU intravenous bolus	○
Cetuximab monotherapy	○
Oxaliplatin monotherapy	×
Oxaliplatin + Cetuximab	×

6.5. Criteria for Discontinuation of Protocol Treatment

If any of the following is applicable, the protocol treatment will be discontinued. Protocol treatment indicates both protocol chemotherapy and protocol surgery.

1. If the response to the protocol chemotherapy is inadequate and the subinvestigator deems a change in chemotherapy is necessary

   If the subinvestigator deems that a change in chemotherapy is necessary because of situations such as "worsening" after the protocol treatment described in section 6.1 has been performed, or "poor response to protocol chemotherapy before liver resection" or "recurrence during adjuvant protocol chemotherapy after liver resection". In patients with "remnant liver recurrence during adjuvant chemotherapy after protocol liver resection", patients in whom liver resection is feasible may undergo liver resection as protocol surgery.

   "Progression" includes both PD based on diagnostic imaging (RECIST-PD, etc.) and progression of the primary disease in accordance with a clinical judgment that is independent of diagnostic imaging (clinical progression). If a clinical judgment is made of "not progression requiring a change of treatment", even with an assessment of PD in accordance with the RECIST response assessment criteria, the clinical judgment takes precedence and it is not necessary to discontinue the protocol treatment. If a clinical judgment is made of clinically unequivocal progression and it is deemed necessary to change the treatment, even without an assessment of PD in accordance with the response assessment criteria, the clinical judgment takes precedence and the protocol treatment will be discontinued.

   However, if it is concluded that the protocol chemotherapy is "worsened", "PD", "inadequate response", or "recurrence during treatment", the protocol surgery can be performed without an assessment that the protocol treatment has been discontinued, in a situation in which the protocol surgery (primary resection or liver resection) is possible.

2. If the protocol treatment described in section 6.1 is provided and surgery performed, that includes liver resection, and it is judged to be follow-up after the planned completion of adjuvant chemotherapy or without adjuvant chemotherapy

3. If the protocol chemotherapy cannot be continued because of adverse events

   1) If a Grade 4 non-hematologic toxicity (Japanese translation of CTCAE Ver. 4.0) occurs.

   2) If the start of the protocol chemotherapy is delayed for more than 28 days at least twice because of adverse events.

      (One delay is permissible. Delays may be permissible, depending on whether there is a public holiday, or in consideration of the patient's personal circumstances regarding site visits.)

   3) If the requirements for discontinuation of the protocol chemotherapy in section 6.6. "Criteria for Amendment of Treatment" are met

   4) If the subinvestigator decides that discontinuation is necessary because of an adverse event, outside the treatment amendment criteria

4. If the patient personally requests discontinuation of the protocol treatment for reasons related to adverse events

5. If the patient personally requests discontinuation of the protocol treatment for reasons that are <u>not</u> related to adverse events

   (This category is used only for situations in which a relationship with adverse events can be excluded, such as change of residence of patient or family, or economic reasons, etc.)

6. If the patient personally withdraws consent for participation in this study

   (A withdrawal of consent form and communication from the patient to the study secretariat are essential)

   ① If the data can be used

   ② If some of the data can be used (up to the time that the patient requests discontinuation)

   ③ If the data cannot be used and the enrollment is canceled

7. Death during protocol therapy
   (Death before a decision to discontinue the protocol treatment for another reason)
8. Otherwise, if there is progression before starting treatment after enrollment (the protocol treatment could not be started because of rapid progression), of if discontinuation is necessary because a serious protocol violation has been discovered, and in situations such as when ineligibility is discovered because of a factor such as change in pathologic diagnosis after enrollment (not only in the judgment of the subinvestigator, communication with the study secretariat is essential)

The date of discontinuation of protocol treatment will be regarded as the date of death in situation (7), and the date of surgery if the protocol treatment is discontinued with the "protocol surgery" in situation (1) or (2). If the adjuvant chemotherapy is to be completed as planned, Day 14 of that last cycle. In other situations, the date of discontinuation shall be defined as the day on which the subinvestigator decided to discontinue the protocol chemotherapy.

6.6. Criteria for Amendment of Protocol Chemotherapy

The following terminology will be used for amendment criteria.

Discontinuation	Part or all of the treatment is ended prematurely without restarting. The planned completion of adjuvant chemotherapy is included in the definition of discontinuation.
Treatment hold	Treatment hold is defined as a single holiday of at least one dose of the investigational products, and waiting for the restart conditions to be met.
Treatment delay	Prolongation of the dosing interval, representing a delay from the prescribed dosing time.<br>If the start of the protocol chemotherapy is delayed at least twice for a total duration of more than 28 days, it will be regarded as discontinuation of protocol chemotherapy.<br>A single delay exceeding 28 days will allow the protocol chemotherapy to be continued.<br>(However, delays may be permissible, depending on whether there is a public holiday, or in consideration of the patient's personal circumstances regarding site visits.)

6.6.1. Drug Dose Levels

Doses for mFOLFOX6 and cetuximab shall be adjusted with reference to the dose reduction chart shown below. In situations such as those when starting or delaying treatment is repeated because of a hematologic toxicity or another event, the subinvestigator can reduce the dose level either within or outside the dose reduction criteria, at his or her discretion.

The dose of bevacizumab will be fixed at 5 mg/kg and will not be adjusted.

If the dosing period for cetuximab is extended because of a dose hold or delay, it will be restarted from the starting dose (loading dose) of 400 mg/m^2, and thereafter it can be returned to 250 mg/m^2.

[Dose Reduction Chart]

	5-FU (bolus) NB1)	5-FU (Infusion)	Oxaliplatin	Bevacizumab	Cetuximab
**Specified dose (Dose level 0)**	**400 mg/m^2**	**2,400 mg/m^2**	**85 mg/m^2**	**5 mg/kg**	**250 mg/m^2 (First dose: 400mg/m^2)**
**-1 Level (Dose reduced 1 level)**	300 mg/m^2	2,000 mg/m^2	65 mg/m^2	5 mg/kg	200 mg/m^2
**-2 Levels [illegible]**	[illegible]	[illegible]	[illegible]	[illegible]	[illegible]

NB1. If the subinvestigator concludes that diarrhea or a hematologic toxicity is attributable to 5-FU (bolus), it is possible to omit the 5-FU (bolus) component of mFOLFOX6.

Bev :Round to the nearest 25 mg.

Cet :Round to the nearest 5 mg.

L-OHP :Round to the nearest 5 mg.

5-FU :Round to the nearest 50-mg.

I-LV :Round to the nearest 10 mg.

As a general rule, the body surface area will be calculated using the DuBois formula.

Body surface area (m^2) = Body weight (kg)$^{0.425}$×Height (m)$^{0.725}$×0.007184

6.6.2. Group A: Criteria for Dose Reduction, Hold, and Discontinuation of mFOLFOX6 plus Bevacizumab

For the first cycle, check that the criteria for starting a treatment cycle (see 6.6.4) are met, and start from dose level 0.

From the next cycle onwards, check that the criteria for starting a treatment cycle (see 6.6.4) are met and reduce the dose one level, as shown below, in response to the adverse event that occurred in the previous cycle. **However, if the dose has already been reduced because of oxaliplatin-induced neuropathy, the dose of only the drug without a previous dose reduction can be reduced one level.**

At the discretion of the subinvestigator, the starting dose for the next cycle can be reduced two levels at one time after the occurrence of clinically significant adverse events. Dose reduction up to two levels (dose reduction to level -2) may be permitted at the discretion of the subinvestigator for each of Bmab, FL, and L-OHP, and if a three-level dose reduction is deemed necessary, the protocol chemotherapy should be discontinued (mFOLFOX6 or the 5-FU (bolus) of sLV5FU2 can be omitted).

If the start of the protocol chemotherapy is delayed at least twice for a total duration of more than 28 days, it will be regarded as discontinuation of protocol chemotherapy.

(However, this may be shortened or delayed, depending on whether there is a public holiday, or in consideration of the patient's personal circumstances regarding site visits.)

Adverse events in the previous cycle	Criteria for dose hold or discontinuation				Criteria for restart or dose reduction	
	Previous cycle Grade	Bmab	FL	L-OHP	Restart Grade/ Test value	Action taken
Neutropenia (Leukopenia)	**G4**	Dose hold			**≥1,200/mm³**	Restart **5-FU, L-OHP** at one dose level lower
Febrile neutropenia	**≥G3**				**G0**	
Thrombocytopenia	**≥G3**				**G1**	
Hypertension	**G3** (excluding situations where it can be controlled with drug therapy)	Dose hold	**Give mFOLFOX6 (or sLV5FU2)**		**≤G3** If controllable	Restart **Bmab**
	**G4**	Discontinue protocol chemotherapy				
Proteinuria (on day of starting treatment)	**G3**	Dose hold	**Give mFOLFOX6 (or sLV5FU2)**		**G2**	Restart **Bmab**
	**G4**	Discontinue protocol chemotherapy				
Gastrointestinal perforation	-					
Venous thromboembolism	**≥G3**					
Arterial thromboembolism	-					
Hemorrhage	**G2** 1st	Dose hold	**Give mFOLFOX6 (or sLV5FU2)**		**G1**	Restart **Bmab**
	**G2** 2nd	Discontinue protocol chemotherapy				
	**≥G3**					
Hemoptysis	-					
Reversible posterior	-					

leukoencephalopathy					
Neuropathy	**G2, 3**	**Give sLV5FU2+Bmab**	Dose hold	**G1**	Restart **L-OHP** at one dose level lower
	**G4**	Discontinue protocol chemotherapy			
Allergic reaction	**≥G3**				
Other non-hematologic toxicity	**≥G3**	Dose hold		**G1**	Restart **5-FU, L-OHP** at one dose level lower
Non-hematologic toxicity	**G4**	Discontinue protocol chemotherapy			

Bmab=bevacizumab, FL=sLV5FU2, L-OHP=oxaliplatin

6.6.3. Group B: Criteria for Dose Reduction, Hold, and Discontinuation of mFOLFOX6 plus Cetuximab

For the first cycle, check that the criteria for starting a treatment cycle (see 6.6.4) are met, and start from dose level 0.

From the next cycle onwards, check that the criteria for starting a treatment cycle (see 6.6.4) are met and reduce the dose one level, as shown below, in response to the adverse event that occurred in the previous cycle.

However, if the dose has already been reduced because of oxaliplatin-induced neuropathy, the dose of only the drug without a previous dose reduction can be reduced one level.

If it can be concluded that the adverse event is not related to Cmab, it is possible to continue Cmab alone.

At the discretion of the subinvestigator, the starting dose for the next cycle can be reduced two levels at one time after the occurrence of clinically significant adverse events.

Dose reduction up to two levels (dose reduction to level -2 or discontinuation) may be permitted at the discretion of the subinvestigator for each of Cmab, FL, and L-OHP, and if a three-level dose reduction is deemed necessary, the protocol chemotherapy should be discontinued (mFOLFOX6 or the 5-FU (bolus) of sLV5FU2 can be omitted).

If the start of the protocol chemotherapy is delayed at least twice for a total duration of more than 28 days, it will be regarded as discontinuation of protocol chemotherapy. (However, this may be shortened or delayed, depending on whether there is a public holiday, or in consideration of the patient's personal circumstances regarding site visits.)

Adverse events in the previous cycle	Criteria for dose hold or discontinuation					Criteria for dose reduction or restart	
	Previous cycle Grade		Cmab	FL	L-OHP	Restart Grade/ Test value	Action taken
Neutropenia (Leukopenia)	**G4**		Continuation possible*	Dose hold		**≥1,200/mm³**	Restart **5-FU, L-OHP** at one dose level lower
Febrile neutropenia	**≥G3**					**G0**	
Thrombocytopenia	**≥G3**					**G1**	
Infusion reaction	**≥G3**		Discontinue	**Continue treatment with mFOLFOX6 (or sLV5FU2)**			
Skin symptoms	**G3**	1st	Dose hold	**Give mFOLFOX6 (or sLV5FU2)**		**G2**	Restart **Cmab**
		2nd, 3rd					Restart Cmab at one dose level lower
		4th	Discontinue				
Hypomagnesemia**	**≥G3**		Dose hold	**Give mFOLFOX6 (or sLV5FU2)**		**G2**	Restart Cmab at one dose level lower
Neuropathy	**G2, 3**		**Give sLV5FU2+Cmab**		Dose hold	**G1**	Restart **L-OHP** at one dose level lower
	**G4**		Discontinue protocol chemotherapy				
Allergic reaction	**≥G3**						
Reversible posterior leukoencephalopathy	-						
Other non-hematologic toxicity	**≥G3**		Continuation possible*	Dose hold		**G1**	Restart **5-FU, L-OHP** at one dose level lower
Non-hematologic toxicity	**G4**		Continuation possible*	Discontinue protocol chemotherapy			

Cmab=cetuximab, FL=sLV5FU2, L-OHP=oxaliplatin

*: If it can be concluded that the adverse event is not related to Cmab, Cmab may be continued.

*: Hypomagnesemia: See the grading chart below.

	Grade				
	1	2	3	4	5
Hypomagnesemia	<LLN-1.2 mg/dl<br><LLN-0.5 mmol/l	<1.2-0.9 mg/dl<br><0.5-0.4 mmol/l	<0.9-0.7 mg/dl<br><0.4-0.3 mmol/l	0.7 mg/dl<br>0.3 mmol/l<br>Life threatening	Death

6.6.4. Criteria for Starting a Treatment Cycle

Check that the following criteria are met on or the day before the planned day of administration, and start the protocol chemotherapy (as a general rule, the dose should be administered within two weeks after the enrollment date (the same day of the week is permissible)).

If the criteria for starting the cycle are not met, delay until they are met.

***: In Group B, if it can be concluded that the adverse event is not related to Cmab, Cmab may be continued alone.**

If the start of the protocol chemotherapy is delayed at least twice for a total duration of more than 28 days, it will be regarded as discontinuation of protocol chemotherapy.

(However, this may be shortened or delayed, depending on whether there is a public holiday, or in consideration of the patient's personal circumstances regarding site visits.)

Variable	Criteria for starting drug treatment	
Neutrophils	≥1,200/mm^3	
Platelets	≥7.5×10^4/mm^3	
AST, ALT	≤200 IU/L	
Hemoglobin	≥8.0 g/dL	
Serum total bilirubin	≤2.0 mg/dL	
Serum creatinine	≤1.5 mg/dL	
Infection	No fever with a temperature of ≥38°C, suggestive of infection	
Neuropathy	≤Grade 1*	Deep tendon reflex is absent or abnormal sensation (including pain) is present, but there is no dysfunction
Proteinuria	≤2+[NB)]	
Hemorrhage	No hemorrhage<br>(For mucosal hemorrhage, ≤Grade 1)	There is no hemorrhage (for mucosal hemorrhage, mild and requires no treatment)
Skin symptoms	≤Grade 2*	
Hypomagnesemia	≤Grade 2	
Other	Adverse events that do not meet the above criteria for which dosing may be delayed, if the subinvestigator deems it necessary in light of the nature and intensity of the adverse event.	

* For Grade 2 or 3 neuropathy only, oxaliplatin alone is held and the next cycle can be started.

** The assessment of skin symptoms should be graded for adverse events corresponding to acne, rash/desquamation, rash, dry skin, paronychia, pruritus, skin reaction, nail disorder, alopecia, cheilitis, skin disorder, urticaria, hand-foot syndrome, dermatitis exfoliative, dermatitis acneiform, skin fissures, skin toxicity, hair disorder, and hirsutism.

NB: See the chart below for conversion of proteinuria grade, and qualitative and quantitative values.

	Grade					
	1		2		3	
Proteinuria	Qualitative	1+	Qualitative	2+	Qualitative	-
	Quantitative	<1.0 g/24 h	Quantitative	1.0-3.5 g/24 h (adult)	Quantitative	≥3.5 g/24 h (adult)

*: Hypomagnesemia: See the grading chart below.

	Grade				
	1	2	3	4	5
Hypomagnesemia	<LLN-1.2 mg/dl<br><LLN-0.5 mmol/l	<1.2-0.9 mg/dl<br><0.5-0.4 mmol/l	<0.9-0.7 mg/dl<br><0.4-0.3 mmol/l	<0.7 mg/dl<br><0.3 mmol/l<br>Life threatening	Death

6.6.5. Consultations on Protocol Therapy

Contact the following for questions about changing the treatment.

Study Secretariat Personnel: Responsible person Yasunori Emi
Satomi Abe
abesato@surg2.med.kyushu-u.ac.jp
Department of Surgery and Science, Graduate School of Medical Sciences, Kyushu University
Department of Surgery II
3-1-1 Maidashi, Higashi-ku, Fukuoka, Fukuoka Prefecture 812-8582. Tel: 092-642-5466

6.7. Concomitant and Supportive Therapies

6.7.1. Recommended/Non-recommended Concomitant and Supportive Therapies

The following concomitant and supportive therapies are recommended. Failure to provide such therapies does not constitute a protocol violation.

1. G-CSF products

   G-CSF is to be administered in accordance with the health insurance indications, and is not to be given for prophylactic purposes

2. Premedication, including prophylactic use of antiemetics

   Premedication using $5HT_3$ receptor antagonists, NK1 receptor antagonists, and steroids and antihistamines, may be given by the method usually employed at each individual study site

6.7.2. Permitted Concomitant and Supportive Therapies

Drugs for treating adverse events may be used concomitantly at the discretion of the subinvestigator. Symptomatic therapy that continues from before the start of the study is also permitted.

6.7.3. Prohibited Concomitant and Supportive Therapies

Prohibited concomitant drugs and therapies: The following drugs and treatments, which could potentially affect the safety, pharmacokinetics, or efficacy evaluations of bevacizumab plus concomitant chemotherapy, or cetuximab plus concomitant chemotherapy, are prohibited.

1. Anticancer treatments
   1) Other chemotherapies
   2) Hormone therapy
   3) Immunotherapy (BRM)
   4) Other antibody therapies

5) Radiotherapy
6) Thermotherapy
7) Other

2. The prophylactic use of drugs such as the following, administered to prevent the onset of adverse events during the study period, is permitted: $5HT_3$ receptor antagonists, NK1 receptor antagonists, steroids, antihistamines, Ca, and Mg.
3. Investigational products and unapproved drugs

6.8. Ongoing Treatment

No ongoing treatment will be specified. However, information on the details of any ongoing treatment will be collected at follow-up. For ongoing treatment, it is also possible to cross over opposing groups (Group B after Group A, or Group A after Group B). After the protocol surgery is performed and the adjuvant chemotherapy is discontinued or completed on schedule (discontinuation of protocol treatment), patients will be monitored without treatment until recurrence. After recurrence, not precluding that the same post-recurrence treatment as the protocol treatment will be selected, in this case, it will be judged that the same treatment has been selected as ongoing treatment.

After the discontinuation criteria due to toxicity have been met, and an assessment of "protocol treatment discontinuation" has been made because of patient refusal, continuing a regimen that is the same as the protocol treatment as "ongoing treatment" is not recommended. If "the discontinuation criteria are met but the same treatment is to be continued at the subinvestigator's discretion and the patient's wishes", the classification "protocol treatment continued after deviation from discontinuation criteria" will be used, rather than "protocol treatment discontinued → ongoing treatment". If the discontinuation criteria for the protocol treatment are met, but it is concluded that it is clinically appropriate to "continue the protocol treatment", the study secretariat should be consulted. With the agreement of the study secretariat and the subinvestigator, decide whether the assessment should be "protocol treatment discontinued → treated as ongoing treatment" or "deviated and protocol treatment continued". If an assessment of "deviated and protocol treatment continued" occurs frequently, there is a possibility that the discontinuation criteria for protocol treatment are clinically inappropriate. Therefore, the study secretariat will consult with the study coordinating committee, and decide whether to revise the discontinuation criteria for protocol treatment.

7. Expected Adverse Events

7.1. Expected Adverse Drug Reactions

Adverse events that are expected to occur in use of each of the drugs are described concisely in this section. See the attached tables for the most recent versions of the package inserts for each drug.

7.1.1. Bevacizumab: Avastin for IV Infusion 100 mg/4 ml, 400 mg/16 ml

1. Main adverse drug reactions (studies in Japan)
   1. Bevacizumab plus FOLFOX4 therapy (n=14)
      In total, 207 events were reported in all 14 subjects (100%). The main adverse drug reactions included neutrophil count decreased, reported in 12 subjects (85.7%), white blood cell count decreased in 11 subjects (78.6%), nausea in 11 subjects (78.6%), anorexia in 11 subjects (78.6%), and neurotoxicity in 10 subjects (71.4%). (At approval)
   2. Bevacizumab plus 5-FU/l-LV
      In total, 186 events were reported in 17 subjects (94.4%). The main adverse drug reactions included nausea, reported in 8 subjects (44.4%), epistaxis in 8 subjects (44.4%), diarrhea in 7 subjects (38.9%), hypertension in 6 subjects (33.3%), vomiting in 6 subjects (33.3%), and stomatitis in 6 subjects (33.3%).
2. Clinically significant adverse drug reactions

Shock, anaphylactoid symptoms or infusion reaction (urticaria, dyspnea, lip edema, pharyngeal edema, etc.), gastrointestinal perforation, protracted wound healing, hemorrhage (tumor-related hemorrhage including that from tumor metastatic sites (gastrointestinal tract bleeding (hematemesis, melena), pulmonary hemorrhage (hemoptysis), cerebral hemorrhage), and mucosal hemorrhage (epistaxis, gingival bleeding, vaginal bleeding)), thromboembolism (cerebrovascular accident, transient ischemic attack, myocardial infarction, angina pectoris, cerebral ischemia, cerebral infarction, and other arterial thromboembolisms, and deep vein thrombosis, pulmonary embolism, and other venous thromboembolisms), hypertensive encephalopathy, and hypertensive crisis.

7.1.2. Cetuximab: Erbitux Injection 100 mg

1. Main adverse drug reactions (studies in Japan)

   Among 39 subjects in the safety evaluation set in a Japanese Phase II study in coadministration with irinotecan hydrochloride hydrate in patients with EGFR-expressing colorectal cancer, the main adverse drug reactions were acne (87.2%), rash (61.5%), anorexia (56.4%), dry skin (51.3%), paronychia (51.3%), diarrhea (51.3%), stomatitis (51.3%), hypomagnesemia (51.3%), pruritus (43.6%), nausea (43.6%), fatigue (43.6%), and lymphocyte count decreased (30.8%) (at approval).

2. Clinically significant adverse drug reactions

   Severe infusion reaction, severe skin symptoms, interstitial lung disease, cardiac failure, severe diarrhea

7.1.3. Oxaliplatin: Elplat Infusion Solution 50 mg, 100 mg

1. Main Adverse Reactions

   (In FOLFOX4 therapy, a total of 618 patients in a Phase II study outside Japan, and in monotherapy in Phase I and Phase II studies in Japan, and in combination therapy in a Phase I/II study in Japan)

   Hematologic toxicities: white blood cell count decreased, neutrophil count decreased, hemoglobin decreased (anemia), and platelet count decreased; gastrointestinal toxicities: diarrhea, nausea, vomiting, anorexia, stomatitis, constipation, and abdominal pain; hepatic symptoms: AST (GOT) increased, ALT (GPT) increased, total bilirubin increased; psychoneurological symptoms: peripheral neurological symptoms; other: fatigue, cough and alopecia

2. Clinically significant adverse drug reactions

   Peripheral neurological symptoms, shock or anaphylactoid symptoms, interstitial pneumonia or pulmonary fibrosis, myelosuppression, hemolytic uremic syndrome, visual field defect, visual field disorder, optic neuritis, or visual acuity reduced, thromboembolism, ventricular arrhythmia or myocardial infarction, hepatic vein occlusion, and renal failure acute

7.1.4. Fluorouracil: 5-FU Injection 250 Kyowa, etc.

1. Main adverse drug reactions

   At approval and in surveillance of the incidence of adverse drug reactions up to February 1970, the main adverse drug reactions in 1,936 patients included the following: 295 events (15.2%) of anorexia, 239 events (12.3%) of diarrhea or loose stools, 172 events (8.9%) of generalized malaise, 159 events (8.2%) of nausea or vomiting, 153 events (7.9%) of leukopenia, 129 events (6.7%) of stomatitis, 92 events (4.8%) of pigmentation, and 74 events (3.8%) of alopecia.

2. Clinically significant adverse drug reactions

   Dehydration symptoms, hemorrhagic enteritis, ischemic enteritis, necrotizing enteritis, and other serious forms of enteritis, pancytopenia, leukopenia, neutropenia, anemia, thrombocytopenia, and other forms of myelosuppression, shock or anaphylactoid symptoms, leukoencephalopathy, congestive heart failure, myocardial infarction, rest angina, acute renal failure, interstitial pneumonia, impaired liver function, jaundice,

hepatic failure, gastrointestinal ulcer, severe stomatitis, acute pancreatitis, hyperammonemia with consciousness disturbance, hepatobiliary disorder (cholecystitis, bile duct necrosis, liver parenchymal injury, etc.), hand-foot syndrome, olfactory disturbance, loss of taste, fulminant hepatitis and other severe liver disorders, liver cirrhosis, ventricular tachycardia, nephrotic syndrome, oculomucocutaneous syndrome (Stevens-Johnson syndrome), toxic epidermal necrolysis (Lyell syndrome), or hemolytic anemia.

Phenytoin (brand names: Aleviatin, Hydantol, Phenytoin Powder, Phenytoin N), dyslalia, ataxia, disturbance of consciousness, and other forms of phenytoin intoxication may develop. (The mechanism is unknown, but fluorouracil may increase the blood concentration of phenytoin.)

Warfarin potassium: Because fluorouracil may enhance the activity of warfarin potassium, changes in coagulation function should be carefully monitored. (The mechanism is unknown.)

Other chemotherapies and radiotherapy: Because blood disorders, gastrointestinal tract disorders, and other adverse drug reactions may be enhanced, patient condition must be carefully monitored. If any abnormal findings occur, appropriate measures should be taken, such as reducing the dose or discontinuing the drug. (Adverse drug reactions may be mutually enhanced.)

7.1.5. Levofolinate calcium: Isovorin Injection 25 mg, 100 mg

1. Main adverse drug reactions

Among 336 patients for whom data on adverse drug reactions in treatment with levofolinate plus fluorouracil were collected, adverse drug reactions were reported in 297 patients (88.4%). The main adverse drug reactions were diarrhea, reported in 160 patients (47.6%), anorexia in 160 patients (47.6%), nausea and vomiting in 155 patients (46.1%), stomatitis in 69 patients (20.5%), and pyrexia in 64 patients (19.0%). The Grade 3 or higher adverse drug reactions were diarrhea, reported in 47 patients (14.0%), anorexia in 45 patients (13.4%), nausea and vomiting in 27 patients (8.0%), pyrexia in 5 patients (1.5%), and stomatitis in 3 patients (0.9%). The main changes in laboratory test values were white blood cell count decreased in 200 (60.7%) of 336 patients, hemoglobin decreased in 136 (40.5%) of 336 patients, total protein decreased in 48 (14.5%) of 332 patients, and platelet count decreased in 46 (13.7%) of 336 patients. The test parameters with Grade 3 or higher abnormal values were white blood cell count decreased, reported in 59 patients (17.6%), hemoglobin decreased in 30 patients (8.9%), and platelet count decreased in 8 patients (2.4%).

2. Clinically significant adverse drug reactions

Severe diarrhea, serious enteritis, myelosuppression, shock or anaphylactoid symptoms, leukoencephalopathy or psychoneurological disorders, cardiac failure congestive, myocardial infarction, rest angina, impaired hepatic function or jaundice, renal failure acute, interstitial pneumonia, gastrointestinal tract ulcer, serious stomatitis, hand-foot syndrome, disseminated intravascular coagulation (DIC), loss of smell, hyperammonemia, acute pancreatitis, fulminant hepatitis, liver cirrhosis, ventricular tachycardia, nephrotic syndrome, oculomucocutaneous syndrome (Stevens-Johnson syndrome), toxic epidermal necrolysis (Lyell syndrome), and hemolytic anemia

7.2. Evaluation of Adverse Events and Adverse Reactions

The Japanese translation (JCOG version) of the NCI-Common Terminology Criteria for Adverse Events v4.0 (CTCAE v4.0) will be used for evaluating adverse events and adverse reactions.

Adverse events will be evaluated using the grade closest to the definitions for Grades 0 to 4. If a specific intervention is described for the grade, the grading should reflect the clinical necessity for such intervention.

According to the original CTCAE, any adverse event that caused a treatment-related death was to be assessed as Grade 5; however, Grade 4, rather than Grade 5, will be used in the notations on charts in this study, and the efficacy and safety evaluation committee will review the relevant expedited notification of an adverse event and decide whether to classify the event as Grade 5. Discussion of the causal relationship between the death and the adverse event

for a treatment-related death should be included in the comments on the adverse event.

For all adverse events that occur in this clinical study, the reporting requirements in the Adverse Events column should be completed.

Records of all adverse events, including their grades, should be kept in the medical charts. These will be checked in on-site monitoring activities.

8. Schedule for Evaluations and Laboratory Tests

8.1. Pre-enrollment Evaluations

8.1.1. Patient Demographic and Clinical Characteristics

Investigate or check the following characteristics before starting administration.

1. Patient ID No., sex, age (at enrollment), date of birth, date of informed consent, height, weight, previous and current diseases (names of previous and current diseases within one year prior to the date of enrollment), usual oral medications, history of surgery, history of radiotherapy
2. History of surgery for the primary lesion (date of surgery, location of the lesion, depth of tumor invasion, degree of lymph node metastasis, histological type), history of neoadjuvant or adjuvant (radio) chemotherapy (description, duration), date of diagnosis of metastasis to liver (for deciding whether synchronous or metachronous)
3. If no primary surgery has been performed, the date of biopsy and histological type
4. Date and results of *KRAS* gene analysis
5. Tests for oncogenes other than *KRAS* and other factors (e.g., *BRAF*)

8.1.2. Lesion Findings

The radiology review required for PFS, the primary endpoint, is to be performed within 28 days before enrollment. Imaging data for measurable lesions should preferably be evaluated within 14 days before enrollment. For those patients in whom it is possible, baseline diagnostic imaging may be repeated before starting treatment after enrollment (in such circumstances, pre-treatment scans may be used as baseline information, rather than the pre-enrollment scans).

The minimum requirements are set out below.

1. Perform the minimum essential baseline diagnostic imaging of metastases to liver and diagnostic imaging for verifying that metastases are limited to the liver only
   As a general rule, contrast-enhanced chest CT, contrast-enhanced abdominal CT, and contrast-enhanced pelvic CT (contrast-enhanced trunk CT) with slice thickness no greater than 5 mm should be used.
   However, if contrast-enhanced CT is not possible for reasons such as allergy to the contrast agent, unenhanced whole-body CT plus liver MRI will be used. Ongoing evaluation of liver lesions will be done by MRI, and on-study evaluation by unenhanced CT only will not be permitted. Patients for whom this may be a possibility should not be enrolled, as a general rule. MRI scans should typically include gadolinium contrast enhancement.
   For cases in which obtaining CT scans during treatment proves impossible because of a reason such as contrast agent allergy, monitoring using unenhanced trunk CT is permitted, and the evaluations of such clinically unavoidable cases will be reviewed by the study coordinating committee.
2. Degree of liver metastasis, synchronous/metachronous, number of metastases to liver (diagnostic method, documentation of integrated diagnosis), maximum size of metastases to liver, liver subsegments (sites) containing metastases, curative resectability (possible, not possible, reason)

Patients with H2-3/difficult-to-resect, liver-only metastases from colorectal cancer will be enrolled in this study.

While the minimum essential diagnostic imaging was described above, other diagnostic imaging modalities that can provide important information may also be performed, such as angio-CT, liver (contrast-enhanced) MRI, abdominal US, whole body PET-CT, brain MRI/CT, cervical CT, and bone scintigraphy. The diagnostic modalities used in the clinical setting for "liver-only metastases", "number of liver metastases", and "difficult-to-resect" should all be reported, and it is essential to report concisely how the above three characteristics were diagnosed as overall findings.

Pay close attention to the fact that there is a difference between the number of liver metastases as RECIST-measurable lesions required for the evaluation of the primary endpoint PFS, and the number of liver metastases as clinically overall findings.

The submission of baseline images

To evaluate progression-free survival by independent centralized assessment, and evaluate the spleen volume index (SVI) described in section (6) and morphologic response described in section (7) 11.4 "Exploratory Endpoints", images should be submitted to the CRO for supporting the central radiology review (see 21.8).

Within one month after enrollment, images obtained during the treatment period should be submitted to the CRO for supporting the central radiology review (see 21.8).

[A separate set of procedures will be prepared and established for the imaging and submission methods.]

8.1.3. Laboratory Tests (pre-enrollment)

Perform the following tests within 14 days before enrollment (essential). Use the most recent data available before enrollment.

1. Peripheral blood counts: Neutrophil count, platelets, hemoglobin
2. Blood chemistry tests: T-bil, AST, ALT, Cr, and INR
3. Urinalysis: Urine protein - qualitative

8.1.4. Laboratory Tests (from enrollment to before starting treatment)

Use test data obtained up to the start of treatment as baseline values.

1. Peripheral blood counts: Neutrophil count, platelets, hemoglobin
2. Blood chemistry tests: T-bil, AST, ALT, serum albumin, Cr, ALP, CRP, K, Mg, Na
3. Urinalysis: Urine protein - qualitative
4. Tumor markers (CEA, CA19-9) must be tested once before starting treatment

8.1.5. Quality of Life (QoL) survey

The QoL survey is to be performed at enrollment using the Functional Assessment of Cancer Therapy, Colorectal (FACT-C) instrument[55)]. At enrollments, survey forms will be distributed to patients and their completion requested, and the completed survey forms will be mailed to the enrollment and data center (see 22.13).

[A separate set of procedures will be prepared and established for conducting QoL surveys.]

8.2. Tests and Evaluations during the Treatment Period

8.2.1. Treatment status

1. The date of administration, dose, whether or not there is a dose hold, dose reduction, or start delay and the applicable reasons, in each cycle

8.2.2. Radiology Review

The radiology review will be performed every eight weeks (±one week), counting from the date of enrollment.

The imaging procedures used for the assessment of tumor response will be the same as the diagnostic imaging methods used at enrollment.

At baseline diagnostic imaging, contrast-enhanced CT will be used, and if contrast-enhanced CT subsequently becomes impossible because of allergies to contrast agent during the treatment period, unenhanced whole-body CT will be used.

If required, brain MRI/CT or cervical CT will be performed.

Submission of radiology scans

To evaluate progression-free survival by independent centralized assessment, and evaluate spleen volume index (SVI) described in section (5) of 11.4 "Exploratory Endpoints", the images should be submitted to the CRO for supporting the central radiology review (see 21.8).

Within one month after confirmation of PD (RECIST-PD) by diagnostic imaging, images obtained during the treatment period should be submitted to the CRO for supporting the central radiology review (see 21.8).

If an assessment of PD (progression) is made by the subinvestigator in the radiology review but not in the central radiology review, a request will be made for submission of later images, if possible.

However, if later images are not available, the patient will be censored as of the date of the image which was assessed as PD (progression) by the subinvestigator's review.

[A separate set of procedures will be prepared and stipulated for the imaging and submission methods.]

8.2.3. Clinical Laboratory Tests: Perform once every two weeks (on the day before or on the day of administration).

1. Peripheral blood counts: Neutrophil count, platelets, hemoglobin
2. Blood chemistry tests: T-bil, AST, ALT, serum albumin, Cr, ALP, CRP, K, Mg, Na
3. Urinalysis: Urine protein - qualitative
4. Tumor markers (CEA, CA19-9) (to be tested at least once every eight weeks, to match the schedule for the radiology review)

8.2.4. Signs and Symptoms: Evaluate once every two weeks (on the day before or day of administration), and whenever the protocol treatment is discontinued.

For symptoms and signs, the Japanese (JCOG) translation of CTCAE v4.0 will be used for evaluating adverse events, and will be done once every 2 weeks (on the day before or day of administration). If adverse events are reported, the best available treatment will be given, and the details will be documented on the medical records and the case report form. This will not apply when the symptoms or signs are associated with worsening of the primary disease.

1. PS, body weight, gastrointestinal perforation, hemorrhage, wound healing complications, thrombosis/blood clot/embolism, hypertension, mucositis/stomatitis, anorexia, nausea, vomiting, constipation, diarrhea, fatigue, malaise, pyrexia, febrile neutropenia, infection, allergic reaction, neuropathy (sensory), skin symptoms (acne, rash/desquamation, rash, dry skin, paronychia, pruritus, skin reaction, nail disorder, alopecia, cheilitis, skin disorder, urticaria, hand-foot syndrome, dermatitis exfoliative, dermatitis acneiform, skin fissures, skin toxicity, hair disorder, hirsutism)

8.2.5. Quality of Life (QoL) Survey

The QoL survey will be carried out using the FACT-C (Functional Assessment of Cancer Therapy, Colorectal) instrument[55)], at (i) registration, (ii) 16 weeks, (iii) 32 weeks and (iv) 48 weeks. At enrollment, survey forms will be distributed to patients for completion, and the completed survey forms will be mailed to the data center (see 21.12).

[A separate set of procedures will be prepared and established for conducting QoL surveys.]

8.3. Surgical Tests and Evaluations

8.3.1. Tests and evaluations before liver resection

The feasibility of liver resection will be evaluated for all patients, and the results reported for all patients.

Patients who have actually undergone liver resection will be evaluated before surgery. Status after the end of the basic eight cycles for patients who do not undergo the protocol liver resection

1. Date of assessment of liver resectability (date of imaging procedure)
2. Assessment of liver resectability
3. Contrast-enhanced CT or MRI of the chest, abdomen, and pelvis (slice thickness no more than 5 mm)
4. The degree of liver metastasis, number of liver metastases, the maximum size of liver metastases, and the subsegments (sites) containing liver metastases
   (According to information obtained before surgery)
5. Peripheral blood counts: White blood cell count (neutrophil count), platelets, hemoglobin
6. Blood chemistry tests: T-bil, AST, ALT, serum albumin, Cr
7. ICG-R15 value (essential)
8. Presence of ascites
9. Informed consent: If it is concluded by the subinvestigator that the liver is resectable and informed consent cannot be obtained from the patient in person, the assessment for (2) will be documented as "Resectable" and for (9) as No consent.

8.3.2. Surgical Findings of Liver Resection

Date of surgery, operative time, surgical technique (including exploratory laparotomy), use of vascular clamping, number of liver metastases (surgical findings, pathologic findings), number of liver resections, liver subsegments resected, hepatic hilar lymph node metastases, residual tumor R (pathologic diagnosis), volume of blood loss (in grams), blood transfusion volume (in grams), concomitant radiofrequency ablation (residual tumor absent + RFA, residual tumor present + RFA), concomitant portal embolization, date of discharge, other concomitant surgery (cholecystectomy, resection of hepatic hilar lymph node metastases, resection of other organs)

8.3.3. Evaluations after Liver Resection

1. Intraoperative and postoperative complications
2. To perform the following pathologic reviews specified in 11.4. "Exploratory Endpoints", specimens should be submitted to the data center (see 21.12): (1) tumor regression grade (TRG), (2) modified tumor regression grade (mTRG), (3) dangerous halo (HALO), (4) sinusoidal obstruction syndrome (SOS), and (5) other.
   Within one month after discontinuation or completion of the protocol treatment, prepared specimens should be submitted to the data center (see 21.12).

[A separate set of procedures will be prepared and established for the preparation and submission of pathologic specimens.]

8.3.4. Surgical Findings of Primary Resection

Date of surgery, operative time, surgical technique (including exploratory laparotomy), site, degree of lymph node dissection, residual tumor R (pathologic diagnosis), volume of blood loss (in grams), blood transfusion volume (in grams), date of discharge

8.4. Tests and Evaluations at the Completion or Discontinuation of Treatment

8.4.1. Outcome

1. Date of treatment discontinuation/completion
2. Reason for treatment discontinuation/completion
3. Ongoing treatment
   Chemotherapy, liver resection (including ablation) performed for ongoing treatment, hepatic arterial infusion, or other primary or metastasis resection procedures, radiotherapy
4. Progression/recurrence (RECIST-PD, clinical progression)
5. Date of confirmation of progression/recurrence
6. Site of recurrence
7. If progression is not evident, response will be assessed every eight weeks (±one week) by the diagnostic imaging method used at enrollment. (Counting from the day of enrollment)
8. For patients with R0 or R1 liver resections, the response will be assessed until confirmation of new lesions (including recurrence), by the diagnostic imaging method used at enrollment. As a general rule, this will be done every 12 weeks (±one week) in the first year, every 16 weeks (±one week) in the second year, and every 24 weeks (±one week) in the third year after resection, and for patients with R2 resections, every eight weeks (±one week). (Counting from the day of resection)
9. Outcome of death or survival
10. Date of confirmation of death or survival
11. In the event of death, the reason for death and the causal relationship with the treatment

8.5. Variables to be Investigated as Required

1. If interstitial pneumonia, dyspnea, or similar disease is evident: Arterial blood gases - PaO2, chest X-rays
2. If arrhythmia or other symptoms suggestive of cardiotoxicity are evident: Resting 12-lead ECG

8.6. Study Calendar

Study Calendar

<table>
<tr><td colspan="2" rowspan="2">Time windows for site visits</td><td colspan="2">Before starting treatment</td><td>Cycle 1 (Days 1 to 14)</td><td>Cycle 2 (Days 15 to 29)</td><td>Cycle 3 onwards</td><td>Before resection</td><td>After resection</td><td>End of study or discontinuation</td></tr>
<tr><td>Within 28 days</td><td>Within 14 days</td><td>Day before or Day 1</td><td>Day before or on the day</td><td>Day before or on the day</td><td>From the last chemotherapy until surgery</td><td>NB.</td><td></td></tr>
<tr><td colspan="2">Plasma for ancillary research</td><td></td><td>○*</td><td></td><td></td><td></td><td></td><td></td><td></td></tr>
<tr><td colspan="2">Radiology review</td><td>○</td><td></td><td colspan="3" rowspan="2">Counting from the day of enrollment, every 8 weeks (±1 week)</td><td>○</td><td>○</td><td>○NB</td></tr>
<tr><td colspan="2">Tumor markers: CEA, CA19-9</td><td>○</td><td></td><td>○</td><td>○</td><td>○</td></tr>
<tr><td colspan="2">Laboratory tests: Peripheral blood count</td><td>○</td><td></td><td>○</td><td>○</td><td>○</td><td>○</td><td>○</td><td></td></tr>
<tr><td colspan="2">Laboratory tests: Blood chemistry tests</td><td>○</td><td></td><td>○</td><td>○</td><td>○</td><td>○</td><td>○</td><td></td></tr>
<tr><td colspan="2">Signs and symptoms</td><td></td><td></td><td>○</td><td>○</td><td>○</td><td>○</td><td>○</td><td>○</td></tr>
<tr><td colspan="2">Treatment status</td><td></td><td></td><td>○</td><td>○</td><td>○</td><td></td><td>○</td><td></td></tr>
<tr><td>Before liver resection</td><td>ICG15</td><td></td><td></td><td></td><td></td><td></td><td>○</td><td></td><td></td></tr>
<tr><td rowspan="2">After liver resection</td><td>Pathology review**</td><td></td><td></td><td></td><td></td><td></td><td></td><td>○</td><td></td></tr>
<tr><td>Postoperative complications***</td><td></td><td></td><td></td><td></td><td></td><td></td><td>○</td><td></td></tr>
<tr><td colspan="2">QoL survey</td><td colspan="8">Four times: (i) At enrollment; (ii) at 16 weeks; (iii) at 32 weeks; and at 48 weeks</td></tr>
<tr><td colspan="2">Arterial blood gases: PaO2</td><td colspan="8">Whenever dyspnea is present</td></tr>
<tr><td colspan="2">Chest X-rays</td><td colspan="8">Whenever dyspnea is present</td></tr>
<tr><td colspan="2">Resting 12-lead ECG</td><td colspan="8">Whenever arrhythmia is suspected</td></tr>
</table>

NB: Until progression is confirmed, every eight weeks (±one week) as a rule (counting from the day of enrollment). For patients with R0 or R1 liver resection, response will be assessed until confirmation of new lesions (including recurrence), by the diagnostic imaging method used at enrollment. As a general rule, this will be done every 12 weeks (±one week) in the first year, every 16 weeks (±one week) in the second year, and every 24 weeks (±one week) in the third year after resection, and for patients with R2 resections, every eight weeks (±one week).

*: For quantitative measurement of pVEGF-A, (i) Before starting treatment, (ii) Before completing eight cycles, and (iii) at PD or recurrence.

(For patients who underwent liver resection, before liver resection, and after completion of 12 cycles.)

**: At the time of liver resection, assess TRG, mTRG, HALO, SOS, and perform other pathologic evaluations.

***: After liver resection, monitor postoperative complications until improvement (including after change of hospital).

9. Data Collection

9.1. EDC System

In this study, an electronic data capturing (EDC) system will be used for the preparation and submission of case report forms. Prior to using the EDC system, and after approval by the study site's ethics committee or IRB, the subinvestigator will obtain a personal user ID and password from the data center, after which they will be able to prepare and submit case report forms.

9.2. Preparation of Case Report Forms (CRFs)

The preparation, submission, amendment, and inspection of case report forms will all be achieved via the EDC system. Subinvestigators will enter data by the specified deadlines, in accordance with the data entry manual.

10. Reporting of Adverse Events

10.1. Adverse Events

All adverse events that occur during the study period will be subject to evaluation.

On the occurrence of an adverse event, the subinvestigator will enter the details into the EDC at the corresponding time, and report to the data center at the times of submission specified on each survey form. As far as possible, the outcome of the particular adverse event will be followed during the study period, and entered in the EDC.

10.2. Serious Adverse Events

On the occurrence of a serious adverse event, the investigator will promptly file a report in accordance with the reporting procedure. Reporting to the Minister of Health, Labour and Welfare on adverse drug reactions pursuant to the Pharmaceutical Affairs Law, reporting to the head of each study site or the regulatory authorities on serious adverse events pursuant to "Ethical Guidelines for Clinical Studies", and notification of adverse drug reactions from study sites to companies will be carried out appropriately at the responsibility of the investigator, in accordance with each site's regulations.

10.2.1. Expedited Reporting

Serious adverse events that occur in this study will be subject to expedited reporting.

1. Seriousness
   1) Death
   2) Disability
   3) Is potentially life-threatening
   4) Is potentially disabling
   5) Inpatient hospitalization or prolongation of existing hospitalization for treatment of the adverse event.
   6) Seriousness corresponding to (i) through (v) above.
   7) Is a congenital anomaly/birth defect
2. On the occurrence of such an adverse event, the subinvestigator will promptly inform the investigator. If contact cannot be made with the investigator, the subinvestigator must act in place of the investigator.
3. If such an event is verified, the investigator will promptly report to the head of the study site. After becoming aware of the particular adverse event, the investigator should complete the required entries in the EDC within 72 hours, and send an initial report to the principal investigator and study secretariat.
4. Details of the clinical course should be entered into the EDC and a supplementary report sent to the principal investigator and study secretariat.
5. Upon receiving a report from the principal investigator of a serious adverse event occurring at another site, the investigator should report to the head of their study site of a "serious adverse event concerned with this clinical study".

10.3. Responsibilities of the Principal Investigator

10.3.1. Deciding whether it is necessary to terminate the study and send an urgent notification to study sites

After receiving a urgent notification of an adverse event from the investigator, the study secretariat will report to and consult with the principal investigator, assessing the urgency, importance, and effect of the report's contents and, as required, suspend enrollment temporarily (notify the data center and all participating study sites), and take steps such as sending expedited communications of known issues to participating sites. Telephone communications with the data center are permissible, depending on the degree of urgency of the communication, and written communications (fax, mail, or email) should also be sent as quickly as possible afterwards.

10.3.2. Reporting to the efficacy and safety evaluation committee

If the principal investigator concludes that an expedited notification is required, he will promptly report in writing to the efficacy and safety evaluation committee after becoming aware of the adverse event, and seek the views of the principal investigator on the adverse event and request a review of the appropriateness of the response to the adverse event.

10.3.3. Notifications to Study Site Investigators

If the principal investigator has reported to the efficacy and safety evaluation committee, the details of the review and recommendations of the efficacy and safety evaluation committee will be communicated in writing to the investigator.

If a report has not been made to the efficacy and safety evaluation committee, the principal investigator will communicate his decision in writing to the investigator that filed the report.

10.3.4. Review of Adverse Events in Periodic Monitoring

In periodic monitoring, the principal investigator will carefully review the adverse event reports in the monitoring report prepared by the data center, and check that there are no omissions in the reports from the study sites. Conversely, he will also check that all reported adverse events are listed in the periodic monitoring report. Reporting omissions will be detailed in the column of the periodic monitoring report for reporting group review results column.

10.3.5. Investigations by the Efficacy and Safety Evaluation Committee

The efficacy and safety evaluation committee will review and examine the reports, and make recommendations in writing to the principal investigator on future actions to be taken, including the appropriateness of continuing enrollments, and the need for protocol revision, and report on the evaluations, including the responses to the evaluation results to the ethics committee.

11. Assessment of Response and Definitions of Endpoints

11.1. Assessment of Tumor Response

For evaluable lesions, the objective tumor response will be assessed as a percentage response rate.

The assessment method shall follow the procedure below, in accordance with the Response Evaluation Criteria In Solid Tumours (RECIST ver. 1.1.)[56]. For further details, see the Response Evaluation Criteria In Solid Tumours (RECIST ver. 1.1.)[56].

11.1.1. Baseline Evaluation

In accordance with the pre-enrollment evaluation items listed in section 8.1, neoplastic lesions should be identified before enrollment, and each lesion classified as either a "measurable lesion" or a "non-measurable lesion".

As a general rule, contrast-enhanced chest CT, contrast-enhanced abdominal CT, or contrast-enhanced pelvic CT

(contrast-enhanced trunk CT) with slice thickness no greater than 5 mm should be used.

Unenhanced CT of the chest only may not be used.

However, if contrast-enhanced CT is not possible for reasons such as allergy to the contrast agent, unenhanced whole-body CT plus liver MRI will be used. As a general rule, MRI scans should include gadolinium contrast enhancement. Abdominal MRI should be used to evaluate metastases to liver, and patients for whom serial MRI scans cannot be evaluated will not be enrolled.

Imaging for the assessment of tumor response will be done by the same diagnostic imaging methods used at enrollment.

At baseline, contrast-enhanced CT will be used for diagnostic imaging, and if contrast-enhanced CT subsequently becomes impossible because of allergies to contrast agent during the treatment period, unenhanced whole-body CT will be used and evaluations continued, but the final evaluations will be reviewed by the study coordinating committee.

11.1.2. Definition of Measurable Lesion

Lesions that meet any of the following criteria shall be regarded as measurable lesions.

1. Lesions other than lymph node lesions (non-lymph node lesions) that meet any of the following requirements
    1) At contrast-enhanced CT or MRI with a slice thickness of no more than 5 mm, the longest diameter is ≥10 mm
    2) At contrast-enhanced CT or MRI with a slice thickness of more than 5 mm, the longest diameter is at least twice the slice thickness
    3) Lytic bone metastatic lesions with soft tissue components that meet the criteria in (i) or (ii) above
    4) Other cystic metastatic lesions without non-cystic lesions, that meet the criteria in (i) or (ii) above
2. Lymph node lesions with short axis length ≥15mm at contrast-enhanced CT or MRI with a slice thickness of no more than 5 mm

   Lesions with 10 mm≦ short axis length < 15 mm will be defined as non-target lesions, and those with short axis length < 10 mm defined as being within the normal range.
3. Clinical lesions that are measurable and can be photographed in color, with a longest diameter at least 10 mm (Superficial skin lesions, etc.)

All lesions other than those described above shall be regarded as non-measurable lesions.

However, caution should be paid to the following lesions, because they will be classified as non-measurable lesions, regardless of the imaging method or their size.

1. Bone lesions (excluding lytic lesions with measurable soft tissue components)
2. Cystic lesions (excluding (1) (iv) above))
3. Lesions with prior local treatment, such as radiotherapy
4. Leptomeningeal lesions
5. Ascites, pleural or pericardial effusion
6. Lymphangitis with skin or lung involvement
7. Palpable abdominal masses or abdominal organomegaly identified by physical exam that are not measurable by imaging techniques

11.1.3. Selection of Target Lesions and Baseline Records

At radiology review (contrast-enhanced CT or abdominal MRI), up to three of the measurable liver metastases seen at enrollment most suited to reproducible, repeated measurement, in descending order of size (hereunder, long axis length), will be selected and designated as target lesions.

For patients with synchronous liver metastases and primary lesion present at enrollment, up to two lesions that are regional lymph node metastasis-positive and have a short axis length at least 15 mm, in descending order of size, will

be selected and added as target lesions.

Patients with liver-only metastases are essential in this study, and their evaluation is important. For patients with three or more measurable (long axis length at least 10 mm) metastases to liver, the selection of three lesions as target lesions is essential (RECIST Ver. 1.1 optional provisions).

All others will be classified as non-target lesions. The site (code) of the selected target lesion, imaging method, date of imaging, long axis, and the sum of long axis lengths for all target lesions (hereunder, sum of long axes) will be recorded in case report forms.

The primary lesions in synchronous liver metastases will be classified as non-target lesions (only when there is progression sufficient to require a change in the treatment will they be assessed as PD, and a slight increase in size will not be classified as PD).

Classification of lymph nodes: Measure the short axis; lesions with short axis length of at least 15 mm will be classified as measurable lesions. However, if the lymph node short axis length is ≥10 mm and <15 mm, it will be classified as a non-target lesion, and if the length is <10 mm, it will be classified as being in the normal range.

11.1.4. Selection of Non-Target Lesions and Baseline Records

The following data for lesions that are not selected as target lesions will be recorded in case report forms: sites for all non-target lesions regardless of whether measurable or not, imaging method, and the date of imaging only (liver lesions not classified as target lesions, primary lesions and regional lymph node metastases (short axis length ≥10 mm and <15 mm)).

11.1.5. Assessment of Tumor Response

Every eight weeks(±one week) starting from the day of enrollment, target and non-target lesions will be evaluated in accordance with section 8.2 "Tests and Evaluations During the Treatment Period", using the same imaging method as that used at enrollment, and the long axis lengths of target lesions, and the disappearance or progression of non-target lesions will be recorded on case report forms.

11.1.6. Criteria for Assessment of Target Lesion Response

1. **CR: Complete Response**
   All target lesions excluding lymph nodes have disappeared, and lymph nodes have short axis reductions to <10 mm.
2. **PR: Partial Response**
   At least a 30% decrease in the sum of long axes of target lesions, taking as reference the baseline sum of long axes.
3. **PD: Progression**
   At least a 20% increase in the sum of long axes of target lesions, taking as reference the smallest sum on study, and the sum must also demonstrate an absolute increase of at least 5 mm.
4. **SD: Stable Disease**
   Neither sufficient shrinkage to qualify for PR nor sufficient increase to qualify for PD.
5. **NE: Not Evaluable**
   When imaging cannot be done for any reason, or when an assessment of CR, PR, PD, or SD cannot be made.

Sum of long axes at baseline - Sum of long axes at time of evaluation

Percent shrinkage of long ~~axes = × 100%~~

Sum of long axes at baseline

Sum of long axes at baseline - Sum of long axes at time of evaluation

Percent increase in long axes = ———————————— × 100%

Smallest sum of long axes

11.1.7. Criteria for Assessment of Non-target Lesion Response

1. **CR: Complete Response**

   Disappearance of all non-target lesions, and lymph node lesions have short axis lengths reduced to <10 mm, and all tumor markers (CA19-9, CEA) have decreased below the site's upper limit of normal.

2. **non-CR/non-PD**

   At least one non-target lesion persists, or any of the tumor markers exceeds the site's upper limit of normal.

3. **PD: Progression**

   Increase in non-target lesion (increase of 73% in volume terms)

4. **NE: Not Evaluable**

   When imaging cannot be done for any reason, or when an assessment of CR, non-CR/non-PD, or PD cannot be made.

* "Unequivocal increase" means that overall the status is PD, and that treatment must stop, and in short, signifies that there is an increase that is equivalent to that of PD in measurable lesions.

11.1.8. Appearance of New Lesions

The "appearance of new lesions" shall not affect the "target lesion response" and "non-target lesion response", and the "target lesion response" and "non-target lesion response" are to be evaluated separately.

The specified diagnostic imaging method will be used to assess lesion response at baseline, but the clinical symptoms and findings, and a definitive diagnosis of "the appearance of new lesions" using other forms of diagnostic imaging can also, as a matter of course, be used for diagnosing the appearance of new lesions.

Example: ・Brain CT or MRI is performed because of headache, and a definitive diagnosis of brain metastasis is obtained

・MRI is performed because of pain or other reason, and a definitive diagnosis of bone metastasis is obtained

・Excision biopsy is performed for a skin mass that was not present at the start of treatment, and skin metastasis is confirmed, etc.

If new lesions are not well-defined and a definitive diagnosis is not possible (for example, small size), the study secretariat should be notified. The study secretariat will seek a decision from the study coordinating committee.

Special Notes

The finding of a new lesion must be confirmed clinically, and must be unequivocal. For example, some "new" bone lesions may represent apparent progression and be simply healing or flares of pre-existing lesions. This requires particular attention when the lesions identified at baseline show a partial or complete response. For example, necrosis of a liver lesion may be reported on a CT scan report as a "new" cystic lesion, which it is not.

Furthermore, lesions that are identified in follow-up studies in organs or at sites that were not scanned at baseline are considered new lesions and indicate disease progression. An example of this is the patient who has visceral disease at baseline and while on study has a CT or MRI of the brain which reveals metastases. Such a patient's brain metastases are considered to be evidence of PD even if he/she did not have brain imaging at baseline.

11.1.9. Overall Response

The overall response will be assessed every eight weeks (±one week), starting from the day of enrollment, based on the combination of the response for target lesions and response for non-target lesions, in accordance with the table below. However, if the response for either of the target or non-target lesions is NE, the overall response will be NE.

For a response assessment of CR or PR, the "4-week duration" stipulated in the WHO criteria is not required, and the date of assessment of an overall response of CR or PR will signify the "date of assessment of CR" and "date of assessment of PR", respectively.

Target lesion response	Non-target lesion response	Appearance of new lesions	Overall Response
CR	CR	No	CR
CR	non-CR/non-PD	No	PR
CR	NE	No	PR
PR	Non-PD, or NE	No	PR
SD	Non-PD, or NE	No	SD
NE	Non-PD	No	NE
PD	Any	Any	PD
Any	PD	Any	PD
Any	Any	Yes	PD

11.1.10. Best Overall Response

With the overall response assessed in descending order as CR>PR>SD>PD>NE, the best overall response will be assessed in accordance with the criteria below, based on the overall responses in all cycles. If the definitions of multiple categories are met, the response will be classified in the best category, using the following order CR>PR>SD>PD>NE.

1. Complete response (CR)

   An overall response of CR is obtained at least twice in succession, at least four weeks (28 days) apart.

   The day on which the second overall response of CR is ascertained and the best overall response of CR is confirmed is defined as the "date of CR confirmation".

2. Partial response (PR)

   An overall response of PR or higher (CR or PR) is obtained at least twice in succession, at least four weeks (28 days) apart.

   The day on which the second overall response of PR is ascertained and the best overall response of PR is confirmed is defined as the "date of PR confirmation".

3. Stable disease (SD)

   If best overall responses of neither CR or PR have been obtained, but the overall responses up to the assessments at the end of two cycles after starting treatment are not PD, and the overall response is SD or higher at least once

4. Progressive disease (PD)

   Does not meet the criteria for best overall response CR, PR, or SD, and the overall response is PD

5. Not evaluable (NE)

First overall response	Next overall response	Next overall response	Best overall response
Either PR or CR	SD	PD	SD
Either PR or CR	SD	NE	SD
Either PR or CR	PD	—	PD
Either PR or CR	NE	NE	NE

If all overall response assessments are NE.

Either PR or CR	NE	SD	SD
SD	PD	—	PD
SD	SD	PD	SD
SD	NE	PD	PD
NE	NE	PD	PD
NE	NE	NE	NE
NE	NE	PD	PD

11.2. Definitions of Analysis Sets

Enrolled patients will be classified as shown below. The main analysis set for efficacy is defined in section 11.2.2. "Full Analysis Set" and that for safety in section 11.2.3. "Safety Analysis Set".

11.2.1. All Enrolled Patients

Among patients enrolled in accordance with the requirements of section 5.1 "Enrollment Procedure", the "all enrolled patients" set will be defined as those remaining after excluding duplicate enrollments and enrollment errors.

11.2.2. Full Analysis Set

The full analysis set is composed of "all enrolled patients", after excluding patients for whom the objective data obtained before enrollment did not meet the study eligibility criteria, patients who withdrew consent after enrollment and before starting the protocol treatment, and patients who did not receive the protocol treatment for any reason after enrollment.

11.2.3. Safety Analysis Set

Excluding those for whom the objective data obtained before enrollment did not meet the study eligibility criteria, the safety analysis set is composed of enrolled and assigned patients who started the assigned protocol treatment and for whom any data are available.

11.3. Definitions of Endpoints

11.3.1. Progression-free Survival (PFS)

Starting from the day of enrollment, the number of days to the date on which an assessment of progression is made or the date of death due to any cause, whichever occurs first.

The population for this endpoint will be the full analysis set (see 11.2.2).

Two types of PFS will be evaluated: the PFS based on the subinvestigator's radiology review (subinvestigator's PFS) and the PFS based on central radiology review (independent review committee PFS; IRC PFS). The IRC PFS is designated as the primary endpoint.

1. Progression will be defined as PD (progression) pursuant to diagnostic imaging in section 11.1.9. "Overall Response" in patients who have not undergone resection, and as recurrence in patients in whom no events occurred, and in whom resection was performed without radiologically-evident residual tumor. The date on which the imaging procedure was performed is defined as the date of progression.
   The diagnostic imaging procedure should be the same as the method used at enrollment.

2. The definition of PFS "progression" begins from clinical symptoms suggestive of metastatic progression, and this will be defined as an event if a definitive diagnosis of unequivocal new lesions was made by a diagnostic imaging method different from that at baseline or biopsy, etc., without progression or new lesions being diagnosed with the specified scans. The date of progression in this case is defined as the date of the imaging procedure that verified the diagnosis or the date of biopsy, etc. In the event of this assessment, copies of the scans, radiology reports, and the biopsy results and pathology reports are to be submitted (for central assessment).
   If it is not possible to make an assessment that a new lesion is not unequivocal but suspected, by a diagnostic imaging technique that is different from the technique used for evaluation at baseline, an inquiry should be made to the study secretariat. At this time too, copies of the scans, the radiology reports, and the biopsy results and pathology reports are to be submitted (for central assessment).
3. Survivors for whom a diagnostic imaging-based assessment of progression has not been made will be censored as of the date of the last imaging procedure on which it was confirmed from the prescribed diagnostic imaging that there was no progression.
4. Patients who discontinue the protocol treatment for reasons such as toxicity or refusal, and if another therapy is provided as ongoing treatment (any type of therapy), the event and censoring will be handled similarly. In short, they will not be censored as of the time of discontinuation of protocol treatment or the date of starting ongoing treatment. Continuation of diagnostic imaging as prescribed will be obligatory.
5. If the surgery is non-radical resection, it will be classified as an event if recurrence is confirmed from post-operative diagnostic imaging, without being classified as either an event or censoring at the time of surgery.
6. If an assessment of PD (progression) is made on the basis of diagnostic imaging during protocol treatment and surgery is performed, PD (progression) based on preoperative diagnostic imaging will be defined as an event. Postoperatively however, (including situations where another therapy is instituted as ongoing treatment), diagnostic imaging-based follow-up of PD (progression) and recurrence will be mandatory (secondary endpoint).
7. If an assessment of PD (progression) is made by the subinvestigator in the radiology review for PFS but not in the central radiology review (IRC PPFS), a request will be made for submission of a later image, if possible. However, if a later image is not available, the patient will be censored as of the date of the image from which an assessment of PD (progression) was made in the subinvestigator's review.
8. The event will be defined as occurring on the "imaging date" of the "definitive" radiology scans obtained later, rather than the imaging date on which it was "radiologically suspected".
9. The occurrences of secondary cancer, metachronous double cancer, or metachronous multiple cancer are not defined as events or censored, and the progression-free survival period is defined until observation of an event as defined above.
10. In patients for whom an event is not confirmed, lesions that cannot be confirmed via specified diagnostic imaging, and even when they can be confirmed when surgery is undertaken, will not be classified as new lesions, and above all, a judgment based on the specified diagnostic imaging technique will be used (a new lesion will be defined as such when it is recognized as a lesion via the specified diagnostic imaging after surgery). This type of lesion will not be classified as a new lesion because it is not possible to make a definitive diagnosis of whether or not it was a lesion that was present at enrollment (start of treatment).

Patients with PFS events

Primary lesion absent (enrolled after primary resection)

Event

Ongoing treatment

Discontinuation for reasons other than PD

(assessment of clinical progression/adverse events/patient circumstances, etc.)

Postoperative CTx

SD or higher, liver resection

Recurrence

Laparotomy for resection

Peritoneal metastases present

Non-regional lymph node metastases present

SD or higher (contrast-enhanced CT)

(Target lesion evaluation using MRI, etc.)

(Unequivocal new lesion on MRI, etc.)

Increase in size

* 12 or more cycles

Primary lesion present

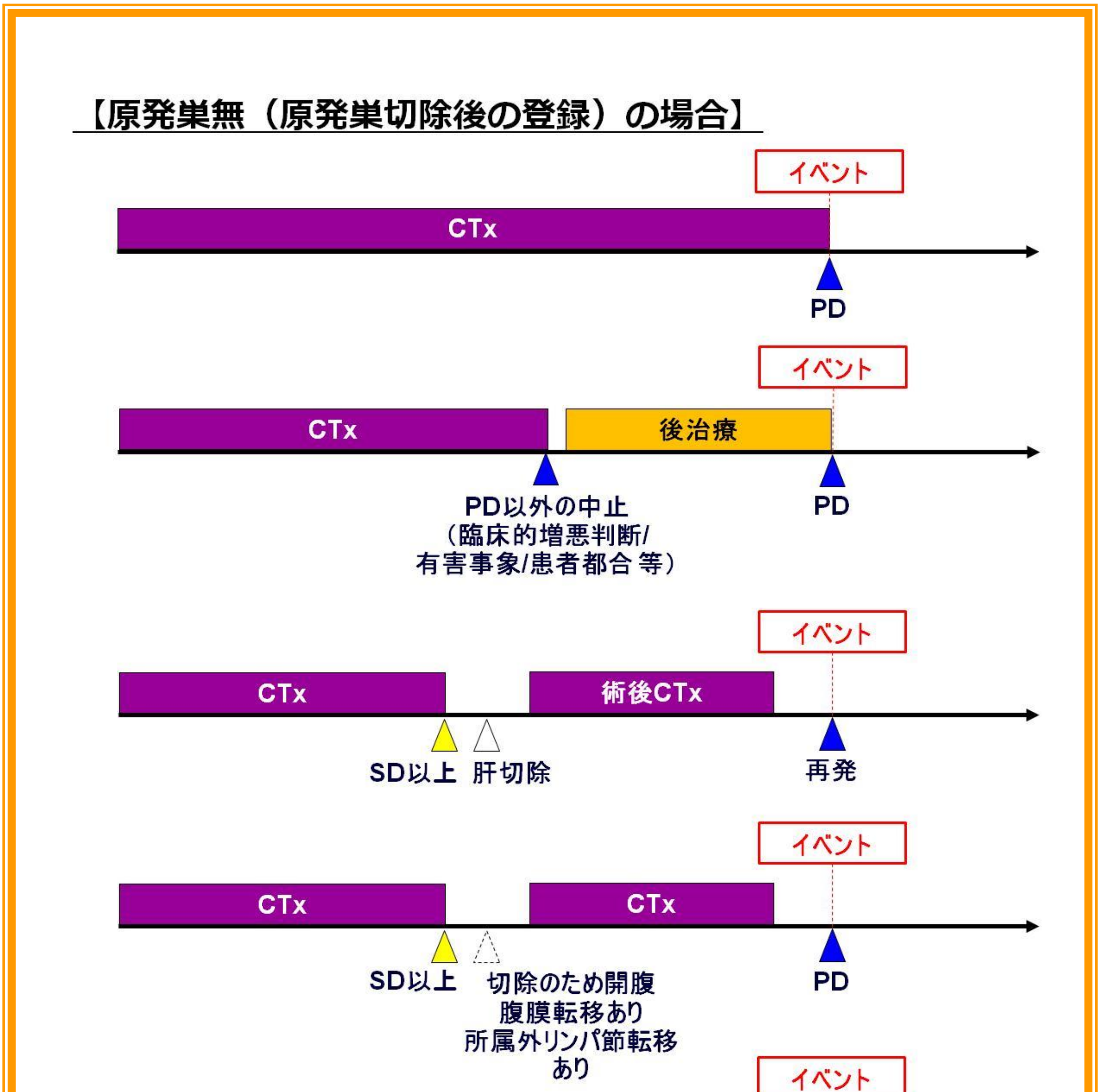

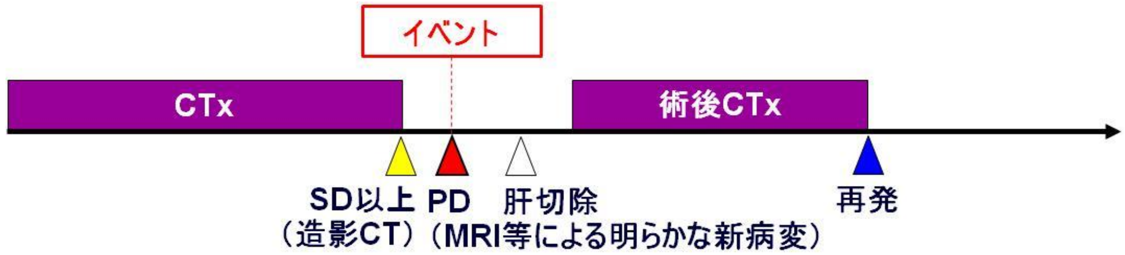

Continued

Patients with PFS events

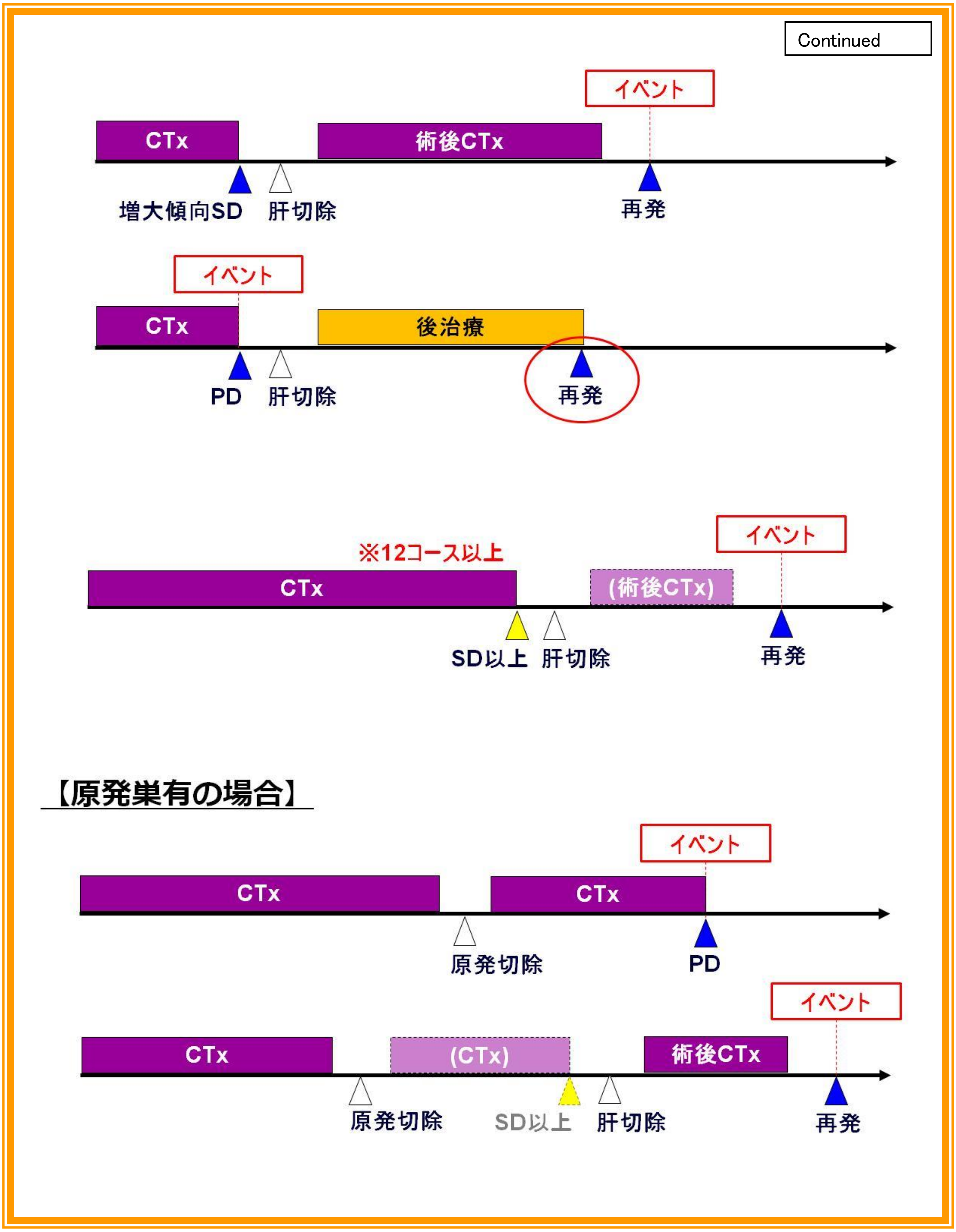

11.3.2. Response Rate (RR)

Response rates will be evaluated for the overall response (see 11.1.9) and best overall response (see 11.1.10)

The percentage of patients in the full analysis set (see 11.2.2) with measurable lesions and responses of either CR or PR for overall response (see 11.1.9) and best overall response (see 11.1.10) is defined as the response rate.

11.3.3. Tumor Shrinkage Rate at Eight Weeks

The tumor shrinkage rate at eight weeks, defined as the shrinkage rate of target lesions from baseline imaging to the first radiology review, will be evaluated. The population for this endpoint will be patients in the full analysis set (see 11.2.2) with measurable lesions.

11.3.4. Liver Resection Rate

This endpoint will be evaluated as the percentage of patients in the full analysis set (see 11.2.2) who undergo liver resection.

1. The liver resection rate will be evaluated for Groups A and B individually and combined, and by subinvestigator assessment at enrollment as resectable or unresectable.
2. The liver resection rate will also be evaluated by extent of residual tumor in each of Groups A and B.

   The final decision on whether a protocol liver resection was a two-stage or multiple-surgery procedure for "evaluation of residual tumor: R number" will be ultimately made by the study coordinating committee.

11.3.5. R0 Liver Resection Rate

This endpoint will be evaluated as the percentage of patients in the full analysis set (see 11.2.2) who undergo liver resection for whom the "evaluation of residual tumor after resection" (see 3.10) was R0.

1. The R0 liver resection rate will be evaluated for Groups A and B individually and combined, and by subinvestigator assessment at enrollment as resectable or unresectable.

11.3.6. Time to Treatment Failure (TTF) for the Protocol Chemotherapy

Starting from the date of enrollment, whichever is the earliest of the following: the date of assessment of progression, the date of death from any cause, or the date of discontinuation (completion) of protocol chemotherapy prior to the completion of protocol chemotherapy.

The population for this endpoint will be the full analysis set (see 11.2.2).

1. The date of discontinuation (completion) of protocol chemotherapy is defined as the date on which discontinuation (completion) is decided.
2. The definition of "progression" will follow that in section 11.3.1. "Progression-free Survival".
3. If liver resection is performed during protocol chemotherapy and adjuvant chemotherapy is been provided, the date of discontinuation (completion) of adjuvant chemotherapy is defined as the date on which discontinuation (completion) is decided.
4. If liver resection is performed during protocol chemotherapy and adjuvant chemotherapy is not provided, the patient will be regarded as censored as of the date of surgery.

11.3.7. Overall Survival (OS)

Starting from the date of enrollment, the number of days until death from any cause.

The population for this endpoint will be the full analysis set (see 11.2.2).

1. Survivors will be censored as of the date of final confirmation of survival (survival may be confirmed by means such as telephone, consultation for tests, consultation with another clinical service, or examination at the relevant department, and survival can be confirmed from the medical charts or other records, as a general principle).

2. Patients who are lost to follow-up will be censored as of the last date on which survival was confirmed before they were lost to follow-up.

11.3.8. Quality of Life (QoL)

The QoL survey will be carried out four times using the FACT-C (Functional Assessment of Cancer Therapy, Colorectal) instrument[55)] at (i) enrollment, (ii) 16 weeks, (iii) 32 weeks, and (iv) 48 weeks.

The population for this endpoint will be the full analysis set (see 11.2.2).

1. Changes over time in QoL will be evaluated for each of Groups A and B.

11.3.9. Incidence of Adverse Events (Adverse Reactions) (Chemotherapy-related)

With the safety analysis set (see 11.2.3) designated as the parent population for this endpoint, the frequencies (by group) will be determined for the worst grades of the adverse events (toxicities) listed below during all cycles (up to a maximum of 24 cycles), according to the Japanese translation (JCOG) of the CTCAE v4.0.

1. Peripheral blood counts: Neutrophil count, platelets, hemoglobin
2. Blood chemistry tests: T-bil, AST, ALT, serum albumin, Cr, ALP, CRP, K, Mg, Na
3. Urinalysis: Urine protein - qualitative (essential only for Group A)
4. Gastrointestinal perforation
5. Hemorrhage
6. Wound-healing complications
7. Thrombosis/blood clot/embolism
8. Hypertension
9. Mucositis/stomatitis
10. Anorexia
11. Nausea
12. Vomiting
13. Constipation
14. Diarrhea
15. Fatigue
16. Malaise
17. Pyrexia
18. Febrile neutropenia
19. Infection
20. Allergic reaction
21. Neuropathy (sensory)
22. Skin symptoms (acne, rash/desquamation, rash, dry skin, paronychia, pruritus, skin reaction, nail disorder, alopecia, cheilitis, skin disorder, urticaria, hand-foot syndrome, dermatitis exfoliative, dermatitis acneiform, skin fissures, skin toxicity, hair disorder, hirsutism)

Incidence data for adverse events (toxicities) other than those listed above will not be tabulated as a general rule, except in circumstances in which numerous specific adverse events are observed, because only Grade 3 or higher non-hematologic (blood or bone marrow categories) adverse events will be recorded in treatment charts.

11.3.10. Incidence of Adverse Events (Adverse Reactions) (Surgery-related)

With the patients who underwent liver resection in section 11.3.4 "Liver Resection Rate" designated as the parent population, the frequencies of the worst grades will be determined (by group) for the adverse events (toxicities) listed below, for intraoperative complications using the JCOG Intraoperative and Postoperative Complication Criteria prepared by the JCOG Surgical Committee, and for postoperative complications using the JCOG Postoperative

Complication Criteria according to the Clavien-Dindo classification.

1. Hepatobiliary
2. Gastrointestinal
3. Hemorrhage
4. Infection

11.3.11. Incidence of Serious Adverse Events

The percentage of patients in the safety analysis set (see 11.2.3) with at least one of the following serious adverse events will be defined as the incidence of serious adverse events.

1. All deaths during the protocol treatment period or within 30 days after the last day of chemotherapy (the cause of death does not have to be causally related to the treatment)
2. Deaths that occur after 31 days from the last date of chemotherapy, but for which a causal relationship with the treatment cannot be ruled out.
3. Grade 4 non-hematologic toxicities

11.4. Exploratory Endpoints

1. Tumor regression grade (TRG)
   With the patients who underwent liver resection defined in section 11.3.4 "Liver Resection Rate" designated as the parent population, the relationships between the percentage of each grade calculated in accordance with section 3.11. "Evaluation of Tumor Regression Grade (TRG)", and and the efficacy endpoints (PFS, RR, and OS) will be evaluated.
2. Modified tumor regression grade (mTRG)
   With the patients who underwent liver resection defined in section 11.3.4 "Liver Resection Rate" designated as the parent population, the relationships between the percentage of each grade calculated in accordance with section 3.12. "Evaluation of Modified Tumor Regression Grade (mTRG)" and the efficacy endpoints (PFS, RR, and OS) will be evaluated.
   The relationships between (1) TRG and the efficacy endpoints (PFS, RR, OS) will also be evaluated.
3. Dangerous halo (HALO)
   With the patients who underwent liver resection defined in section 11.3.4 "Liver Resection Rate" designated as the parent population, the relationships between the percentage of each grade calculated in accordance with section 3.13. "Dangerous Halo (HALO)", and the efficacy endpoints (PFS, RR, and OS) will be evaluated.
   The relationships with the number of days from the last day of treatment with bevacizumab or cetuximab to the day of resection will also be evaluated.
4. Sinusoidal obstruction syndrome (SOS)
   With the patients who underwent liver resection defined in section 11.3.4 "Liver Resection Rate" designated as the parent population, the relationships between the percentage of each grade calculated in accordance with section 3.14. "Evaluation of Sinusoidal Obstruction Syndrome (SOS) (Rubbia-Brandt grade)", and the efficacy endpoints (PFS, RR, and OS) will be evaluated. The relationships with treatment status and safety (surgery-related) will also be evaluated.
5. Other
   Apart from TRG, mTRG, HALO, and SOS, exploratory histopathological investigations will be performed as required, and evaluated with the patients who underwent liver resection defined in section 11.3.4. "Liver Resection Rate" designated as the parent population. As required, the relationship with efficacy endpoints (PFS, RR, OS), and the relationships with treatment status and safety (surgery-related) will also be evaluated.
6. Spleen Volume Index (SVI)[57,58]
   With the full analysis set (see 11.2.2) and patients who underwent liver resection defined in section 11.3.4 "Liver Resection Rate" designated as the parent populations, the relationships between the percentage change

from baseline in spleen volume at radiology review at four months and the efficacy endpoints (PFS, RR, and OS) will be evaluated. The relationships with treatment status and safety (surgery-related) will also be evaluated.

The relationships with (4) SOS and thrombocytopenia will also be evaluated.

7. Morphologic Response[37)]

   With the full analysis set (see 11.2.2) and patients who underwent liver resection defined in section 11.3.4 "Liver Resection Rate" designated as the parent populations, the relationships between the percentage of morphologic response (optimal response, incomplete respons and no response) which was evalusted and based on morphologic response criteria and the efficacy endpoints (PFS, RR, and OS) will also be evaluated.

11.5. Subgroup Endpoints

1. PFS, response rate, tumor shrinkage rate at 8 weeks, liver resection rate, R0 liver resection rate, TTF, and OS by number of liver metastases (single, 2-4, 5-10, 11 or more)
2. PFS, response rate, and tumor shrinkage rate at 8 weeks by R0 liver resection (R0, R1 or higher/unresected)
3. PFS, response rate, tumor shrinkage rate at 8 weeks, liver resection rate, R0 liver resection rate, TTF, and OS by primary lesions (yes, no) in synchronous liver metastasis
4. If an assessment of PD (progression) is made on the basis of diagnostic imaging undertaken during protocol treatment, the number of times surgery is performed, the number of R0 liver resections, and PFS with PD (progression) based on postoperative diagnostic imaging (including situations where another therapy has been provided as an ongoing treatment) defined as an event.

12. Identification of Source Data and Source Document Verification

12.1. Identification of Source Data

The EDC will be classified as source data only for the following variables recorded in the EDC system.

1. Comments by the investigator and subinvestigator
2. Treatment status: Reasons for treatment suspension, reasons for dose hold or change in total dose (applicable to dose reduction criteria or recalculation of total dose), reasons for delaying the proposed start of protocol chemotherapy, and reasons for delaying the proposed start of treatment with each investigational product
3. Special notes for blood sampling records
4. Observations of lesions: Target lesions (detailed site of lesion, long axis (short axis for lymph node metastases)), non-target lesions (presence of lesion, detailed site of lesion), new lesions (presence of lesion, detailed site of lesion), assessment of tumor shrinkage response, best overall response
5. Electrocardiograms (resting 12-lead): Abnormal findings and details
6. Chest X-rays: Abnormal findings and description
7. Head CT/MRI: Abnormal findings and description
8. Adverse events: Assessment of abnormal variations in laboratory values, seriousness, assessment of severity of adverse events, etc., assessment of causality, outcome, date of resolution/improvement
9. Study completion: Reason for discontinuation of treatment, reason for study discontinuation, report of death (causal relationship with investigational products)

12.2. Source Document Verification

The head of the study site and the investigator must make available source documents and all other study-related records in response to requests from the monitors, auditors, and other relevant staff whenever monitoring, auditing, or investigations by the ethics committee (or IRB) or the regulatory authorities are to be carried out.

All necessary measures must be put in place to ensure the protection of subject confidentiality during the direct inspection of source documents.

13. Statistical Analysis

13.1. Main Analysis and Criteria

The main objective of this study is to conduct an exploratory comparison of whether the primary endpoint, progression-free survival, is significantly longer for Group A (mFOLFOX plus bevacizumab) than for Group B (mFOLFOX plus cetuximab) in patients with liver-only metastases from colorectal cancer.

In the main analysis, the hazard ratio for Group B versus Group A will be estimated for the primary endpoint, progression-free survival, based on a stratified proportional hazard model using assignment adjustment factors other than study site. If the point estimate of the hazard ratio for Group B versus Group A is less than 1.00, it will be concluded that Group B is the more promising treatment, and conversely, if the point estimate of the hazard ratio is 1.00 or higher, it will be concluded that Group A is the more promising treatment. However, it is also possible that summarization using hazard ratios is inappropriate, for example, when the shapes of the survival curves in each group are substantially different and do not intersect. In such cases, the question of which treatment is more promising will be decided via a comprehensive assessment, taking into account the toxicity of each treatment. For reference purposes, estimates including the cumulative progression-free survival curves, median progression-free survival, and annual progression-free survival rate will be calculated using the Kaplan-Meier method.

13.2. Handling of Protocol Deviations and Violations

Statistical analyses will be performed in accordance with the statistical analysis plan that will be finalized before data locking, in accordance with the definitions in section 18.3. "Protocol Deviations and Violations", after discussions by the sponsor with the person responsible for statistical analysis and the study coordinating committee as required, and after approval by the efficacy and safety evaluation committee.

13.3. Planned Number of Enrollments, Enrollment Period, and Follow-up Period

Planned number of enrollments: A total of 120 patients in two groups (90 in each)

Enrollment period: May 2013 to April 2016 (three years)

Follow-up period: One years after the end of enrollment

Overall study period: May 2013 to April 2017 (four years)

13.4. Interim Analyses and Early Study Termination

No interim analyses will be performed.

13.5. Analysis of Secondary Endpoints, Exploratory Endpoints, and Subgroup Endpoints

13.5.1. Analysis of Secondary Endpoints of Safety

The secondary endpoints of safety are chemotherapy-induced adverse events (adverse reactions) and intraoperative and postoperative complications if liver resection is performed.

Intervals will be estimated using binomial exact confidence intervals, and as required, groups will be compared using Fisher's exact test.

13.5.2. Analysis of Secondary Endpoints, Exploratory Endpoints, and Subgroup Endpoints of Efficacy

The secondary and exploratory endpoints of efficacy are response rate, tumor shrinkage rate at eight weeks, liver resection rate, R0 liver resection rate, response duration, overall survival, QoL, pathologic endpoints (TRG, mTRG, HALO, SOS, others), SVI and morphologic response, which will be analyzed only in the final analysis.

The analytical method for the endpoint time to event will be the same as that for the primary endpoint,

progression-free survival. Fisher's exact test will be used for the inter-group comparison of response rates, and binomially distributed exact confidence intervals will be used for interval estimates.

The subgroup analyses defined in section 11.5 "Subgroup Endpoints" will be performed.

13.6. Final Analyses

After the end of the follow-up period, final analyses for all endpoints will be performed after the data have been verified in the final investigation. The final analysis results will be summarized by the person responsible for statistical analysis in a final analysis report, which will be submitted to the principal investigator.

14. Ethics

14.1. Regulatory Compliance Statement

This study will be conducted in compliance with the most recent versions of the Declaration of Helsinki (attached table), the protocol, Ethical Guidelines for Clinical Studies, and ICH-GCP.

14.2. Patient Protection

All researchers involved with this study will conduct the study in compliance with the Declaration of Helsinki.

14.3. Informed Consent

14.3.1. Explanations to Patients

Pursuant to the most recent version of the Declaration of Helsinki and prior to enrollment in the clinical study, the subinvestigator will hand each candidate personally, the patient information pamphlet approved by the site IRB, and provide a verbal explanation of the following matters.

1. That this study involves clinical research, and is different from general clinical practice
2. Explanations of the disease name, stage (degree of progression), and anticipated prognosis
3. Explanation of the standard therapy for the target disease
4. The design and justification (objective, significance, methodology, and number of enrollments, etc.) for this study
5. Description of the protocol therapy
   The drug names, methods of administration, doses, treatment cycles, and the overall duration of protocol treatment, etc.
6. Benefits that can be expected from the protocol treatment
   Life prolongation, tumor downsizing, symptomatic relief, etc.
7. Expected adverse events, complications, sequelae, and methods for dealing with them
   An explanation of the severities and frequencies of expected adverse events, including complications, sequelae, and treatment-related mortality, and methods for dealing with them
8. Alternative therapies
   The advantages and disadvantages of choosing the existing, standard treatment methods (including palliative care) and alternative therapies, including the details, benefits, and toxicities of standard therapies
9. Costs and compensation
   An explanation that all treatment-related costs will be covered by the health insurance system, that compensation in the event of health injury will be in line with routine practice in the normal clinical setting, and that such costs and compensation are the same as in normal clinical practice
10. Expected advantages and potential disadvantages
    An explanation of the benefits that could be enjoyed and the potential disadvantages that might be suffered by participating in the study
11. Direct inspection of clinical histories

An explanation about the acceptance of auditing, which includes accepting "that for quality control purposes, medical staff from other sites will directly inspect clinical histories, subject to permission from the head of the study site".

12. Refusal of consent and withdrawal of consent
    That the patient is free to refuse consent before participation in the study, and is free to withdraw consent at any time after having given consent, without prejudicing their treatment in any way
13. Protection of human rights
    That the best efforts will be made to maintain confidentiality of patient names and their personal information
14. Secondary use of data
    Subject to approval by the principal investigator, that data may be subject to secondary use (for example, for meta-analysis), unlinked to personal identifying information
15. Conflicts of interest and funding sources
    That conflicts of interest will be controlled and disclosed where necessary. An explanation on funding sources will be given.
16. Central ethics review
    That this study has been appropriately reviewed from the scientific and ethical standpoints by an independent, external organization.
17. Freedom to ask questions
    Patients will be informed of not only the subinvestigator's contact details, but also those of the principal investigator, and that they are free to ask questions on the study and the details of their treatment

14.3.2. Informed Consent

After checking that patients have received an explanation of the study and confirmed that they fully understand its details, they will be invited to participate in the study. If a patient consents to participate in the study, the name of the doctor who gave the explanation, the name of the patient who received the explanation, and the date on which consent was obtained will be recorded on the consent form, and the doctor and patient will each sign the form.

Two copies of the patient information pamphlet and consent form will be prepared, one will be handed to the patient, and the other retained at the study site. Originals will be stored with the medical records.

14.4. Protection of Personal Information and Patient Identification

Names of enrolled patients will not be disclosed by enrolling study sites to the data center.

Identification of and reference to enrolled patients will be made using the enrollment numbers issued at enrollment, their dates of birth, and their patient ID numbers. Information such as personal names that can directly identify patients without unauthorized third party access to the study site personnel or database will not be recorded in the data center database. Interactions involving the study sites, data center, and patient data will as a general principle, take place via the EDC.

14.4.1. Purpose of Use and Data to be Used, and Methods of Use of Personal Information

1. Purpose of use
   In this study, patient personal information will be used for ensuring that monitoring is being carried out appropriately.
2. Data to be used
   As an absolute minimum required for identification of and reference to patients, the data to be used will be as follows.
   Patient ID number (issued at each study site), date of birth, pathology specimen number (whenever required)

In brief, patient names, and personal information other than that listed above, will not be communicated to the data center or the study organization from the participating study site, and if disclosed in error, the information will be destroyed, whatever the recording medium, or rendered unreadable by masking or other appropriate processing means and then stored.

3. Usage method

Patient personal information and clinical records will be collected by the subinvestigators at each study site, who will enter it into the various CRFs via the EDC system. Personal information will not be transmitted via electronic mail.

14.4.2. On the secondary use of data

Subject to approval via review by the principal investigator and steering committee, the secondary use (for example, for meta-analysis) of data obtained in this study, unlinked to personal identifying information, may be possible

14.4.3. Safety Management Responsibilities

To minimize the risk of information leakage when personal information is used, safety management strategies will be devised in accordance with the regulations at each participating study site. The study sponsor EPS Corporation appropriately manages information in accordance with the information security management system standard, ISO/IEC 27001: 2005/JIS Q 27001: 2006.

14.4.4. Dealing with Requests for Disclosure of Patient Information

The person who responds to a request by a patient for disclosure of privacy-related information held by the data center will as a rule, be the researcher (subinvestigator) at the subject's study site.

14.5. Protocol Compliance

Research staff participating in this study will adhere to the study protocol, insofar as patient safety or human rights are not impaired.

14.6. Approval by the Study Site Ethics Committee (IRB)

14.6.1. Approvals at the Start of Study Participation

Before a site participates in this study, the study protocol and explanatory pamphlet for subjects must be approved by the ethics committee or institutional review board (IRB) of the study site. Once IRB approval has been given, the investigator will forward a copy of the IRB approval letter to the data center. The original IRB approval letter will be retained by the investigator.

14.6.2. Annual Updates of IRB Approval

Annual updates of the IRB review and approval for the study protocol and explanatory pamphlet for patients will be completed in accordance with regulations at each participating study site. IRBs will not be asked to submit an annual update approval document.

14.7. Protocol Changes

14.7.1. Classification of Protocol Changes

Before activation of any protocol amendment, an application for protocol revision must be submitted to the efficacy and safety evaluation committee for its approval.

Amendments to the protocol after approval by the study coordinating committee will be classified into two categories, amendments and revisions, and all applications by researchers to the committee will be classified as "applications for revision", with the classification of amendments and revisions to be done by the efficacy and safety

evaluation committee. Supplementary explanations that do not fit into the definition of amendments to the protocol will be classified as memoranda. Definitions and classifications will be as follows.

1. Amendment

   Partial amendments to the protocol relating to the primary endpoint of the study or with the potential to increase the risk to patients participating in the study. Approval by the efficacy and safety evaluation committee and each study site is required.

   Approval by the principal investigator and study coordinating committee members is required before submitting an application to the efficacy and safety evaluation committee. The date of approval by the efficacy and safety evaluation committee and the effective date will be documented on the cover page of the protocol.

2. Revision

   Protocol amendments other than those above that are not related to the primary endpoint of the study or without the potential to increase the risk to patients participating in the study.

   The approval of the efficacy and safety evaluation committee is required. Approval by the principal investigator and study coordinating committee members is required before submitting an application to the efficacy and safety evaluation committee.

   Approval by the study site will proceed in accordance with the arrangements at each site.

   The date of approval by the efficacy and safety evaluation committee and the effective date will be documented on the cover page of the protocol.

3. Memoranda

   Supplementary explanations to the protocol that will be distributed by the principal investigator to persons involved in the study, which aim to reduce the variation in documentary interpretation and draw particular attention to issues, and that are not changes to the protocol. No particular form is specified.

   Approval by the principal investigator and study coordinating committee members is required before distribution. Reporting to the efficacy and safety evaluation committee is required before and promptly after distribution. Documentation on the cover page of the protocol is not required.

14.7.2. Study Site IRB Approval at Amendment or Revision of the Protocol

If amendment of the study protocol or the information pamphlet for subjects is done with the approval of the efficacy and safety evaluation committee during the study, the amended study protocol and information pamphlet must be approved by the ethics committee (or IRB) at each study site.

Whenever the change is a revision rather than amendment, the question of whether or not review and approval is required by the ethics committee (or IRB) at each study site will be resolved in accordance with the arrangements at each study site.

Once the IRB has given approval for an amendment, the investigator will forward a copy of the IRB approval letter to the data center. The original IRB approval letter will be retained by the investigator and a copy retained by the data center.

15. Conflicts of Interest and Funding Sources

15.1. Conflicts of Interest

Decision-making for the planning, conduct, and publication of this study will be done via a study organization that includes the principal investigator of the study. Each researcher will appropriately manage conflicts of interest by measures such as compliance with the conflict of interest management policy in force at their respective academic societies and affiliated study site, and shall make appropriate disclosures to meet the requirements of the academic society or medical journal that plans to publish the study results.

15.2. Funding Sources and Financial Relationships

This study will be conducted by EPS Corporation and the researchers, with support provided by Chugai Pharmaceutical Co., Ltd. The participating study sites will be paid by EPS Corporation for conducting the study, comprising amounts which will be proportional to the number of subjects enrolled. Products manufactured and marketed by Chugai Pharmaceutical Co., Ltd. will be used in this study, but nothing in this arrangement will influence the study results.

16. Study Costs and Compensation

16.1. Costs Relating to Study Treatment

This study will be conducted within the scope of normal health insurance arrangements, and expenses for requirements such as observations, tests and drugs during the study period will be covered by the health insurance system.

16.2. Compensation for Injury

If an injury to health attributable to the conduct of the study arises, the subinvestigator and participating study site shall take action to ensure that appropriate treatment is provided and other necessary measures are instituted. However, health insurance shall be applicable to the treatment provided and monetary compensation will not be paid.

17. Ownership of Research Results

The results generated in this study will become the property of Chugai Pharmaceutical Co., Ltd. and all participating study sites. However, intellectual property rights concerned with pharmaceutical products manufactured and marketed by Chugai Pharmaceutical Co., Ltd. will belong to Chugai Pharmaceutical Co., Ltd.

18. Monitoring

Monitoring activities will include central monitoring and onsite monitoring, as required.

18.1. Central Monitoring

Central monitoring will involve checking that the research has been conducted safely and in accordance with the protocol, and that data have been properly collected, based on the data entered in the case report forms collected via the EDC. As a general principle, central monitoring will be performed twice a year (starting from enrollment of the first patient), periodic monitoring reports will be prepared, and submitted to the principal investigator.

Periodic monitoring reports can be used for evaluation by the study coordinating committee and feedback on problems and review purposes in regular or educational briefing sessions.

1. Patient recruitment status: Number of enrollments—cumulative/by period, by group/study site
2. Patient eligibility: Ineligible/potentially ineligible patients: By group and study site
3. Pre-treatment demographic and clinical characteristics: Group
4. By protocol surgery performed/not performed: All enrolled patients, status by group will not be monitored
5. By protocol treatment in progress/discontinued, reason for discontinuation: Status by group will not be monitored
6. Protocol deviations: Group/site
7. Serious adverse events: Group/site
8. Adverse reactions/adverse events: Group
9. Overall survival, progression-free survival: All enrolled patients
10. Other problems that are concerned with study progress and safety

18.2. Onsite Monitoring

Onsite monitoring will include sampling of about 10% of sites, and source document verification (SDV) by sampling.

By comparison against source documents, monitoring will confirm that the research has been conducted safely and in accordance with the protocol, and that data have been properly collected. The frequency and procedures for onsite monitoring will be in accordance with the separately prepared monitoring plan.

1. Patient recruitment status: Number of enrollments—cumulative/by period, by group/study site
2. Patient eligibility: Ineligible/potentially ineligible patients: By group and study site
3. Pre-treatment demographic and clinical characteristics: Group
4. By protocol treatment in progress/discontinued, reason for discontinuation: Group/status
5. Protocol deviations: Group/site
6. Serious adverse events: Group/site
7. Adverse reactions/adverse events: Group
8. Overall survival, progression-free survival: All enrolled patients
9. Other problems that are concerned with study progress and safety

18.3. Protocol Deviations and Violations

Any of the following that are not conducted in accordance with the requirements of the protocol will be regarded as protocol deviations: treatments such as drug administration and surgical resection, laboratory tests, and evaluations of toxicity and efficacy. Events will be classified as deviations or violations by the study coordinating committee. The handling of the particular patient or tabulation and analysis of data will be discussed by the study coordinating committee, and approved by the efficacy and safety evaluation committee.

1. Violations

   Deviations from the requirements of the protocol that are clinically inappropriate and meet the criteria below will be regarded as violations.

   1) Affect the evaluation of the study endpoints
   2) Are attributable to the subinvestigator/site
   3) Intentional or systematic
   4) The severity of risk or deviation is pronounced

2. Deviations

   Deviations that do not meet the requirements of either (1) or the acceptable limits in (3)

   If multiple specific deviations are observed, they should be documented at publication.

   At monitoring, deviations should be classified into one of the following categories.

   1) Deviation: Should be reduced because it is undesirable
   2) Deviation (unavoidable): Not sufficient to require active steps to reduce
   3) Deviation (clinically acceptable): A deviation that positively suports the assessment of the subinvestigator or site

3. Acceptable Limits for Deviations

   Ultimately acceptable limits will be established after completion of the study (study coordinating committee).

   Deviations within pre-specified acceptable limits will not be included in monitoring reports.

   Deviations concerned with adverse events that are informed by the package insert for each drug.

19. Study Quality Control and Quality Assurance

19.1. Quality Control

The investigator will implement the following controls to ensure the accuracy, consistency, completeness, and reliability of data.

1. When initiating the clinical study, the investigator will brief their staff thoroughly on the study protocol and other documents, with the aim of ensuring their proper comprehension and standardizing assessments and evaluations.
2. The investigator or the subinvestigator will prepare case report forms in accordance with the requirements of the protocol. If there is a deviation from the protocol, the details and relevant reasons will be documented in medical records and elsewhere.
3. Deviations that are based on source documents in case report forms must not be inconsistent with the source documents. If there is any inconsistency with the source documents, the relevant reasons will be documented in medical records and elsewhere.
4. To eliminate violations of inclusion and exclusion criteria, the investigator or subinvestigator will enroll patients after thoroughly checking the demographic and clinical characteristics of the enrolled subjects. The data center will also check the eligibility of the enrolled subjects.
5. To eliminate variations in assessments and evaluations, assessments concerned with radiology review and pathology review will be performed by the CRO for supporting the central radiology review and the pathology evaluation committee.

19.2. Quality Assurance

1. Routine monitoring will be performed to ascertain compliance with the sponsor's protocol, standard operating procedures, and ICH-GCP, and auditing will be performed to evaluate that the quality control activities of the study are being carried out independently and separately. The auditor will conduct audits of the participating study sites in accordance with the separately-established "Audit SOP" and "Audit Plan".

20. Data Handling And Archiving

20.1. Preparation, Amendment, and Correction of Case Report Forms (CRF)

Case report forms will be prepared for all enrolled subjects.

The investigator or the subinvestigator will prepare and amend or revise the information in case report forms in accordance with the sponsor's EDC input manual.

20.2. Management of Electronic Data Storage

In addition to using an electronic data processing system to store the entries made in case report forms as electronic data, the sponsor will perform the following tasks.

1. Keep records of data amendments if the electronic data is amended (date of amendment, by whom amended).
2. Control system security.
3. Undertake appropriate data backup.

20.3. Retention of Records

Records will be retained in accordance with the standard operating procedures set down by the ethics committee or IRB of each study site.

20.3.1. Investigators

Records concerned with patient consent, basic data for report preparation (test data, etc.), approval letters issued by ethics committee or IRBs, and documentary records prepared by participating study sites will be retained by the investigator (subinvestigator). Documents will be retained for five years after the discontinuation or completion of the

study overall.

20.3.2. Documents to be Retained

The sponsor will retain all documents and records pertaining to the study that should be retained by the sponsor in accordance with the requirements of the relevant standard operating procedures.

21. Study Organization

21.1. Principal Investigators

Professor Yoshihiko Maehara Graduate School of Medical Sciences, Kyushu University
Department of Surgery II, Department of Surgery and Science
3-1-1 Maidashi, Higashi-ku, Fukuoka, Fukuoka Prefecture 812-8582.
Tel: 092-642-1151 (main)

Professor Naohiro Tomita
Division of Lower Gastrointestinal Surgery, Department of Surgery, Hyogo College of Medicine
1-1 Mukogawa-cho, Nishinomiya, Hyogo Prefecture 663-8501
Tel: 0798-45-6111 (main)

Professor Ichinosuke Hyodo
Gastroenterology, Graduate School of Comprehensive Human Sciences,
Institute of Clinical Medicine, University of Tsukuba
1-1-1 Tennnodai, Tsukuba, Ibaraki Prefecture 305-8577
Tel: 029-853-3109 (direct)

Professor Michiaki Unno
Division of Gastroenterological Surgery, Tohoku University Graduate School of Medicine
1-1 Seiryo-machi, Aoba-ku, Sendai, Miyagi Prefecture 980-8674. Tel: 022-717-7000 (main)
Tel: 022-717-7000 (main)

21.2. Study Medical Advisor

Professor Kenichi Suihara
Division of Systemic Organ Regulation, Tokyo Medical and Dental University Graduate School
Department of Surgical Oncology, Digestive and Metabolic Disease
1-5-45 Yushima, Bunkyo-ku,Tokyo 113-8519
Tel: 03-5803-5261

21.3. Study Secretariat

Professor Yoshihiko Maehara
Graduate School of Medical Sciences, Kyushu University
Department of Surgery II, Department of Surgery and Science
3-1-1 Maidashi, Higashi-ku, Fukuoka, Fukuoka Prefecture 812-8582.
Tel: 092-642-5466

21.4. Study Coordination Committee (Japanese alphabetical order)

Yasunori Emi	Kyushu University
Hiroyuki Uetake	Tokyo Medical and Dental University
Yu Katayose	Tohoku University
Kei Muro	Aichi Cancer Center Hospital

21.5. Protocol Preparation Committee

Chair

Kei Muro Aichi Cancer Center Hospital

Members (Japanese alphabetical order)

Hiroyuki Uetake Tokyo Medical and Dental University
Eiji Oki Kyushu University
Yu Katayose Tohoku University
Takao Takahashi Gifu University
Masashi Noda Hyogo College of Medicine
Kensei Yamaguchi Saitama Cancer Center
Takayuki Yoshino National Cancer Center Hospital East

21.6. Steering Committee

Chair

Keiichi Takahashi Tokyo Cancer and Infectious Diseases Center Komagome Hospital

Members (Japanese alphabetical order)

Michio Itabashi Tokyo Women's Medical University
Hideki Ohdan Hiroshima University
Yoshihiro Kakeji Kobe University
Yoshito Komatsu Hokkaido University
Yoshiharu Sakai Kyoto University
Mitsuo Shimada Tokushima University
Akinobu Taketomi Hokkaido University
Kuniya Tanaka Yokohama City University
Tadamichi Denda Chiba Cancer Center
Kenichi Hakamada Hirosaki University
Eishi Baba Kyushu University
Hideo Baba Kumamoto University
Toshiyoshi Fujiwara Okayama University
Kei Muro Aichi Cancer Center Hospital
Akio Yamaguchi University of Fukui
Kensei Yamaguchi Saitama Cancer Center
Kazuhiro Yoshida Gifu University
Hiroyuki Yoshitome Chiba University
Takayuki Yoshino National Cancer Center Hospital East
Go Wakabayashi Iwate Medical University

21.7. Efficacy and Safety Evaluation Committee

Chair

Kenjiro Kotake Tochigi Cancer Center

Members

Yoshinori Miyata Saku Central Hospital

Akio Saiura Cancer Institute Ariake Hospital

21.8. Contract Research Organization supporting the central radiology review

Hiromasa Suzuki Clinical Development Division, Micron Inc.
Sumitomofudosan Marunouchi Bldg, 3-8-1 Marunouchi, Chiyoda-ku, Tokyo 100-0005
Tel: 03-6268-0308
Fax: 03-6268-0309

21.9. Pathology Review Committee (Japanese alphabetical order)

Shinichi Aishima Kyushu University

Kazuyuki Ishida Iwate Medical University

Joji Imura Toyama University

Michiie Sakamoto Keio University

21.10. Person Responsible for Statistical Analysis

Takaharu Yamanaka National Cancer Center Hospital East

21.11. Sponsor

EPS Corporation
2-3-19 Koraku, Bunkyo-ku,Tokyo 112-0004
Tel: 03-5684-7797 (main)

Data center: Responsible person Tatsumi Shimizu
DM Center 1, Clinical Information Division
2-3-19 Koraku, Bunkyo-ku,Tokyo 112-0004
Tel: 03-5684-7767
E-mail: prj-atomdc@eps.co.jp

Monitoring Responsible person Yoichi Narita
Clinical Coordinating Department, Clinical Coordinating Center

Audit Responsible person Katsumi Hirano
Quality Assurance Department, Quality Management Division

21.12. Data Center

Tatsumi Shimizu DM Center 1, Clinical Information Division, EPS Corporation
2-3-19 Koraku, Bunkyo-ku,Tokyo 112-0004
Tel: 03-5684-7767
E-mail: prj-atomdc@eps.co.jp

22. Announcement of Study Results

The principal investigators will, together with the investigators participating in the study, discuss the study authors and announcement medium, as set out in the study organization arrangements.

22.1. Agreement on Authorship for Article Publications and Conference Presentations

22.1.1. Article Publication

Authorship of an article announcing the study results will be decided in proportion to the degree of contribution to the study (design, planning, management, enrollment, analysis)

All coauthors shall review the article and agree on its contents before submission.

22.1.2. Conference Presentations

The person chosen to announce the study results in a conference will be decided in proportion to their contribution to the study (design, planning, management, enrollment, analysis)

22.1.3. Media for Announcement of the Results

The study results will be presented at a key Japanese or international conference.

The information contained in the conference proceedings will be ultimately compiled into an article and submitted to a specialist journal.

23. References

1. Cancer Statistics in Japan—2010: http://ganjoho.jp/public/statistics/backnumber/2010_jp.html
2. JSCCR 2005 Guidelines for the Treatment of Colorectal Cancer. Colorectal Cancer Study Group (ed.). Kanehara Shuppan, 2005.
3. Kato T, Yasui K, et al. Therapeutic results for hepatic metastasis of colorectal cancer with special reference to effectiveness of hepatectomy; Analysis of prognostic factors for 763 cases recorded at 18 institutions. Dis Colon Rectum 2003; 46: S22-31.
4. Adam R, Delvart V, et al. Rescue surgery for unresectable colorectal liver metastases downstaged by chemotherapy: a model to predict long-term survival. Ann Surg 2004; 240: 644-57.
5. Folprecht G, Grothey A, et al. Neoadjuvant treatment of unresectable colorectal liver metastases: correlation between tumour response and resection rates. Ann Oncol 2005; 16: 1311–9.
6. NCCN Guidelines Colon Cancer Version 1. 2013：
http://www.nccn.org/professionals/physician_gls/pdf/colon.pdf
7. Schmoll HJ, Van Cutsem E, et al. ESMO Consensus Guidelines for management of patients with colon and rectal cancer. A personalized approach to clinical decision making. Annals of Oncology 23: 2479-516, 2012.
8. Nordlinger B, Van Cutsem E, et al. Combination of surgery and chemotherapy and the role of targeted agents in the treatment of patients with colorectal liver metastases: recommendations from an expert panel. Ann Oncol 2009; 20: 985–92.
9. Giacchetti S, Itzhaki M, et al. Long-term survival of patients with unresectable colorectal cancer liver metastases following infusional chemotherapy with 5-fluorouracil, leucovorin, oxaliplatin and surgery. Ann Oncol 1999; 10: 663–9.
10. Kopetz S, Chang GJ, et al. Improved survival in metastatic colorectal cancer is associated with adoption of hepatic resection and improved chemotherapy. J Clin Oncol 2009; 27: 3677-83.
11. Adam R, Barroso E, et al. Impact of the type and modalities of preoperative chemotherapy on the outcome of liver resection for colorectal metastases. J Clin Oncol 2011; 29: abstr 3519.
12. Nordlinger B, Sorbye H, et al. Perioperative chemotherapy with FOLFOX4 and surgery versus surgery alone for resectable liver metastases from colorectal cancer (EORTC Intergroup trial 40983): a randomised controlled trial. Lancet 2008; 371: 1007-1016.
13. Nordlinger B, Sorbye H, et al. EORTC liver metastases intergroup randomized phase III study 40983: Long-term survival results. J Clin Oncol 2012; 30: abstr 3508.
14. Okines A, del Puerto O, et al. Surgery with curative-intent in patients treated with first-line chemotherapy plus bevacizumab for metastatic colorectal cancer. First BEAT and the randomised phase-III NO16966 trial. Br J Cancer 2009; 101: 1033-8.
15. Saltz LB, Clarke S, et al. Bevacizumab in combination with oxaliplatin-based chemotherapy as first-line therapy in metastatic colorectal cancer: A randomized phase III study. J Clin Oncol 2008; 26: 2013-9.
16. Van Cutsem E, Köhne CH, et al. Cetuximab and chemotherapy as initial treatment for metastatic colorectal cancer. N Engl J Med 2009; 360: 1408-17.
17. Bokemeyer C, Bondarenko I, et al. Fluorouracil, leucovorin, and oxaliplatin with and without cetuximab in the first-line treatment of metastatic colorectal cancer. J Clin Oncol 2009; 27: 655-8.
18. Alberts SR, Horvathet WL, et al. Oxaliplatin, Fluorouracil, and Leucovorin for Patients With Unresectable Liver-Only Metastases From Colorectal Cancer: A North Central Cancer Treatment Group Phase II Study. J Clin Oncol 2005; 23: 9243-49.
19. Wong R, Cunningham D, et al. A multicentre study of capecitabine, oxaliplatin plus bevacizumab as perioperative treatment of patients with poor-risk colorectal liver-only metastases not selected for upfront resection. Ann Oncol 2011; 22: 2042–8.
20. Folprecht G, Gruenberger T, et al. Tumour response and secondary resectability of colorectal liver metastases

following neoadjuvant chemotherapy with cetuximab: the CELIM randomised phase 2 trial. Lancet Oncol 2010; 11: 38-47.

21. Garufi C, Torsello A, et al. Cetuximab plus chronomodulated irinotecan, 5-fluorouracil, leucovorin and oxaliplatin as neoadjuvant chemotherapy in colorectal liver metastases: POCHER trial. Br J Cancer 2010; 103: 1542-47.
22. Portier G, Elias D, et al. Multicenter randomized trial of adjuvant fluorouracil and folinic acid compared with surgery alone after resection of colorectal liver metastases: FFCD ACHBTH AURC 9002 Trial. J Clin Oncol 2006; 24: 4976-82.
23. Mitry E, Fields ALA, et al. Adjuvant chemotherapy after potentially curative resection of metastases from colorectal cancer: A pooled analysis of two randomized trials. J Clin Oncol 2008; 26: 4906-11.
24. Ychou M, Hohenberger W, et al. A randomized phase III study comparing adjuvant 5-fluorouracil/folinic acid with FOLFIRI in patients following complete resection of liver metastases from colorectal cancer. Ann Oncol 2009; 20: 1964–70.
25. Kanemitsu Y, Kato T, et al. A randomized phase II/III trial comparing hepatectomy followed by mFOLFOX6 with hepatectomy alone as treatment for liver metastasis from colorectal cancer: Japan Clinical Oncology Group Study JCOG0603. Jpn J Clin Oncol 2009; 39: 406–9.
26. Tournigand C, Andre T, et al. FOLFIRI followed by FOLFOX6 or the reverse sequence in advanced colorectal cancer: a randomized GERCOR study. J Clin Oncol 2004; 22: 229-37.
27. Vauthey JN, Pawlik TM, et al. Chemotherapy regimen predicts seatohepatitis and an increase in 90-Day mortality after surgery for hepatic colorectal metastases. J Clin Oncol 2006; 24: 2065-72.
28. Rubbia-Brandt L, Audard V, et al. Severe hepatic sinusoidal obstruction associated with oxaliplatin-based chemotherapy in patients with metastatic colorectal cancer. Ann Oncol 2004; 15: 460-6.
29. Kleiner DE, Brunt EM, et al. Design and validation of a histological scoring system for nonalcoholic fatty liver disease. Hepatology 2005; 41: 1313-21.
30. Ribero D, Wang H, et al. Bevacizumab improves pathologic response and protects against hepatic injury in patients treated with oxaliplatin-based chemotherapy for colorectal liver metastases. Cancer 2007; 110: 2761–7.
31. Kanwal PSR, Karime K, et al. Effect of bevacizumab on rate of oxaliplatin-induced thrombocytopenia and splenomegaly. J Clin Oncol 2012; 30: abstr 3544.
32. Blazer DG, Kishi Y, et al. A new outcome endpoint after resection of hepatic colorectal metastases. J Clin Oncol 2008; 26: 5344-51.
33. Rubbia-Brandt L, Giostra E, et al. Importance of histological tumor response assessment in predicting the outcome in patients with colorectal liver metastases treated with neo-adjuvant chemotherapy followed by liver surgery. Ann Oncol 2007; 18: 299–304.
34. Mandard AM, Dalibard FF, et al. Pathologic assessment of tumor regression after preoperative chemoradiotherapy of esophageal carcinoma. Cancer 1994; 73: 2680-6.
35. Klinger M, Tamandl D, et al. Bevacizumab improves pathological response of colorectal cancer liver metastases treated with XELOX/FOLFOX. Ann Surg Oncol 2010; 17: 2059–65.
36. Li Chang HH, Leeper WR, et al. Infarct-like necrosis: A distinct form of necrosis seen in colorectal carcinoma liver metastases treated with perioperative chemotherapy. Am J Surg Pathol 2012; 36: 570–6.
37. Chun YS, Vauthey JN, et al. Association of computed tomography morphologic criteria with pathologic response and survival in patients treated with bevacizumab for colorectal liver metastases. JAMA 2009; 302: 2338-44.
38. Maughan TS, Adams RA, et al. Addition of cetuximab to oxaliplatin-based first-line combination chemotherapy for treatment of advanced colorectal cancer: results of the randomised phase 3 MRC COIN trial. Lancet 2011; 377: 2103–14.

39. Buyse M, Burzykowski T, et al. Progression-free survival is a surrogate for survival in advanced colorectal cancer. J Clin Oncol 2007; 25: 5218-24.
40. Tang PA, Bentzen SM, et al. Surrogate end points for median overall survival in metastatic colorectal cancer: Literature-based analysis from 39 randomized controlled trials of first-line chemotherapy. J Clin Oncol 2007; 25: 4562-68.
41. Hurwitz H, YI J, et al. The clinical benefit of bevacizumab in metastatic colorectal cancer is independent of K-ras mutation status: Analysis of a phase III study of bevacizumab with chemotherapy in previously untreated metastatic colorectal cancer. The Oncologist 2009; 14: 22–8.
42. Tol J, Koopman M, et al. Chemotherapy, bevacizumab, and cetuximab in metastatic colorectal cancer. N Engl J Med 2009; 360: 563-72.
43. Hecht JR, Mitchell E, et al. A randomized phase IIIB trial of chemotherapy, bevacizumab, and panitumumab compared with chemotherapy and bevacizumab alone for metastatic colorectal cancer. J Clin Oncol 2008; 27: 672-80.
44. Díaz-Rubio E, Gómez-España A, et al. First-line XELOX plus bevacizumab followed by XELOX plus bevacizumab or single-agent bevacizumab as maintenance therapy in patients with metastatic colorectal cancer: The phase III MACRO TTD Study. Oncologist 2012; 17: 15-25.
45. Tveit KM, Guren T, et al. Phase III trial of cetuximab with continuous or intermittent fluorouracil, leucovorin, and oxaliplatin (Nordic FLOX) versus FLOX alone in first-line treatment of metastatic colorectal cancer: The NORDIC-VII study. J Clin Oncol 2012; 30: 1755-1762.
46. Köhne CH, Cunningham D, et al. Clinical determinants of survival in patients with 5-fluorouracilbased treatment for metastatic colorectal cancer: Results of a multivariate analysis of 3825 patients. Ann Oncol 2002; 13: 308-17.
47. Kabbinavar FF, Irl C, et al. Bevacizumab improves the overall and progression-free survival of patients with metastatic colorectal cancer treated with 5-fluorouracil-based regimens irrespective of baseline risk. Oncology 2008; 75: 215-23.
48. Tsai M, Su Y, et al. Clinicopathological feature and prognosis in respectable synchronous and metachronous colorectal liver metastases. Ann Surg Oncol 2007; 14: 786-94.
49. Ferrand F, Malka D, et al. Impact of primary tumour resection on survival of patients with colorectal cancer and synchronous metastases treated by chemotherapy: Results from the multicenter, randomised trial Fe´de´ration Francophone de Cance´rologie Digestive 9601. Eur J Cancer 2012 Epub ahead of print.
50. Venderbosch S, de Wilt JH, et al. Prognostic value of resection of primary tumor in patients with Stage IV colorectal cancer: retrospective analysis of two randomized studies and a review of the literature. Ann Surg Oncol 2011; 18: 3252–3260.
51. Beppu T, Sakamoto T, et al. A nomogram predicting disease-free survival in patients with colorectal liver metastases treated with hepatic resection: multicenter data collection as a Project Study for Hepatic Surgery of the Japanese Society of Hepato-Biliary-Pancreatic Surgery. J Hepatobiliary Pancreat Sc 2012; 19: 72-84.
52. Saiura A, Yamamoto J, et al. Liver resection for multiple colorectal liver metastases with surgery up-front approach: bi-institutional analysis of 736 consecutive cases. World J Surg 2012; 36: 2171–2178.
53. Mentha G, Terraz S, et al. Dangerous halo after neoadjuvant chemotherapy and two-step hepatectomy for colorectal liver metastases. Br J Surg 2009; 96: 95-103.
54. 幕内雅敏，高山忠利，他．肝硬変合併肝癌治療の strategy．外科診療 1987; 29: 1530-6.
55. Ward WL, Hahn EA, et al. Reliability and validity of the Functional Assessment of Cancer Therapy-Colorectal (FACT-C) quality of life instrument. Qual Life Res 1999; 8: 181-95.
56. Eisenhauer EA, Therasse P, et al. New response evaluation criteria in solid tumours: Revised RECIST guideline (version 1.1). Eur J Cancer 2009; 45: 228-47.
57. Overman MJ, Maru DM, et al. Oxaliplatin-mediated increase in spleen size as a biomarker for the development

of hepatic sinusoidal injury. J Clin Oncol 2010; 28: 2549-55.

58. Miura K, Nakano H, et al. Splenomegaly in FOLFOX-naı¨ve stage IV or recurrent colorectal cancer patients due to chemotherapy-associated hepatotoxicity can be predicted by the aspartate aminotransferase to platelet ratio before chemotherapy. Int J Clin Oncol 2011; 16: 257–63.
59. Wierzbicki R, Jonker DJ, et al. A phase II, multicenter study of cetuximab monotherapy in patients with refractory, metastatic colorectal carcinoma with absent epidermal growth factor receptor immunostaining. Invest New Drugs 2011; 29: 167-74.
60. Douillard JY, Oliner KS, et al. Panitumumab-FOLFOX4 treatment and RAS mutations in colorectal cancer. N Engl J Med 2013; 369: 1023-24.
61. Peeters M, Oliner KS, et al. Analysis of KRAS/NRAS mutations in phase 3 study 20050181 of panitumumab (pmab) plus FOLFIRI versus FOLFIRI for secondline treatment (tx) of metastatic colorectal cancer (mCRC). J Clin Oncol 2014; 32: abstr LBA387.
62. Patterson SD, Peeters M, et al. Comprehensive analysis of KRAS and NRAS mutations as predictive biomarkers for single agent panitumumab (pmab) response in a randomized, phase III metastatic colorectal cancer (mCRC) study (20020408). J Clin Oncol 2013; 31: abstr 3617.
63. Bokemeyer C, Kohne CH, et al. Treatment outcome according to tumor RAS mutation status in OPUS study patients with metastatic colorectal cancer (mCRC) randomized to FOLFOX4 with/without cetuximab. J Clin Oncol 2014; 32: abstr 3505.
64. Ciardiello F, Lenz HJ, et al. Treatment outcome according to tumor RAS mutation status in CRYSTAL study patients with metastatic colorectal cancer (mCRC) randomized to FOLFIRI with/without cetuximab. J Clin Oncol 2014; 32: abstr 3506.
65. Karthaus M, Schwartzberg L, et al. Updated overall survival (OS) analysis of novel predictive KRAS/NRAS mutations beyond KRAS exon 2 in PEAK: A 1st-line phase 2 study of FOLFOX6 plus panitumumab (pmab) or bevacizumab (bev) in metastatic colorectal cancer (mCRC). Eur J Cancer 2013; 49: S516.
66. Stintzing S, et al. Independent radiological evaluation of objective response, early tumor shrinkage, and depth of response in FIRE-3 (AIO KRK-0306) in the final RAS evaluable population. ESMO 2014 LBA11.
67. Lenz HJ, et al. CALGB/SWOG 80405: Phase III trial of irinotecan/irinotecan/5-FU/leucovorin (FOLFIRI) or oxaliplatin/5-FU/leucovorin (mFOLFOX6) with bevacizumab (BV) or cetuximab (CET) for patients (pts) with untreated metastatic adenocarcinoma of the colon or rectum (mCRC): Expanded RAS analyses. ESMO 2014 501O.
68. Patterson SD, Cohen N, et al. Prospective-retrospective biomarker analysis for regulatory consideration: white paper from the industry pharmacogenomics working group. Pharmacogenomics 2011; 12: 939-51.

24. Appendices

- Package Inserts
- Patient information sheet and informed consent form
- Declaration of Helsinki (Japanese translation by the Japan Medical Association)
