# Supplementary material for: Randomised phase II trial of mFOLFOX6 plus bevacizumab versus mFOLFOX6 plus cetuximab as first-line treatment for colorectal liver metastasis (ATOM trial)
Source: Br J Cancer. 2019 Jul 9;121(3):222–9. doi: 10.1038/s41416-019-0518-2 (PMC6738101; doi:10.1038/s41416-019-0518-2)
Supplement: Supplementary file 3 — List of name all institutional committees [file 41416_2019_518_MOESM3_ESM.docx]

Ethical subject was approved by the review board of all participated institutions. The name of institutions were listed following

Saiseikai Fukuoka General Hospital

Seirei Hamamatsu General Hospital

Sano Hospital

Yamaguchi University Hospital

National Hospital Organization Kobe Medical Center

JCHO Shimonoseki Medical Center

Hamamatsu Medical Center

Hyogo College of Medicine

Hakodate Goryoukaku Hospital

Oita Red Cross Hospital

Iizuka Hospital

Kyoto-Katsura Hospital

JCHO Kyushu Hospital

Chugoku Central Hospital

Ishinomaki Red Cross Hospital

National Hospital Organization Sagamihara National Hospital

Kumamoto University Hospital

Yokohama City University Hospital

Naha City Hospital

Sasebo City General Hospital

Iwaki Kyouritsu Hospital

Kyushu University Hospital

Gifu University Hospital

Kyushu Central Hospital of the Mutual Aid Association of Public School Teachers

Okayama University Hospital

Teikyo University Chiba Medical Center

FukuiKen Saiseikai Hospital

Hirosaki University Hospital

Fukui Prefectural Hospital

Nagoya City Hospital

National Hospital Organization Kyushu Medical Center

Tohoku University Hospital

Aichi Cancer Center Hospital

National Hospital Organization Nagoya Medical Center

University of Fukui Faculty of Medical Sciences

Nagasaki University Hospital

Tokyo Medical and Dental University

Kurume University

Kagoshima University Hospital

Kobe City Medical Center General Hospital

Gunma Prefectural Cancer Center

Kobe University Hospital

Hokkaido University Hospital

Kyoto University Hospital

Toho University Omori Medical Center

Hiroshima University Hospital

Japanese Red Cross Society Himeji Hospital

Hitachi General Hospital

Saiseikai Kumamoto Hospital

Omagari Kousei Medical Center

National Hospital Organization Tsuruga Medical Center

Tokyo Metropolitan Cancer and Infectious diseases Center Komagome Hospital

Steel Memorial Yawata Hospital

Kagawa Medical School Hospital

Handa City Hospital

NTT-East Sapporo Hospital

Kushiro Rosai Hospital

Kindai University

Niigata Cancer Center hospital

University of Tsukuba Hospital

Asahi General Hospital

Murakami Memorial Hospital

Meiwa General Hospital
